# Supplementary material for: Evaluation of the Reliability and the Performance of Magnetic Resonance Imaging Radiomics in the Presence of Randomly Generated Irrelevant Features for Prostate Cancer
Source: Diagnostics (Basel). 2023 Dec 1;13(23):3580. doi: 10.3390/diagnostics13233580 (PMC10705874; doi:10.3390/diagnostics13233580)
Supplement: Supplementary file 1 [file diagnostics-13-03580-s001.zip › diagnostics-2673699-supplementary.pdf]

Supplementary Table S1. Showing the feature index with the feature name in Dataset 1.

index	Feature Name (Song Dataset)
1	label
2	ADC_general_info_VolumeNum
3	ADC_general_info_VoxelNum
4	ADC_original_firstorder_10Percentile
5	ADC_original_firstorder_90Percentile
6	ADC_original_firstorder_Energy
7	ADC_original_firstorder_Entropy
8	ADC_original_firstorder_InterquartileRange
9	ADC_original_firstorder_Kurtosis
10	ADC_original_firstorder_Maximum
11	ADC_original_firstorder_Mean
12	ADC_original_firstorder_MeanAbsoluteDeviation
13	ADC_original_firstorder_Median
14	ADC_original_firstorder_Minimum
15	ADC_original_firstorder_Range
16	ADC_original_firstorder_RobustMeanAbsoluteDeviation
17	ADC_original_firstorder_RootMeanSquared
18	ADC_original_firstorder_Skewness
19	ADC_original_firstorder_TotalEnergy
20	ADC_original_firstorder_Uniformity
21	ADC_original_firstorder_Variance
22	ADC_original_glcm_Autocorrelation
23	ADC_original_glcm_ClusterProminence
24	ADC_original_glcm_ClusterShade
25	ADC_original_glcm_ClusterTendency
26	ADC_original_glcm_Contrast
27	ADC_original_glcm_Correlation
28	ADC_original_glcm_DifferenceAverage
29	ADC_original_glcm_DifferenceEntropy
30	ADC_original_glcm_DifferenceVariance
31	ADC_original_glcm_Id
32	ADC_original_glcm_Idm
33	ADC_original_glcm_Idmn
34	ADC_original_glcm_Idn
35	ADC_original_glcm_Imc1
36	ADC_original_glcm_Imc2
37	ADC_original_glcm_InverseVariance
38	ADC_original_glcm_JointAverage
39	ADC_original_glcm_JointEnergy
40	ADC_original_glcm_JointEntropy
41	ADC_original_glcm_MaximumProbability
42	ADC_original_glcm_SumAverage
43	ADC_original_glcm_SumEntropy
44	ADC_original_glcm_SumSquares
45	ADC_original_glrlm_GrayLevelNonUniformity
46	ADC_original_glrlm_GrayLevelNonUniformityNormalized
47	ADC_original_glrlm_GrayLevelVariance
48	ADC_original_glrlm_HighGrayLevelRunEmphasis
49	ADC_original_glrlm_LongRunEmphasis

50	ADC_original_glrlm_LongRunHighGrayLevelEmphasis
51	ADC_original_glrlm_LongRunLowGrayLevelEmphasis
52	ADC_original_glrlm_LowGrayLevelRunEmphasis
53	ADC_original_glrlm_RunEntropy
54	ADC_original_glrlm_RunLengthNonUniformity
55	ADC_original_glrlm_RunLengthNonUniformityNormalized
56	ADC_original_glrlm_RunPercentage
57	ADC_original_glrlm_RunVariance
58	ADC_original_glrlm_ShortRunEmphasis
59	ADC_original_glrlm_ShortRunHighGrayLevelEmphasis
60	ADC_original_glrlm_ShortRunLowGrayLevelEmphasis
61	ADC_original_glszm_GrayLevelNonUniformity
62	ADC_original_glszm_GrayLevelNonUniformityNormalized
63	ADC_original_glszm_GrayLevelVariance
64	ADC_original_glszm_HighGrayLevelZoneEmphasis
65	ADC_original_glszm_LargeAreaEmphasis
66	ADC_original_glszm_LargeAreaHighGrayLevelEmphasis
67	ADC_original_glszm_LargeAreaLowGrayLevelEmphasis
68	ADC_original_glszm_LowGrayLevelZoneEmphasis
69	ADC_original_glszm_SizeZoneNonUniformity
70	ADC_original_glszm_SizeZoneNonUniformityNormalized
71	ADC_original_glszm_SmallAreaEmphasis
72	ADC_original_glszm_SmallAreaHighGrayLevelEmphasis
73	ADC_original_glszm_SmallAreaLowGrayLevelEmphasis
74	ADC_original_glszm_ZoneEntropy
75	ADC_original_glszm_ZonePercentage
76	ADC_original_glszm_ZoneVariance
77	ADC_original_shape_Elongation
78	ADC_original_shape_Flatness
79	ADC_original_shape_LeastAxis
80	ADC_original_shape_MajorAxis
81	ADC_original_shape_Maximum2DDiameterColumn
82	ADC_original_shape_Maximum2DDiameterRow
83	ADC_original_shape_Maximum2DDiameterSlice
84	ADC_original_shape_Maximum3DDiameter
85	ADC_original_shape_MinorAxis
86	ADC_original_shape_Sphericity
87	ADC_original_shape_SurfaceArea
88	ADC_original_shape_SurfaceVolumeRatio
89	ADC_original_shape_Volume
90	DWI_general_info_VolumeNum
91	DWI_general_info_VoxelNum
92	DWI_original_firstorder_10Percentile
93	DWI_original_firstorder_90Percentile
94	DWI_original_firstorder_Energy
95	DWI_original_firstorder_Entropy
96	DWI_original_firstorder_InterquartileRange
97	DWI_original_firstorder_Kurtosis
98	DWI_original_firstorder_Maximum
99	DWI_original_firstorder_Mean
100	DWI_original_firstorder_MeanAbsoluteDeviation
101	DWI_original_firstorder_Median

102 DWI_original_firstorder_Minimum
103 DWI_original_firstorder_Range
104 DWI_original_firstorder_RobustMeanAbsoluteDeviation
105 DWI_original_firstorder_RootMeanSquared
106 DWI_original_firstorder_Skewness
107 DWI_original_firstorder_TotalEnergy
108 DWI_original_firstorder_Uniformity
109 DWI_original_firstorder_Variance
110 DWI_original_glcm_Autocorrelation
111 DWI_original_glcm_ClusterProminence
112 DWI_original_glcm_ClusterShade
113 DWI_original_glcm_ClusterTendency
114 DWI_original_glcm_Contrast
115 DWI_original_glcm_Correlation
116 DWI_original_glcm_DifferenceAverage
117 DWI_original_glcm_DifferenceEntropy
118 DWI_original_glcm_DifferenceVariance
119 DWI_original_glcm_Id
120 DWI_original_glcm_Idm
121 DWI_original_glcm_Idmn
122 DWI_original_glcm_Idn
123 DWI_original_glcm_Imc1
124 DWI_original_glcm_Imc2
125 DWI_original_glcm_InverseVariance
126 DWI_original_glcm_JointAverage
127 DWI_original_glcm_JointEnergy
128 DWI_original_glcm_JointEntropy
129 DWI_original_glcm_MaximumProbability
130 DWI_original_glcm_SumAverage
131 DWI_original_glcm_SumEntropy
132 DWI_original_glcm_SumSquares
133 DWI_original_glrlm_GrayLevelNonUniformity
134 DWI_original_glrlm_GrayLevelNonUniformityNormalized
135 DWI_original_glrlm_GrayLevelVariance
136 DWI_original_glrlm_HighGrayLevelRunEmphasis
137 DWI_original_glrlm_LongRunEmphasis
138 DWI_original_glrlm_LongRunHighGrayLevelEmphasis
139 DWI_original_glrlm_LongRunLowGrayLevelEmphasis
140 DWI_original_glrlm_LowGrayLevelRunEmphasis
141 DWI_original_glrlm_RunEntropy
142 DWI_original_glrlm_RunLengthNonUniformity
143 DWI_original_glrlm_RunLengthNonUniformityNormalized
144 DWI_original_glrlm_RunPercentage
145 DWI_original_glrlm_RunVariance
146 DWI_original_glrlm_ShortRunEmphasis
147 DWI_original_glrlm_ShortRunHighGrayLevelEmphasis
148 DWI_original_glrlm_ShortRunLowGrayLevelEmphasis
149 DWI_original_glszm_GrayLevelNonUniformity
150 DWI_original_glszm_GrayLevelNonUniformityNormalized
151 DWI_original_glszm_GrayLevelVariance
152 DWI_original_glszm_HighGrayLevelZoneEmphasis
153 DWI_original_glszm_LargeAreaEmphasis

154 DWI_original_glszm_LargeAreaHighGrayLevelEmphasis
155 DWI_original_glszm_LargeAreaLowGrayLevelEmphasis
156 DWI_original_glszm_LowGrayLevelZoneEmphasis
157 DWI_original_glszm_SizeZoneNonUniformity
158 DWI_original_glszm_SizeZoneNonUniformityNormalized
159 DWI_original_glszm_SmallAreaEmphasis
160 DWI_original_glszm_SmallAreaHighGrayLevelEmphasis
161 DWI_original_glszm_SmallAreaLowGrayLevelEmphasis
162 DWI_original_glszm_ZoneEntropy
163 DWI_original_glszm_ZonePercentage
164 DWI_original_glszm_ZoneVariance
165 DWI_original_shape_Elongation
166 DWI_original_shape_Flatness
167 DWI_original_shape_LeastAxis
168 DWI_original_shape_MajorAxis
169 DWI_original_shape_Maximum2DDiameterColumn
170 DWI_original_shape_Maximum2DDiameterRow
171 DWI_original_shape_Maximum2DDiameterSlice
172 DWI_original_shape_Maximum3DDiameter
173 DWI_original_shape_MinorAxis
174 DWI_original_shape_Sphericity
175 DWI_original_shape_SurfaceArea
176 DWI_original_shape_SurfaceVolumeRatio
177 DWI_original_shape_Volume
178 T2_general_info_VolumeNum
179 T2_general_info_VoxelNum
180 T2_original_firstorder_10Percentile
181 T2_original_firstorder_90Percentile
182 T2_original_firstorder_Energy
183 T2_original_firstorder_Entropy
184 T2_original_firstorder_InterquartileRange
185 T2_original_firstorder_Kurtosis
186 T2_original_firstorder_Maximum
187 T2_original_firstorder_Mean
188 T2_original_firstorder_MeanAbsoluteDeviation
189 T2_original_firstorder_Median
190 T2_original_firstorder_Minimum
191 T2_original_firstorder_Range
192 T2_original_firstorder_RobustMeanAbsoluteDeviation
193 T2_original_firstorder_RootMeanSquared
194 T2_original_firstorder_Skewness
195 T2_original_firstorder_TotalEnergy
196 T2_original_firstorder_Uniformity
197 T2_original_firstorder_Variance
198 T2_original_glcm_Autocorrelation
199 T2_original_glcm_ClusterProminence
200 T2_original_glcm_ClusterShade
201 T2_original_glcm_ClusterTendency
202 T2_original_glcm_Contrast
203 T2_original_glcm_Correlation
204 T2_original_glcm_DifferenceAverage
205 T2_original_glcm_DifferenceEntropy

206 T2_original_glcm_DifferenceVariance
207 T2_original_glcm_Id
208 T2_original_glcm_Idm
209 T2_original_glcm_Idmn
210 T2_original_glcm_Idn
211 T2_original_glcm_Imc1
212 T2_original_glcm_Imc2
213 T2_original_glcm_InverseVariance
214 T2_original_glcm_JointAverage
215 T2_original_glcm_JointEnergy
216 T2_original_glcm_JointEntropy
217 T2_original_glcm_MaximumProbability
218 T2_original_glcm_SumAverage
219 T2_original_glcm_SumEntropy
220 T2_original_glcm_SumSquares
221 T2_original_glrlm_GrayLevelNonUniformity
222 T2_original_glrlm_GrayLevelNonUniformityNormalized
223 T2_original_glrlm_GrayLevelVariance
224 T2_original_glrlm_HighGrayLevelRunEmphasis
225 T2_original_glrlm_LongRunEmphasis
226 T2_original_glrlm_LongRunHighGrayLevelEmphasis
227 T2_original_glrlm_LongRunLowGrayLevelEmphasis
228 T2_original_glrlm_LowGrayLevelRunEmphasis
229 T2_original_glrlm_RunEntropy
230 T2_original_glrlm_RunLengthNonUniformity
231 T2_original_glrlm_RunLengthNonUniformityNormalized
232 T2_original_glrlm_RunPercentage
233 T2_original_glrlm_RunVariance
234 T2_original_glrlm_ShortRunEmphasis
235 T2_original_glrlm_ShortRunHighGrayLevelEmphasis
236 T2_original_glrlm_ShortRunLowGrayLevelEmphasis
237 T2_original_glszm_GrayLevelNonUniformity
238 T2_original_glszm_GrayLevelNonUniformityNormalized
239 T2_original_glszm_GrayLevelVariance
240 T2_original_glszm_HighGrayLevelZoneEmphasis
241 T2_original_glszm_LargeAreaEmphasis
242 T2_original_glszm_LargeAreaHighGrayLevelEmphasis
243 T2_original_glszm_LargeAreaLowGrayLevelEmphasis
244 T2_original_glszm_LowGrayLevelZoneEmphasis
245 T2_original_glszm_SizeZoneNonUniformity
246 T2_original_glszm_SizeZoneNonUniformityNormalized
247 T2_original_glszm_SmallAreaEmphasis
248 T2_original_glszm_SmallAreaHighGrayLevelEmphasis
249 T2_original_glszm_SmallAreaLowGrayLevelEmphasis
250 T2_original_glszm_ZoneEntropy
251 T2_original_glszm_ZonePercentage
252 T2_original_glszm_ZoneVariance
253 T2_original_shape_Elongation
254 T2_original_shape_Flatness
255 T2_original_shape_LeastAxis
256 T2_original_shape_MajorAxis
257 T2_original_shape_Maximum2DDiameterColumn

258	T2_original_shape_Maximum2DDiameterRow
259	T2_original_shape_Maximum2DDiameterSlice
260	T2_original_shape_Maximum3DDiameter
261	T2_original_shape_MinorAxis
262	T2_original_shape_Sphericity
263	T2_original_shape_SurfaceArea
264	T2_original_shape_SurfaceVolumeRatio
265	T2_original_shape_Volume

Supplementary Table S2. Showing the feature index with the feature name in Dataset 2.

index	label
1	ADCm.3.glcm.contrast.1.mean.
2	ADCm.3.glcm.contrast.1.range.
3	ADCm.3.glcm.contrast.2.mean.
4	ADCm.3.glcm.contrast.2.range.
5	ADCm.3.glcm.contrast.3.mean.
6	ADCm.3.glcm.contrast.3.range.
7	ADCm.3.glcm.dissimilarity.1.mean.
8	ADCm.3.glcm.dissimilarity.1.range.
9	ADCm.3.glcm.dissimilarity.2.mean.
10	ADCm.3.glcm.dissimilarity.2.range.
11	ADCm.3.glcm.dissimilarity.3.mean.
12	ADCm.3.glcm.dissimilarity.3.range.
13	ADCm.3.glcm.homogeneity.1.mean.
14	ADCm.3.glcm.homogeneity.1.range.
15	ADCm.3.glcm.homogeneity.2.mean.
16	ADCm.3.glcm.homogeneity.2.range.
17	ADCm.3.glcm.homogeneity.3.mean.
18	ADCm.3.glcm.homogeneity.3.range.
19	ADCm.3.glcm.energy.1.mean.
20	ADCm.3.glcm.energy.1.range.
21	ADCm.3.glcm.energy.2.mean.
22	ADCm.3.glcm.energy.2.range.
23	ADCm.3.glcm.energy.3.mean.
24	ADCm.3.glcm.energy.3.range.
25	ADCm.3.glcm.correlation.1.mean.
26	ADCm.3.glcm.correlation.1.range.
27	ADCm.3.glcm.correlation.2.mean.
28	ADCm.3.glcm.correlation.2.range.

29	ADCm.3.glcm.correlation.3.mean.
30	ADCm.3.glcm.correlation.3.range.
31	ADCm.3.glcm.ASM.1.mean.
32	ADCm.3.glcm.ASM.1.range.
33	ADCm.3.glcm.ASM.2.mean.
34	ADCm.3.glcm.ASM.2.range.
35	ADCm.3.glcm.ASM.3.mean.
36	ADCm.3.glcm.ASM.3.range.
37	ADCm.5.glcm.contrast.1.mean.
38	ADCm.5.glcm.contrast.1.range.
39	ADCm.5.glcm.contrast.2.mean.
40	ADCm.5.glcm.contrast.2.range.
41	ADCm.5.glcm.contrast.3.mean.
42	ADCm.5.glcm.contrast.3.range.
43	ADCm.5.glcm.contrast.4.mean.
44	ADCm.5.glcm.contrast.4.range.
45	ADCm.5.glcm.dissimilarity.1.mean.
46	ADCm.5.glcm.dissimilarity.1.range.
47	ADCm.5.glcm.dissimilarity.2.mean.
48	ADCm.5.glcm.dissimilarity.2.range.
49	ADCm.5.glcm.dissimilarity.3.mean.
50	ADCm.5.glcm.dissimilarity.3.range.
51	ADCm.5.glcm.dissimilarity.4.mean.
52	ADCm.5.glcm.dissimilarity.4.range.
53	ADCm.5.glcm.homogeneity.1.mean.
54	ADCm.5.glcm.homogeneity.1.range.
55	ADCm.5.glcm.homogeneity.2.mean.
56	ADCm.5.glcm.homogeneity.2.range.
57	ADCm.5.glcm.homogeneity.3.mean.
58	ADCm.5.glcm.homogeneity.3.range.
59	ADCm.5.glcm.homogeneity.4.mean.
60	ADCm.5.glcm.homogeneity.4.range.
61	ADCm.5.glcm.energy.1.mean.
62	ADCm.5.glcm.energy.1.range.
63	ADCm.5.glcm.energy.2.mean.
64	ADCm.5.glcm.energy.2.range.
65	ADCm.5.glcm.energy.3.mean.

66	ADCm.5.glcm.energy.3.range.
67	ADCm.5.glcm.energy.4.mean.
68	ADCm.5.glcm.energy.4.range.
69	ADCm.5.glcm.correlation.1.mean.
70	ADCm.5.glcm.correlation.1.range.
71	ADCm.5.glcm.correlation.2.mean.
72	ADCm.5.glcm.correlation.2.range.
73	ADCm.5.glcm.correlation.3.mean.
74	ADCm.5.glcm.correlation.3.range.
75	ADCm.5.glcm.correlation.4.mean.
76	ADCm.5.glcm.correlation.4.range.
77	ADCm.5.glcm.ASM.1.mean.
78	ADCm.5.glcm.ASM.1.range.
79	ADCm.5.glcm.ASM.2.mean.
80	ADCm.5.glcm.ASM.2.range.
81	ADCm.5.glcm.ASM.3.mean.
82	ADCm.5.glcm.ASM.3.range.
83	ADCm.5.glcm.ASM.4.mean.
84	ADCm.5.glcm.ASM.4.range.
85	ADCm.7.glcm.contrast.1.mean.
86	ADCm.7.glcm.contrast.1.range.
87	ADCm.7.glcm.contrast.2.mean.
88	ADCm.7.glcm.contrast.2.range.
89	ADCm.7.glcm.contrast.3.mean.
90	ADCm.7.glcm.contrast.3.range.
91	ADCm.7.glcm.contrast.4.mean.
92	ADCm.7.glcm.contrast.4.range.
93	ADCm.7.glcm.dissimilarity.1.mean.
94	ADCm.7.glcm.dissimilarity.1.range.
95	ADCm.7.glcm.dissimilarity.2.mean.
96	ADCm.7.glcm.dissimilarity.2.range.
97	ADCm.7.glcm.dissimilarity.3.mean.
98	ADCm.7.glcm.dissimilarity.3.range.
99	ADCm.7.glcm.dissimilarity.4.mean.
100	ADCm.7.glcm.dissimilarity.4.range.
101	ADCm.7.glcm.homogeneity.1.mean.
102	ADCm.7.glcm.homogeneity.1.range.

103	ADCm.7.glcm.homogeneity.2.mean.
104	ADCm.7.glcm.homogeneity.2.range.
105	ADCm.7.glcm.homogeneity.3.mean.
106	ADCm.7.glcm.homogeneity.3.range.
107	ADCm.7.glcm.homogeneity.4.mean.
108	ADCm.7.glcm.homogeneity.4.range.
109	ADCm.7.glcm.energy.1.mean.
110	ADCm.7.glcm.energy.1.range.
111	ADCm.7.glcm.energy.2.mean.
112	ADCm.7.glcm.energy.2.range.
113	ADCm.7.glcm.energy.3.mean.
114	ADCm.7.glcm.energy.3.range.
115	ADCm.7.glcm.energy.4.mean.
116	ADCm.7.glcm.energy.4.range.
117	ADCm.7.glcm.correlation.1.mean.
118	ADCm.7.glcm.correlation.1.range.
119	ADCm.7.glcm.correlation.2.mean.
120	ADCm.7.glcm.correlation.2.range.
121	ADCm.7.glcm.correlation.3.mean.
122	ADCm.7.glcm.correlation.3.range.
123	ADCm.7.glcm.correlation.4.mean.
124	ADCm.7.glcm.correlation.4.range.
125	ADCm.7.glcm.ASM.1.mean.
126	ADCm.7.glcm.ASM.1.range.
127	ADCm.7.glcm.ASM.2.mean.
128	ADCm.7.glcm.ASM.2.range.
129	ADCm.7.glcm.ASM.3.mean.
130	ADCm.7.glcm.ASM.3.range.
131	ADCm.7.glcm.ASM.4.mean.
132	ADCm.7.glcm.ASM.4.range.
133	ADCm.9.glcm.contrast.1.mean.
134	ADCm.9.glcm.contrast.1.range.
135	ADCm.9.glcm.contrast.2.mean.
136	ADCm.9.glcm.contrast.2.range.
137	ADCm.9.glcm.contrast.3.mean.
138	ADCm.9.glcm.contrast.3.range.
139	ADCm.9.glcm.contrast.4.mean.

140	ADCm.9.glcm.contrast.4.range.
141	ADCm.9.glcm.dissimilarity.1.mean.
142	ADCm.9.glcm.dissimilarity.1.range.
143	ADCm.9.glcm.dissimilarity.2.mean.
144	ADCm.9.glcm.dissimilarity.2.range.
145	ADCm.9.glcm.dissimilarity.3.mean.
146	ADCm.9.glcm.dissimilarity.3.range.
147	ADCm.9.glcm.dissimilarity.4.mean.
148	ADCm.9.glcm.dissimilarity.4.range.
149	ADCm.9.glcm.homogeneity.1.mean.
150	ADCm.9.glcm.homogeneity.1.range.
151	ADCm.9.glcm.homogeneity.2.mean.
152	ADCm.9.glcm.homogeneity.2.range.
153	ADCm.9.glcm.homogeneity.3.mean.
154	ADCm.9.glcm.homogeneity.3.range.
155	ADCm.9.glcm.homogeneity.4.mean.
156	ADCm.9.glcm.homogeneity.4.range.
157	ADCm.9.glcm.energy.1.mean.
158	ADCm.9.glcm.energy.1.range.
159	ADCm.9.glcm.energy.2.mean.
160	ADCm.9.glcm.energy.2.range.
161	ADCm.9.glcm.energy.3.mean.
162	ADCm.9.glcm.energy.3.range.
163	ADCm.9.glcm.energy.4.mean.
164	ADCm.9.glcm.energy.4.range.
165	ADCm.9.glcm.correlation.1.mean.
166	ADCm.9.glcm.correlation.1.range.
167	ADCm.9.glcm.correlation.2.mean.
168	ADCm.9.glcm.correlation.2.range.
169	ADCm.9.glcm.correlation.3.mean.
170	ADCm.9.glcm.correlation.3.range.
171	ADCm.9.glcm.correlation.4.mean.
172	ADCm.9.glcm.correlation.4.range.
173	ADCm.9.glcm.ASM.1.mean.
174	ADCm.9.glcm.ASM.1.range.
175	ADCm.9.glcm.ASM.2.mean.
176	ADCm.9.glcm.ASM.2.range.

177	ADCm.9.glcm.ASM.3.mean.
178	ADCm.9.glcm.ASM.3.range.
179	ADCm.9.glcm.ASM.4.mean.
180	ADCm.9.glcm.ASM.4.range.
181	ADCm.11.glcm.contrast.1.mean.
182	ADCm.11.glcm.contrast.1.range.
183	ADCm.11.glcm.contrast.2.mean.
184	ADCm.11.glcm.contrast.2.range.
185	ADCm.11.glcm.contrast.3.mean.
186	ADCm.11.glcm.contrast.3.range.
187	ADCm.11.glcm.contrast.4.mean.
188	ADCm.11.glcm.contrast.4.range.
189	ADCm.11.glcm.dissimilarity.1.mean.
190	ADCm.11.glcm.dissimilarity.1.range.
191	ADCm.11.glcm.dissimilarity.2.mean.
192	ADCm.11.glcm.dissimilarity.2.range.
193	ADCm.11.glcm.dissimilarity.3.mean.
194	ADCm.11.glcm.dissimilarity.3.range.
195	ADCm.11.glcm.dissimilarity.4.mean.
196	ADCm.11.glcm.dissimilarity.4.range.
197	ADCm.11.glcm.homogeneity.1.mean.
198	ADCm.11.glcm.homogeneity.1.range.
199	ADCm.11.glcm.homogeneity.2.mean.
200	ADCm.11.glcm.homogeneity.2.range.
201	ADCm.11.glcm.homogeneity.3.mean.
202	ADCm.11.glcm.homogeneity.3.range.
203	ADCm.11.glcm.homogeneity.4.mean.
204	ADCm.11.glcm.homogeneity.4.range.
205	ADCm.11.glcm.energy.1.mean.
206	ADCm.11.glcm.energy.1.range.
207	ADCm.11.glcm.energy.2.mean.
208	ADCm.11.glcm.energy.2.range.
209	ADCm.11.glcm.energy.3.mean.
210	ADCm.11.glcm.energy.3.range.
211	ADCm.11.glcm.energy.4.mean.
212	ADCm.11.glcm.energy.4.range.
213	ADCm.11.glcm.correlation.1.mean.

214	ADCm.11.glcm.correlation.1.range.
215	ADCm.11.glcm.correlation.2.mean.
216	ADCm.11.glcm.correlation.2.range.
217	ADCm.11.glcm.correlation.3.mean.
218	ADCm.11.glcm.correlation.3.range.
219	ADCm.11.glcm.correlation.4.mean.
220	ADCm.11.glcm.correlation.4.range.
221	ADCm.11.glcm.ASM.1.mean.
222	ADCm.11.glcm.ASM.1.range.
223	ADCm.11.glcm.ASM.2.mean.
224	ADCm.11.glcm.ASM.2.range.
225	ADCm.11.glcm.ASM.3.mean.
226	ADCm.11.glcm.ASM.3.range.
227	ADCm.11.glcm.ASM.4.mean.
228	ADCm.11.glcm.ASM.4.range.
229	ADCm.13.glcm.contrast.1.mean.
230	ADCm.13.glcm.contrast.1.range.
231	ADCm.13.glcm.contrast.2.mean.
232	ADCm.13.glcm.contrast.2.range.
233	ADCm.13.glcm.contrast.3.mean.
234	ADCm.13.glcm.contrast.3.range.
235	ADCm.13.glcm.contrast.4.mean.
236	ADCm.13.glcm.contrast.4.range.
237	ADCm.13.glcm.dissimilarity.1.mean.
238	ADCm.13.glcm.dissimilarity.1.range.
239	ADCm.13.glcm.dissimilarity.2.mean.
240	ADCm.13.glcm.dissimilarity.2.range.
241	ADCm.13.glcm.dissimilarity.3.mean.
242	ADCm.13.glcm.dissimilarity.3.range.
243	ADCm.13.glcm.dissimilarity.4.mean.
244	ADCm.13.glcm.dissimilarity.4.range.
245	ADCm.13.glcm.homogeneity.1.mean.
246	ADCm.13.glcm.homogeneity.1.range.
247	ADCm.13.glcm.homogeneity.2.mean.
248	ADCm.13.glcm.homogeneity.2.range.
249	ADCm.13.glcm.homogeneity.3.mean.
250	ADCm.13.glcm.homogeneity.3.range.

251	ADCm.13.glcm.homogeneity.4.mean.
252	ADCm.13.glcm.homogeneity.4.range.
253	ADCm.13.glcm.energy.1.mean.
254	ADCm.13.glcm.energy.1.range.
255	ADCm.13.glcm.energy.2.mean.
256	ADCm.13.glcm.energy.2.range.
257	ADCm.13.glcm.energy.3.mean.
258	ADCm.13.glcm.energy.3.range.
259	ADCm.13.glcm.energy.4.mean.
260	ADCm.13.glcm.energy.4.range.
261	ADCm.13.glcm.correlation.1.mean.
262	ADCm.13.glcm.correlation.1.range.
263	ADCm.13.glcm.correlation.2.mean.
264	ADCm.13.glcm.correlation.2.range.
265	ADCm.13.glcm.correlation.3.mean.
266	ADCm.13.glcm.correlation.3.range.
267	ADCm.13.glcm.correlation.4.mean.
268	ADCm.13.glcm.correlation.4.range.
269	ADCm.13.glcm.ASM.1.mean.
270	ADCm.13.glcm.ASM.1.range.
271	ADCm.13.glcm.ASM.2.mean.
272	ADCm.13.glcm.ASM.2.range.
273	ADCm.13.glcm.ASM.3.mean.
274	ADCm.13.glcm.ASM.3.range.
275	ADCm.13.glcm.ASM.4.mean.
276	ADCm.13.glcm.ASM.4.range.
277	ADCm.15.glcm.contrast.1.mean.
278	ADCm.15.glcm.contrast.1.range.
279	ADCm.15.glcm.contrast.2.mean.
280	ADCm.15.glcm.contrast.2.range.
281	ADCm.15.glcm.contrast.3.mean.
282	ADCm.15.glcm.contrast.3.range.
283	ADCm.15.glcm.contrast.4.mean.
284	ADCm.15.glcm.contrast.4.range.
285	ADCm.15.glcm.dissimilarity.1.mean.
286	ADCm.15.glcm.dissimilarity.1.range.
287	ADCm.15.glcm.dissimilarity.2.mean.

288	ADCm.15.glcm.dissimilarity.2.range.
289	ADCm.15.glcm.dissimilarity.3.mean.
290	ADCm.15.glcm.dissimilarity.3.range.
291	ADCm.15.glcm.dissimilarity.4.mean.
292	ADCm.15.glcm.dissimilarity.4.range.
293	ADCm.15.glcm.homogeneity.1.mean.
294	ADCm.15.glcm.homogeneity.1.range.
295	ADCm.15.glcm.homogeneity.2.mean.
296	ADCm.15.glcm.homogeneity.2.range.
297	ADCm.15.glcm.homogeneity.3.mean.
298	ADCm.15.glcm.homogeneity.3.range.
299	ADCm.15.glcm.homogeneity.4.mean.
300	ADCm.15.glcm.homogeneity.4.range.
301	ADCm.15.glcm.energy.1.mean.
302	ADCm.15.glcm.energy.1.range.
303	ADCm.15.glcm.energy.2.mean.
304	ADCm.15.glcm.energy.2.range.
305	ADCm.15.glcm.energy.3.mean.
306	ADCm.15.glcm.energy.3.range.
307	ADCm.15.glcm.energy.4.mean.
308	ADCm.15.glcm.energy.4.range.
309	ADCm.15.glcm.correlation.1.mean.
310	ADCm.15.glcm.correlation.1.range.
311	ADCm.15.glcm.correlation.2.mean.
312	ADCm.15.glcm.correlation.2.range.
313	ADCm.15.glcm.correlation.3.mean.
314	ADCm.15.glcm.correlation.3.range.
315	ADCm.15.glcm.correlation.4.mean.
316	ADCm.15.glcm.correlation.4.range.
317	ADCm.15.glcm.ASM.1.mean.
318	ADCm.15.glcm.ASM.1.range.
319	ADCm.15.glcm.ASM.2.mean.
320	ADCm.15.glcm.ASM.2.range.
321	ADCm.15.glcm.ASM.3.mean.
322	ADCm.15.glcm.ASM.3.range.
323	ADCm.15.glcm.ASM.4.mean.
324	ADCm.15.glcm.ASM.4.range.

325	ADCm.mbb.glcm.contrast.1.mean.
326	ADCm.mbb.glcm.contrast.1.range.
327	ADCm.mbb.glcm.contrast.2.mean.
328	ADCm.mbb.glcm.contrast.2.range.
329	ADCm.mbb.glcm.contrast.3.mean.
330	ADCm.mbb.glcm.contrast.3.range.
331	ADCm.mbb.glcm.contrast.4.mean.
332	ADCm.mbb.glcm.contrast.4.range.
333	ADCm.mbb.glcm.dissimilarity.1.mean.
334	ADCm.mbb.glcm.dissimilarity.1.range.
335	ADCm.mbb.glcm.dissimilarity.2.mean.
336	ADCm.mbb.glcm.dissimilarity.2.range.
337	ADCm.mbb.glcm.dissimilarity.3.mean.
338	ADCm.mbb.glcm.dissimilarity.3.range.
339	ADCm.mbb.glcm.dissimilarity.4.mean.
340	ADCm.mbb.glcm.dissimilarity.4.range.
341	ADCm.mbb.glcm.homogeneity.1.mean.
342	ADCm.mbb.glcm.homogeneity.1.range.
343	ADCm.mbb.glcm.homogeneity.2.mean.
344	ADCm.mbb.glcm.homogeneity.2.range.
345	ADCm.mbb.glcm.homogeneity.3.mean.
346	ADCm.mbb.glcm.homogeneity.3.range.
347	ADCm.mbb.glcm.homogeneity.4.mean.
348	ADCm.mbb.glcm.homogeneity.4.range.
349	ADCm.mbb.glcm.energy.1.mean.
350	ADCm.mbb.glcm.energy.1.range.

351	ADCm.mbb.glcm.energy.2.mean.
352	ADCm.mbb.glcm.energy.2.range.
353	ADCm.mbb.glcm.energy.3.mean.
354	ADCm.mbb.glcm.energy.3.range.
355	ADCm.mbb.glcm.energy.4.mean.
356	ADCm.mbb.glcm.energy.4.range.
357	ADCm.mbb.glcm.correlation.1.mean.
358	ADCm.mbb.glcm.correlation.1.range.
359	ADCm.mbb.glcm.correlation.2.mean.
360	ADCm.mbb.glcm.correlation.2.range.
361	ADCm.mbb.glcm.correlation.3.mean.
362	ADCm.mbb.glcm.correlation.3.range.
363	ADCm.mbb.glcm.correlation.4.mean.
364	ADCm.mbb.glcm.correlation.4.range.
365	ADCm.mbb.glcm.ASM.1.mean.
366	ADCm.mbb.glcm.ASM.1.range.
367	ADCm.mbb.glcm.ASM.2.mean.
368	ADCm.mbb.glcm.ASM.2.range.
369	ADCm.mbb.glcm.ASM.3.mean.
370	ADCm.mbb.glcm.ASM.3.range.
371	ADCm.mbb.glcm.ASM.4.mean.
372	ADCm.mbb.glcm.ASM.4.range.
373	ADCm.3.lbp.1.0.
374	ADCm.3.lbp.1.1.
375	ADCm.3.lbp.1.2.
376	ADCm.3.lbp.1.3.
377	ADCm.3.lbp.1.4.
378	ADCm.3.lbp.1.5.
379	ADCm.3.lbp.1.6.
380	ADCm.3.lbp.1.7.
381	ADCm.3.lbp.1.8.
382	ADCm.3.lbp.1.9.
383	ADCm.5.lbp.2.0.
384	ADCm.5.lbp.2.1.
385	ADCm.5.lbp.2.2.
386	ADCm.5.lbp.2.3.
387	ADCm.5.lbp.2.4.

388	ADCm.5.lbp.2.5.
389	ADCm.5.lbp.2.6.
390	ADCm.5.lbp.2.7.
391	ADCm.5.lbp.2.8.
392	ADCm.5.lbp.2.9.
393	ADCm.7.lbp.3.0.
394	ADCm.7.lbp.3.1.
395	ADCm.7.lbp.3.2.
396	ADCm.7.lbp.3.3.
397	ADCm.7.lbp.3.4.
398	ADCm.7.lbp.3.5.
399	ADCm.7.lbp.3.6.
400	ADCm.7.lbp.3.7.
401	ADCm.7.lbp.3.8.
402	ADCm.7.lbp.3.9.
403	ADCm.9.lbp.4.0.
404	ADCm.9.lbp.4.1.
405	ADCm.9.lbp.4.2.
406	ADCm.9.lbp.4.3.
407	ADCm.9.lbp.4.4.
408	ADCm.9.lbp.4.5.
409	ADCm.9.lbp.4.6.
410	ADCm.9.lbp.4.7.
411	ADCm.9.lbp.4.8.
412	ADCm.9.lbp.4.9.
413	ADCm.11.lbp.5.0.
414	ADCm.11.lbp.5.1.
415	ADCm.11.lbp.5.2.
416	ADCm.11.lbp.5.3.
417	ADCm.11.lbp.5.4.
418	ADCm.11.lbp.5.5.
419	ADCm.11.lbp.5.6.
420	ADCm.11.lbp.5.7.
421	ADCm.11.lbp.5.8.
422	ADCm.11.lbp.5.9.
423	ADCm.13.lbp.6.0.
424	ADCm.13.lbp.6.1.

425	ADCm.13.lbp.6.2.
426	ADCm.13.lbp.6.3.
427	ADCm.13.lbp.6.4.
428	ADCm.13.lbp.6.5.
429	ADCm.13.lbp.6.6.
430	ADCm.13.lbp.6.7.
431	ADCm.13.lbp.6.8.
432	ADCm.13.lbp.6.9.
433	ADCm.15.lbp.7.0.
434	ADCm.15.lbp.7.1.
435	ADCm.15.lbp.7.2.
436	ADCm.15.lbp.7.3.
437	ADCm.15.lbp.7.4.
438	ADCm.15.lbp.7.5.
439	ADCm.15.lbp.7.6.
440	ADCm.15.lbp.7.7.
441	ADCm.15.lbp.7.8.
442	ADCm.15.lbp.7.9.
443	ADCm.3.hog
444	ADCm.5.hog
445	ADCm.7.hog
446	ADCm.9.hog
447	ADCm.11.hog
448	ADCm.13.hog
449	ADCm.15.hog
450	ADCm.3.gabor.1.0.1.mean.
451	ADCm.3.gabor.1.0.1.var.
452	ADCm.3.gabor.1.0.1.absmean.
453	ADCm.3.gabor.1.0.1.mag.
454	ADCm.3.gabor.1.0.2.mean.
455	ADCm.3.gabor.1.0.2.var.
456	ADCm.3.gabor.1.0.2.absmean.
457	ADCm.3.gabor.1.0.2.mag.
458	ADCm.3.gabor.1.0.3.mean.
459	ADCm.3.gabor.1.0.3.var.
460	ADCm.3.gabor.1.0.3.absmean.
461	ADCm.3.gabor.1.0.3.mag.

462	ADCm.3.gabor.1.0.4.mean.
463	ADCm.3.gabor.1.0.4.var.
464	ADCm.3.gabor.1.0.4.absmean.
465	ADCm.3.gabor.1.0.4.mag.
466	ADCm.3.gabor.1.0.5.mean.
467	ADCm.3.gabor.1.0.5.var.
468	ADCm.3.gabor.1.0.5.absmean.
469	ADCm.3.gabor.1.0.5.mag.
470	ADCm.3.gabor.2.0.1.mean.
471	ADCm.3.gabor.2.0.1.var.
472	ADCm.3.gabor.2.0.1.absmean.
473	ADCm.3.gabor.2.0.1.mag.
474	ADCm.3.gabor.2.0.2.mean.
475	ADCm.3.gabor.2.0.2.var.
476	ADCm.3.gabor.2.0.2.absmean.
477	ADCm.3.gabor.2.0.2.mag.
478	ADCm.3.gabor.2.0.3.mean.
479	ADCm.3.gabor.2.0.3.var.
480	ADCm.3.gabor.2.0.3.absmean.
481	ADCm.3.gabor.2.0.3.mag.
482	ADCm.3.gabor.2.0.4.mean.
483	ADCm.3.gabor.2.0.4.var.
484	ADCm.3.gabor.2.0.4.absmean.
485	ADCm.3.gabor.2.0.4.mag.
486	ADCm.3.gabor.2.0.5.mean.
487	ADCm.3.gabor.2.0.5.var.
488	ADCm.3.gabor.2.0.5.absmean.
489	ADCm.3.gabor.2.0.5.mag.
490	ADCm.3.gabor.3.0.1.mean.
491	ADCm.3.gabor.3.0.1.var.
492	ADCm.3.gabor.3.0.1.absmean.
493	ADCm.3.gabor.3.0.1.mag.
494	ADCm.3.gabor.3.0.2.mean.
495	ADCm.3.gabor.3.0.2.var.
496	ADCm.3.gabor.3.0.2.absmean.
497	ADCm.3.gabor.3.0.2.mag.
498	ADCm.3.gabor.3.0.3.mean.

499	ADCm.3.gabor.3.0.3.var.
500	ADCm.3.gabor.3.0.3.absmean.
501	ADCm.3.gabor.3.0.3.mag.
502	ADCm.3.gabor.3.0.4.mean.
503	ADCm.3.gabor.3.0.4.var.
504	ADCm.3.gabor.3.0.4.absmean.
505	ADCm.3.gabor.3.0.4.mag.
506	ADCm.3.gabor.3.0.5.mean.
507	ADCm.3.gabor.3.0.5.var.
508	ADCm.3.gabor.3.0.5.absmean.
509	ADCm.3.gabor.3.0.5.mag.
510	ADCm.5.gabor.1.0.1.mean.
511	ADCm.5.gabor.1.0.1.var.
512	ADCm.5.gabor.1.0.1.absmean.
513	ADCm.5.gabor.1.0.1.mag.
514	ADCm.5.gabor.1.0.2.mean.
515	ADCm.5.gabor.1.0.2.var.
516	ADCm.5.gabor.1.0.2.absmean.
517	ADCm.5.gabor.1.0.2.mag.
518	ADCm.5.gabor.1.0.3.mean.
519	ADCm.5.gabor.1.0.3.var.
520	ADCm.5.gabor.1.0.3.absmean.
521	ADCm.5.gabor.1.0.3.mag.
522	ADCm.5.gabor.1.0.4.mean.
523	ADCm.5.gabor.1.0.4.var.
524	ADCm.5.gabor.1.0.4.absmean.
525	ADCm.5.gabor.1.0.4.mag.
526	ADCm.5.gabor.1.0.5.mean.
527	ADCm.5.gabor.1.0.5.var.
528	ADCm.5.gabor.1.0.5.absmean.
529	ADCm.5.gabor.1.0.5.mag.
530	ADCm.5.gabor.2.0.1.mean.
531	ADCm.5.gabor.2.0.1.var.
532	ADCm.5.gabor.2.0.1.absmean.
533	ADCm.5.gabor.2.0.1.mag.
534	ADCm.5.gabor.2.0.2.mean.
535	ADCm.5.gabor.2.0.2.var.

536	ADCm.5.gabor.2.0.2.absmean.
537	ADCm.5.gabor.2.0.2.mag.
538	ADCm.5.gabor.2.0.3.mean.
539	ADCm.5.gabor.2.0.3.var.
540	ADCm.5.gabor.2.0.3.absmean.
541	ADCm.5.gabor.2.0.3.mag.
542	ADCm.5.gabor.2.0.4.mean.
543	ADCm.5.gabor.2.0.4.var.
544	ADCm.5.gabor.2.0.4.absmean.
545	ADCm.5.gabor.2.0.4.mag.
546	ADCm.5.gabor.2.0.5.mean.
547	ADCm.5.gabor.2.0.5.var.
548	ADCm.5.gabor.2.0.5.absmean.
549	ADCm.5.gabor.2.0.5.mag.
550	ADCm.5.gabor.3.0.1.mean.
551	ADCm.5.gabor.3.0.1.var.
552	ADCm.5.gabor.3.0.1.absmean.
553	ADCm.5.gabor.3.0.1.mag.
554	ADCm.5.gabor.3.0.2.mean.
555	ADCm.5.gabor.3.0.2.var.
556	ADCm.5.gabor.3.0.2.absmean.
557	ADCm.5.gabor.3.0.2.mag.
558	ADCm.5.gabor.3.0.3.mean.
559	ADCm.5.gabor.3.0.3.var.
560	ADCm.5.gabor.3.0.3.absmean.
561	ADCm.5.gabor.3.0.3.mag.
562	ADCm.5.gabor.3.0.4.mean.
563	ADCm.5.gabor.3.0.4.var.
564	ADCm.5.gabor.3.0.4.absmean.
565	ADCm.5.gabor.3.0.4.mag.
566	ADCm.5.gabor.3.0.5.mean.
567	ADCm.5.gabor.3.0.5.var.
568	ADCm.5.gabor.3.0.5.absmean.
569	ADCm.5.gabor.3.0.5.mag.
570	ADCm.7.gabor.1.0.1.mean.
571	ADCm.7.gabor.1.0.1.var.
572	ADCm.7.gabor.1.0.1.absmean.

573	ADCm.7.gabor.1.0.1.mag.
574	ADCm.7.gabor.1.0.2.mean.
575	ADCm.7.gabor.1.0.2.var.
576	ADCm.7.gabor.1.0.2.absmean.
577	ADCm.7.gabor.1.0.2.mag.
578	ADCm.7.gabor.1.0.3.mean.
579	ADCm.7.gabor.1.0.3.var.
580	ADCm.7.gabor.1.0.3.absmean.
581	ADCm.7.gabor.1.0.3.mag.
582	ADCm.7.gabor.1.0.4.mean.
583	ADCm.7.gabor.1.0.4.var.
584	ADCm.7.gabor.1.0.4.absmean.
585	ADCm.7.gabor.1.0.4.mag.
586	ADCm.7.gabor.1.0.5.mean.
587	ADCm.7.gabor.1.0.5.var.
588	ADCm.7.gabor.1.0.5.absmean.
589	ADCm.7.gabor.1.0.5.mag.
590	ADCm.7.gabor.2.0.1.mean.
591	ADCm.7.gabor.2.0.1.var.
592	ADCm.7.gabor.2.0.1.absmean.
593	ADCm.7.gabor.2.0.1.mag.
594	ADCm.7.gabor.2.0.2.mean.
595	ADCm.7.gabor.2.0.2.var.
596	ADCm.7.gabor.2.0.2.absmean.
597	ADCm.7.gabor.2.0.2.mag.
598	ADCm.7.gabor.2.0.3.mean.
599	ADCm.7.gabor.2.0.3.var.
600	ADCm.7.gabor.2.0.3.absmean.
601	ADCm.7.gabor.2.0.3.mag.
602	ADCm.7.gabor.2.0.4.mean.
603	ADCm.7.gabor.2.0.4.var.
604	ADCm.7.gabor.2.0.4.absmean.
605	ADCm.7.gabor.2.0.4.mag.
606	ADCm.7.gabor.2.0.5.mean.
607	ADCm.7.gabor.2.0.5.var.
608	ADCm.7.gabor.2.0.5.absmean.
609	ADCm.7.gabor.2.0.5.mag.

610	ADCm.7.gabor.3.0.1.mean.
611	ADCm.7.gabor.3.0.1.var.
612	ADCm.7.gabor.3.0.1.absmean.
613	ADCm.7.gabor.3.0.1.mag.
614	ADCm.7.gabor.3.0.2.mean.
615	ADCm.7.gabor.3.0.2.var.
616	ADCm.7.gabor.3.0.2.absmean.
617	ADCm.7.gabor.3.0.2.mag.
618	ADCm.7.gabor.3.0.3.mean.
619	ADCm.7.gabor.3.0.3.var.
620	ADCm.7.gabor.3.0.3.absmean.
621	ADCm.7.gabor.3.0.3.mag.
622	ADCm.7.gabor.3.0.4.mean.
623	ADCm.7.gabor.3.0.4.var.
624	ADCm.7.gabor.3.0.4.absmean.
625	ADCm.7.gabor.3.0.4.mag.
626	ADCm.7.gabor.3.0.5.mean.
627	ADCm.7.gabor.3.0.5.var.
628	ADCm.7.gabor.3.0.5.absmean.
629	ADCm.7.gabor.3.0.5.mag.
630	ADCm.9.gabor.1.0.1.mean.
631	ADCm.9.gabor.1.0.1.var.
632	ADCm.9.gabor.1.0.1.absmean.
633	ADCm.9.gabor.1.0.1.mag.
634	ADCm.9.gabor.1.0.2.mean.
635	ADCm.9.gabor.1.0.2.var.
636	ADCm.9.gabor.1.0.2.absmean.
637	ADCm.9.gabor.1.0.2.mag.
638	ADCm.9.gabor.1.0.3.mean.
639	ADCm.9.gabor.1.0.3.var.
640	ADCm.9.gabor.1.0.3.absmean.
641	ADCm.9.gabor.1.0.3.mag.
642	ADCm.9.gabor.1.0.4.mean.
643	ADCm.9.gabor.1.0.4.var.
644	ADCm.9.gabor.1.0.4.absmean.
645	ADCm.9.gabor.1.0.4.mag.
646	ADCm.9.gabor.1.0.5.mean.

647	ADCm.9.gabor.1.0.5.var.
648	ADCm.9.gabor.1.0.5.absmean.
649	ADCm.9.gabor.1.0.5.mag.
650	ADCm.9.gabor.2.0.1.mean.
651	ADCm.9.gabor.2.0.1.var.
652	ADCm.9.gabor.2.0.1.absmean.
653	ADCm.9.gabor.2.0.1.mag.
654	ADCm.9.gabor.2.0.2.mean.
655	ADCm.9.gabor.2.0.2.var.
656	ADCm.9.gabor.2.0.2.absmean.
657	ADCm.9.gabor.2.0.2.mag.
658	ADCm.9.gabor.2.0.3.mean.
659	ADCm.9.gabor.2.0.3.var.
660	ADCm.9.gabor.2.0.3.absmean.
661	ADCm.9.gabor.2.0.3.mag.
662	ADCm.9.gabor.2.0.4.mean.
663	ADCm.9.gabor.2.0.4.var.
664	ADCm.9.gabor.2.0.4.absmean.
665	ADCm.9.gabor.2.0.4.mag.
666	ADCm.9.gabor.2.0.5.mean.
667	ADCm.9.gabor.2.0.5.var.
668	ADCm.9.gabor.2.0.5.absmean.
669	ADCm.9.gabor.2.0.5.mag.
670	ADCm.9.gabor.3.0.1.mean.
671	ADCm.9.gabor.3.0.1.var.
672	ADCm.9.gabor.3.0.1.absmean.
673	ADCm.9.gabor.3.0.1.mag.
674	ADCm.9.gabor.3.0.2.mean.
675	ADCm.9.gabor.3.0.2.var.
676	ADCm.9.gabor.3.0.2.absmean.
677	ADCm.9.gabor.3.0.2.mag.
678	ADCm.9.gabor.3.0.3.mean.
679	ADCm.9.gabor.3.0.3.var.
680	ADCm.9.gabor.3.0.3.absmean.
681	ADCm.9.gabor.3.0.3.mag.
682	ADCm.9.gabor.3.0.4.mean.
683	ADCm.9.gabor.3.0.4.var.

684	ADCm.9.gabor.3.0.4.absmean.
685	ADCm.9.gabor.3.0.4.mag.
686	ADCm.9.gabor.3.0.5.mean.
687	ADCm.9.gabor.3.0.5.var.
688	ADCm.9.gabor.3.0.5.absmean.
689	ADCm.9.gabor.3.0.5.mag.
690	ADCm.11.gabor.1.0.1.mean.
691	ADCm.11.gabor.1.0.1.var.
692	ADCm.11.gabor.1.0.1.absmean.
693	ADCm.11.gabor.1.0.1.mag.
694	ADCm.11.gabor.1.0.2.mean.
695	ADCm.11.gabor.1.0.2.var.
696	ADCm.11.gabor.1.0.2.absmean.
697	ADCm.11.gabor.1.0.2.mag.
698	ADCm.11.gabor.1.0.3.mean.
699	ADCm.11.gabor.1.0.3.var.
700	ADCm.11.gabor.1.0.3.absmean.
701	ADCm.11.gabor.1.0.3.mag.
702	ADCm.11.gabor.1.0.4.mean.
703	ADCm.11.gabor.1.0.4.var.
704	ADCm.11.gabor.1.0.4.absmean.
705	ADCm.11.gabor.1.0.4.mag.
706	ADCm.11.gabor.1.0.5.mean.
707	ADCm.11.gabor.1.0.5.var.
708	ADCm.11.gabor.1.0.5.absmean.
709	ADCm.11.gabor.1.0.5.mag.
710	ADCm.11.gabor.2.0.1.mean.
711	ADCm.11.gabor.2.0.1.var.
712	ADCm.11.gabor.2.0.1.absmean.
713	ADCm.11.gabor.2.0.1.mag.
714	ADCm.11.gabor.2.0.2.mean.
715	ADCm.11.gabor.2.0.2.var.
716	ADCm.11.gabor.2.0.2.absmean.
717	ADCm.11.gabor.2.0.2.mag.
718	ADCm.11.gabor.2.0.3.mean.
719	ADCm.11.gabor.2.0.3.var.
720	ADCm.11.gabor.2.0.3.absmean.

721	ADCm.11.gabor.2.0.3.mag.
722	ADCm.11.gabor.2.0.4.mean.
723	ADCm.11.gabor.2.0.4.var.
724	ADCm.11.gabor.2.0.4.absmean.
725	ADCm.11.gabor.2.0.4.mag.
726	ADCm.11.gabor.2.0.5.mean.
727	ADCm.11.gabor.2.0.5.var.
728	ADCm.11.gabor.2.0.5.absmean.
729	ADCm.11.gabor.2.0.5.mag.
730	ADCm.11.gabor.3.0.1.mean.
731	ADCm.11.gabor.3.0.1.var.
732	ADCm.11.gabor.3.0.1.absmean.
733	ADCm.11.gabor.3.0.1.mag.
734	ADCm.11.gabor.3.0.2.mean.
735	ADCm.11.gabor.3.0.2.var.
736	ADCm.11.gabor.3.0.2.absmean.
737	ADCm.11.gabor.3.0.2.mag.
738	ADCm.11.gabor.3.0.3.mean.
739	ADCm.11.gabor.3.0.3.var.
740	ADCm.11.gabor.3.0.3.absmean.
741	ADCm.11.gabor.3.0.3.mag.
742	ADCm.11.gabor.3.0.4.mean.
743	ADCm.11.gabor.3.0.4.var.
744	ADCm.11.gabor.3.0.4.absmean.
745	ADCm.11.gabor.3.0.4.mag.
746	ADCm.11.gabor.3.0.5.mean.
747	ADCm.11.gabor.3.0.5.var.
748	ADCm.11.gabor.3.0.5.absmean.
749	ADCm.11.gabor.3.0.5.mag.
750	ADCm.13.gabor.1.0.1.mean.
751	ADCm.13.gabor.1.0.1.var.
752	ADCm.13.gabor.1.0.1.absmean.
753	ADCm.13.gabor.1.0.1.mag.
754	ADCm.13.gabor.1.0.2.mean.
755	ADCm.13.gabor.1.0.2.var.
756	ADCm.13.gabor.1.0.2.absmean.
757	ADCm.13.gabor.1.0.2.mag.

758	ADCm.13.gabor.1.0.3.mean.
759	ADCm.13.gabor.1.0.3.var.
760	ADCm.13.gabor.1.0.3.absmean.
761	ADCm.13.gabor.1.0.3.mag.
762	ADCm.13.gabor.1.0.4.mean.
763	ADCm.13.gabor.1.0.4.var.
764	ADCm.13.gabor.1.0.4.absmean.
765	ADCm.13.gabor.1.0.4.mag.
766	ADCm.13.gabor.1.0.5.mean.
767	ADCm.13.gabor.1.0.5.var.
768	ADCm.13.gabor.1.0.5.absmean.
769	ADCm.13.gabor.1.0.5.mag.
770	ADCm.13.gabor.2.0.1.mean.
771	ADCm.13.gabor.2.0.1.var.
772	ADCm.13.gabor.2.0.1.absmean.
773	ADCm.13.gabor.2.0.1.mag.
774	ADCm.13.gabor.2.0.2.mean.
775	ADCm.13.gabor.2.0.2.var.
776	ADCm.13.gabor.2.0.2.absmean.
777	ADCm.13.gabor.2.0.2.mag.
778	ADCm.13.gabor.2.0.3.mean.
779	ADCm.13.gabor.2.0.3.var.
780	ADCm.13.gabor.2.0.3.absmean.
781	ADCm.13.gabor.2.0.3.mag.
782	ADCm.13.gabor.2.0.4.mean.
783	ADCm.13.gabor.2.0.4.var.
784	ADCm.13.gabor.2.0.4.absmean.
785	ADCm.13.gabor.2.0.4.mag.
786	ADCm.13.gabor.2.0.5.mean.
787	ADCm.13.gabor.2.0.5.var.
788	ADCm.13.gabor.2.0.5.absmean.
789	ADCm.13.gabor.2.0.5.mag.
790	ADCm.13.gabor.3.0.1.mean.
791	ADCm.13.gabor.3.0.1.var.
792	ADCm.13.gabor.3.0.1.absmean.
793	ADCm.13.gabor.3.0.1.mag.
794	ADCm.13.gabor.3.0.2.mean.

795	ADCm.13.gabor.3.0.2.var.
796	ADCm.13.gabor.3.0.2.absmean.
797	ADCm.13.gabor.3.0.2.mag.
798	ADCm.13.gabor.3.0.3.mean.
799	ADCm.13.gabor.3.0.3.var.
800	ADCm.13.gabor.3.0.3.absmean.
801	ADCm.13.gabor.3.0.3.mag.
802	ADCm.13.gabor.3.0.4.mean.
803	ADCm.13.gabor.3.0.4.var.
804	ADCm.13.gabor.3.0.4.absmean.
805	ADCm.13.gabor.3.0.4.mag.
806	ADCm.13.gabor.3.0.5.mean.
807	ADCm.13.gabor.3.0.5.var.
808	ADCm.13.gabor.3.0.5.absmean.
809	ADCm.13.gabor.3.0.5.mag.
810	ADCm.15.gabor.1.0.1.mean.
811	ADCm.15.gabor.1.0.1.var.
812	ADCm.15.gabor.1.0.1.absmean.
813	ADCm.15.gabor.1.0.1.mag.
814	ADCm.15.gabor.1.0.2.mean.
815	ADCm.15.gabor.1.0.2.var.
816	ADCm.15.gabor.1.0.2.absmean.
817	ADCm.15.gabor.1.0.2.mag.
818	ADCm.15.gabor.1.0.3.mean.
819	ADCm.15.gabor.1.0.3.var.
820	ADCm.15.gabor.1.0.3.absmean.
821	ADCm.15.gabor.1.0.3.mag.
822	ADCm.15.gabor.1.0.4.mean.
823	ADCm.15.gabor.1.0.4.var.
824	ADCm.15.gabor.1.0.4.absmean.
825	ADCm.15.gabor.1.0.4.mag.
826	ADCm.15.gabor.1.0.5.mean.
827	ADCm.15.gabor.1.0.5.var.
828	ADCm.15.gabor.1.0.5.absmean.
829	ADCm.15.gabor.1.0.5.mag.
830	ADCm.15.gabor.2.0.1.mean.
831	ADCm.15.gabor.2.0.1.var.

832	ADCm.15.gabor.2.0.1.absmean.
833	ADCm.15.gabor.2.0.1.mag.
834	ADCm.15.gabor.2.0.2.mean.
835	ADCm.15.gabor.2.0.2.var.
836	ADCm.15.gabor.2.0.2.absmean.
837	ADCm.15.gabor.2.0.2.mag.
838	ADCm.15.gabor.2.0.3.mean.
839	ADCm.15.gabor.2.0.3.var.
840	ADCm.15.gabor.2.0.3.absmean.
841	ADCm.15.gabor.2.0.3.mag.
842	ADCm.15.gabor.2.0.4.mean.
843	ADCm.15.gabor.2.0.4.var.
844	ADCm.15.gabor.2.0.4.absmean.
845	ADCm.15.gabor.2.0.4.mag.
846	ADCm.15.gabor.2.0.5.mean.
847	ADCm.15.gabor.2.0.5.var.
848	ADCm.15.gabor.2.0.5.absmean.
849	ADCm.15.gabor.2.0.5.mag.
850	ADCm.15.gabor.3.0.1.mean.
851	ADCm.15.gabor.3.0.1.var.
852	ADCm.15.gabor.3.0.1.absmean.
853	ADCm.15.gabor.3.0.1.mag.
854	ADCm.15.gabor.3.0.2.mean.
855	ADCm.15.gabor.3.0.2.var.
856	ADCm.15.gabor.3.0.2.absmean.
857	ADCm.15.gabor.3.0.2.mag.
858	ADCm.15.gabor.3.0.3.mean.
859	ADCm.15.gabor.3.0.3.var.
860	ADCm.15.gabor.3.0.3.absmean.
861	ADCm.15.gabor.3.0.3.mag.
862	ADCm.15.gabor.3.0.4.mean.
863	ADCm.15.gabor.3.0.4.var.
864	ADCm.15.gabor.3.0.4.absmean.
865	ADCm.15.gabor.3.0.4.mag.
866	ADCm.15.gabor.3.0.5.mean.
867	ADCm.15.gabor.3.0.5.var.
868	ADCm.15.gabor.3.0.5.absmean.

869	ADCm.15.gabor.3.0.5.mag.
870	ADCm.3.haar.1.1.aav.
871	ADCm.3.haar.1.1.std.
872	ADCm.3.haar.1.2.aav.
873	ADCm.3.haar.1.2.std.
874	ADCm.3.haar.1.3.aav.
875	ADCm.3.haar.1.3.std.
876	ADCm.3.haar.2.1.aav.
877	ADCm.3.haar.2.1.std.
878	ADCm.3.haar.2.2.aav.
879	ADCm.3.haar.2.2.std.
880	ADCm.3.haar.2.3.aav.
881	ADCm.3.haar.2.3.std.
882	ADCm.3.haar.3.1.aav.
883	ADCm.3.haar.3.1.std.
884	ADCm.3.haar.3.2.aav.
885	ADCm.3.haar.3.2.std.
886	ADCm.3.haar.3.3.aav.
887	ADCm.3.haar.3.3.std.
888	ADCm.3.haar.4.1.aav.
889	ADCm.3.haar.4.1.std.
890	ADCm.3.haar.4.2.aav.
891	ADCm.3.haar.4.2.std.
892	ADCm.3.haar.4.3.aav.
893	ADCm.3.haar.4.3.std.
894	ADCm.5.haar.1.1.aav.
895	ADCm.5.haar.1.1.std.
896	ADCm.5.haar.1.2.aav.
897	ADCm.5.haar.1.2.std.
898	ADCm.5.haar.1.3.aav.
899	ADCm.5.haar.1.3.std.
900	ADCm.5.haar.2.1.aav.
901	ADCm.5.haar.2.1.std.
902	ADCm.5.haar.2.2.aav.
903	ADCm.5.haar.2.2.std.
904	ADCm.5.haar.2.3.aav.
905	ADCm.5.haar.2.3.std.

906	ADCm.5.haar.3.1.aav.
907	ADCm.5.haar.3.1.std.
908	ADCm.5.haar.3.2.aav.
909	ADCm.5.haar.3.2.std.
910	ADCm.5.haar.3.3.aav.
911	ADCm.5.haar.3.3.std.
912	ADCm.5.haar.4.1.aav.
913	ADCm.5.haar.4.1.std.
914	ADCm.5.haar.4.2.aav.
915	ADCm.5.haar.4.2.std.
916	ADCm.5.haar.4.3.aav.
917	ADCm.5.haar.4.3.std.
918	ADCm.7.haar.1.1.aav.
919	ADCm.7.haar.1.1.std.
920	ADCm.7.haar.1.2.aav.
921	ADCm.7.haar.1.2.std.
922	ADCm.7.haar.1.3.aav.
923	ADCm.7.haar.1.3.std.
924	ADCm.7.haar.2.1.aav.
925	ADCm.7.haar.2.1.std.
926	ADCm.7.haar.2.2.aav.
927	ADCm.7.haar.2.2.std.
928	ADCm.7.haar.2.3.aav.
929	ADCm.7.haar.2.3.std.
930	ADCm.7.haar.3.1.aav.
931	ADCm.7.haar.3.1.std.
932	ADCm.7.haar.3.2.aav.
933	ADCm.7.haar.3.2.std.
934	ADCm.7.haar.3.3.aav.
935	ADCm.7.haar.3.3.std.
936	ADCm.7.haar.4.1.aav.
937	ADCm.7.haar.4.1.std.
938	ADCm.7.haar.4.2.aav.
939	ADCm.7.haar.4.2.std.
940	ADCm.7.haar.4.3.aav.
941	ADCm.7.haar.4.3.std.
942	ADCm.9.haar.1.1.aav.

943	ADCm.9.haar.1.1.std.
944	ADCm.9.haar.1.2.aav.
945	ADCm.9.haar.1.2.std.
946	ADCm.9.haar.1.3.aav.
947	ADCm.9.haar.1.3.std.
948	ADCm.9.haar.2.1.aav.
949	ADCm.9.haar.2.1.std.
950	ADCm.9.haar.2.2.aav.
951	ADCm.9.haar.2.2.std.
952	ADCm.9.haar.2.3.aav.
953	ADCm.9.haar.2.3.std.
954	ADCm.9.haar.3.1.aav.
955	ADCm.9.haar.3.1.std.
956	ADCm.9.haar.3.2.aav.
957	ADCm.9.haar.3.2.std.
958	ADCm.9.haar.3.3.aav.
959	ADCm.9.haar.3.3.std.
960	ADCm.9.haar.4.1.aav.
961	ADCm.9.haar.4.1.std.
962	ADCm.9.haar.4.2.aav.
963	ADCm.9.haar.4.2.std.
964	ADCm.9.haar.4.3.aav.
965	ADCm.9.haar.4.3.std.
966	ADCm.11.haar.1.1.aav.
967	ADCm.11.haar.1.1.std.
968	ADCm.11.haar.1.2.aav.
969	ADCm.11.haar.1.2.std.
970	ADCm.11.haar.1.3.aav.
971	ADCm.11.haar.1.3.std.
972	ADCm.11.haar.2.1.aav.
973	ADCm.11.haar.2.1.std.
974	ADCm.11.haar.2.2.aav.
975	ADCm.11.haar.2.2.std.
976	ADCm.11.haar.2.3.aav.
977	ADCm.11.haar.2.3.std.
978	ADCm.11.haar.3.1.aav.
979	ADCm.11.haar.3.1.std.

980	ADCm.11.haar.3.2.aav.
981	ADCm.11.haar.3.2.std.
982	ADCm.11.haar.3.3.aav.
983	ADCm.11.haar.3.3.std.
984	ADCm.11.haar.4.1.aav.
985	ADCm.11.haar.4.1.std.
986	ADCm.11.haar.4.2.aav.
987	ADCm.11.haar.4.2.std.
988	ADCm.11.haar.4.3.aav.
989	ADCm.11.haar.4.3.std.
990	ADCm.13.haar.1.1.aav.
991	ADCm.13.haar.1.1.std.
992	ADCm.13.haar.1.2.aav.
993	ADCm.13.haar.1.2.std.
994	ADCm.13.haar.1.3.aav.
995	ADCm.13.haar.1.3.std.
996	ADCm.13.haar.2.1.aav.
997	ADCm.13.haar.2.1.std.
998	ADCm.13.haar.2.2.aav.
999	ADCm.13.haar.2.2.std.
1000	ADCm.13.haar.2.3.aav.
1001	ADCm.13.haar.2.3.std.
1002	ADCm.13.haar.3.1.aav.
1003	ADCm.13.haar.3.1.std.
1004	ADCm.13.haar.3.2.aav.
1005	ADCm.13.haar.3.2.std.
1006	ADCm.13.haar.3.3.aav.
1007	ADCm.13.haar.3.3.std.
1008	ADCm.13.haar.4.1.aav.
1009	ADCm.13.haar.4.1.std.
1010	ADCm.13.haar.4.2.aav.
1011	ADCm.13.haar.4.2.std.
1012	ADCm.13.haar.4.3.aav.
1013	ADCm.13.haar.4.3.std.
1014	ADCm.15.haar.1.1.aav.
1015	ADCm.15.haar.1.1.std.
1016	ADCm.15.haar.1.2.aav.

1017	ADCm.15.haar.1.2.std.
1018	ADCm.15.haar.1.3.aav.
1019	ADCm.15.haar.1.3.std.
1020	ADCm.15.haar.2.1.aav.
1021	ADCm.15.haar.2.1.std.
1022	ADCm.15.haar.2.2.aav.
1023	ADCm.15.haar.2.2.std.
1024	ADCm.15.haar.2.3.aav.
1025	ADCm.15.haar.2.3.std.
1026	ADCm.15.haar.3.1.aav.
1027	ADCm.15.haar.3.1.std.
1028	ADCm.15.haar.3.2.aav.
1029	ADCm.15.haar.3.2.std.
1030	ADCm.15.haar.3.3.aav.
1031	ADCm.15.haar.3.3.std.
1032	ADCm.15.haar.4.1.aav.
1033	ADCm.15.haar.4.1.std.
1034	ADCm.15.haar.4.2.aav.
1035	ADCm.15.haar.4.2.std.
1036	ADCm.15.haar.4.3.aav.
1037	ADCm.15.haar.4.3.std.
1038	ADCm.3.hu.0.
1039	ADCm.3.hu.1.
1040	ADCm.3.hu.2.
1041	ADCm.3.hu.3.
1042	ADCm.3.hu.4.
1043	ADCm.3.hu.5.
1044	ADCm.3.hu.6.
1045	ADCm.5.hu.0.
1046	ADCm.5.hu.1.
1047	ADCm.5.hu.2.
1048	ADCm.5.hu.3.
1049	ADCm.5.hu.4.
1050	ADCm.5.hu.5.
1051	ADCm.5.hu.6.
1052	ADCm.7.hu.0.
1053	ADCm.7.hu.1.

1054	ADCm.7.hu.2.
1055	ADCm.7.hu.3.
1056	ADCm.7.hu.4.
1057	ADCm.7.hu.5.
1058	ADCm.7.hu.6.
1059	ADCm.9.hu.0.
1060	ADCm.9.hu.1.
1061	ADCm.9.hu.2.
1062	ADCm.9.hu.3.
1063	ADCm.9.hu.4.
1064	ADCm.9.hu.5.
1065	ADCm.9.hu.6.
1066	ADCm.11.hu.0.
1067	ADCm.11.hu.1.
1068	ADCm.11.hu.2.
1069	ADCm.11.hu.3.
1070	ADCm.11.hu.4.
1071	ADCm.11.hu.5.
1072	ADCm.11.hu.6.
1073	ADCm.13.hu.0.
1074	ADCm.13.hu.1.
1075	ADCm.13.hu.2.
1076	ADCm.13.hu.3.
1077	ADCm.13.hu.4.
1078	ADCm.13.hu.5.
1079	ADCm.13.hu.6.
1080	ADCm.15.hu.0.
1081	ADCm.15.hu.1.
1082	ADCm.15.hu.2.
1083	ADCm.15.hu.3.
1084	ADCm.15.hu.4.
1085	ADCm.15.hu.5.
1086	ADCm.15.hu.6.
1087	ADCm.3.zernike.0.
1088	ADCm.3.zernike.1.
1089	ADCm.3.zernike.2.
1090	ADCm.3.zernike.3.

1091	ADCm.3.zernike.4.
1092	ADCm.3.zernike.5.
1093	ADCm.3.zernike.6.
1094	ADCm.3.zernike.7.
1095	ADCm.3.zernike.8.
1096	ADCm.3.zernike.9.
1097	ADCm.3.zernike.10.
1098	ADCm.3.zernike.11.
1099	ADCm.3.zernike.12.
1100	ADCm.3.zernike.13.
1101	ADCm.3.zernike.14.
1102	ADCm.3.zernike.15.
1103	ADCm.3.zernike.16.
1104	ADCm.3.zernike.17.
1105	ADCm.3.zernike.18.
1106	ADCm.3.zernike.19.
1107	ADCm.3.zernike.20.
1108	ADCm.3.zernike.21.
1109	ADCm.3.zernike.22.
1110	ADCm.3.zernike.23.
1111	ADCm.3.zernike.24.
1112	ADCm.5.zernike.0.
1113	ADCm.5.zernike.1.
1114	ADCm.5.zernike.2.
1115	ADCm.5.zernike.3.
1116	ADCm.5.zernike.4.
1117	ADCm.5.zernike.5.
1118	ADCm.5.zernike.6.
1119	ADCm.5.zernike.7.
1120	ADCm.5.zernike.8.
1121	ADCm.5.zernike.9.
1122	ADCm.5.zernike.10.
1123	ADCm.5.zernike.11.
1124	ADCm.5.zernike.12.
1125	ADCm.5.zernike.13.
1126	ADCm.5.zernike.14.
1127	ADCm.5.zernike.15.

1128	ADCm.5.zernike.16.
1129	ADCm.5.zernike.17.
1130	ADCm.5.zernike.18.
1131	ADCm.5.zernike.19.
1132	ADCm.5.zernike.20.
1133	ADCm.5.zernike.21.
1134	ADCm.5.zernike.22.
1135	ADCm.5.zernike.23.
1136	ADCm.5.zernike.24.
1137	ADCm.7.zernike.0.
1138	ADCm.7.zernike.1.
1139	ADCm.7.zernike.2.
1140	ADCm.7.zernike.3.
1141	ADCm.7.zernike.4.
1142	ADCm.7.zernike.5.
1143	ADCm.7.zernike.6.
1144	ADCm.7.zernike.7.
1145	ADCm.7.zernike.8.
1146	ADCm.7.zernike.9.
1147	ADCm.7.zernike.10.
1148	ADCm.7.zernike.11.
1149	ADCm.7.zernike.12.
1150	ADCm.7.zernike.13.
1151	ADCm.7.zernike.14.
1152	ADCm.7.zernike.15.
1153	ADCm.7.zernike.16.
1154	ADCm.7.zernike.17.
1155	ADCm.7.zernike.18.
1156	ADCm.7.zernike.19.
1157	ADCm.7.zernike.20.
1158	ADCm.7.zernike.21.
1159	ADCm.7.zernike.22.
1160	ADCm.7.zernike.23.
1161	ADCm.7.zernike.24.
1162	ADCm.9.zernike.0.
1163	ADCm.9.zernike.1.
1164	ADCm.9.zernike.2.

1165	ADCm.9.zernike.3.
1166	ADCm.9.zernike.4.
1167	ADCm.9.zernike.5.
1168	ADCm.9.zernike.6.
1169	ADCm.9.zernike.7.
1170	ADCm.9.zernike.8.
1171	ADCm.9.zernike.9.
1172	ADCm.9.zernike.10.
1173	ADCm.9.zernike.11.
1174	ADCm.9.zernike.12.
1175	ADCm.9.zernike.13.
1176	ADCm.9.zernike.14.
1177	ADCm.9.zernike.15.
1178	ADCm.9.zernike.16.
1179	ADCm.9.zernike.17.
1180	ADCm.9.zernike.18.
1181	ADCm.9.zernike.19.
1182	ADCm.9.zernike.20.
1183	ADCm.9.zernike.21.
1184	ADCm.9.zernike.22.
1185	ADCm.9.zernike.23.
1186	ADCm.9.zernike.24.
1187	ADCm.11.zernike.0.
1188	ADCm.11.zernike.1.
1189	ADCm.11.zernike.2.
1190	ADCm.11.zernike.3.
1191	ADCm.11.zernike.4.
1192	ADCm.11.zernike.5.
1193	ADCm.11.zernike.6.
1194	ADCm.11.zernike.7.
1195	ADCm.11.zernike.8.
1196	ADCm.11.zernike.9.
1197	ADCm.11.zernike.10.
1198	ADCm.11.zernike.11.
1199	ADCm.11.zernike.12.
1200	ADCm.11.zernike.13.
1201	ADCm.11.zernike.14.

1202	ADCm.11.zernike.15.
1203	ADCm.11.zernike.16.
1204	ADCm.11.zernike.17.
1205	ADCm.11.zernike.18.
1206	ADCm.11.zernike.19.
1207	ADCm.11.zernike.20.
1208	ADCm.11.zernike.21.
1209	ADCm.11.zernike.22.
1210	ADCm.11.zernike.23.
1211	ADCm.11.zernike.24.
1212	ADCm.13.zernike.0.
1213	ADCm.13.zernike.1.
1214	ADCm.13.zernike.2.
1215	ADCm.13.zernike.3.
1216	ADCm.13.zernike.4.
1217	ADCm.13.zernike.5.
1218	ADCm.13.zernike.6.
1219	ADCm.13.zernike.7.
1220	ADCm.13.zernike.8.
1221	ADCm.13.zernike.9.
1222	ADCm.13.zernike.10.
1223	ADCm.13.zernike.11.
1224	ADCm.13.zernike.12.
1225	ADCm.13.zernike.13.
1226	ADCm.13.zernike.14.
1227	ADCm.13.zernike.15.
1228	ADCm.13.zernike.16.
1229	ADCm.13.zernike.17.
1230	ADCm.13.zernike.18.
1231	ADCm.13.zernike.19.
1232	ADCm.13.zernike.20.
1233	ADCm.13.zernike.21.
1234	ADCm.13.zernike.22.
1235	ADCm.13.zernike.23.
1236	ADCm.13.zernike.24.
1237	ADCm.15.zernike.0.
1238	ADCm.15.zernike.1.

1239	ADCm.15.zernike.2.
1240	ADCm.15.zernike.3.
1241	ADCm.15.zernike.4.
1242	ADCm.15.zernike.5.
1243	ADCm.15.zernike.6.
1244	ADCm.15.zernike.7.
1245	ADCm.15.zernike.8.
1246	ADCm.15.zernike.9.
1247	ADCm.15.zernike.10.
1248	ADCm.15.zernike.11.
1249	ADCm.15.zernike.12.
1250	ADCm.15.zernike.13.
1251	ADCm.15.zernike.14.
1252	ADCm.15.zernike.15.
1253	ADCm.15.zernike.16.
1254	ADCm.15.zernike.17.
1255	ADCm.15.zernike.18.
1256	ADCm.15.zernike.19.
1257	ADCm.15.zernike.20.
1258	ADCm.15.zernike.21.
1259	ADCm.15.zernike.22.
1260	ADCm.15.zernike.23.
1261	ADCm.15.zernike.24.
1262	ADCm.3.sobel
1263	ADCm.3.sobel_mask
1264	ADCm.all.stats.p000.
1265	ADCm.all.stats.p010.
1266	ADCm.all.stats.p020.
1267	ADCm.all.stats.p025.
1268	ADCm.all.stats.p030.
1269	ADCm.all.stats.p040.
1270	ADCm.all.stats.p050.
1271	ADCm.all.stats.p060.
1272	ADCm.all.stats.p070.
1273	ADCm.all.stats.p075.
1274	ADCm.all.stats.p080.
1275	ADCm.all.stats.p090.

1276	ADCm.all.stats.p100.
1277	ADCm.all.stats.range.
1278	ADCm.all.stats.mean.
1279	ADCm.all.stats.stddev.
1280	ADCm.all.stats.kurtosis.
1281	ADCm.all.stats.skewness.
1282	ADCk.3.glcm.contrast.1.mean.
1283	ADCk.3.glcm.contrast.1.range.
1284	ADCk.3.glcm.contrast.2.mean.
1285	ADCk.3.glcm.contrast.2.range.
1286	ADCk.3.glcm.contrast.3.mean.
1287	ADCk.3.glcm.contrast.3.range.
1288	ADCk.3.glcm.dissimilarity.1.mean.
1289	ADCk.3.glcm.dissimilarity.1.range.
1290	ADCk.3.glcm.dissimilarity.2.mean.
1291	ADCk.3.glcm.dissimilarity.2.range.
1292	ADCk.3.glcm.dissimilarity.3.mean.
1293	ADCk.3.glcm.dissimilarity.3.range.
1294	ADCk.3.glcm.homogeneity.1.mean.
1295	ADCk.3.glcm.homogeneity.1.range.
1296	ADCk.3.glcm.homogeneity.2.mean.
1297	ADCk.3.glcm.homogeneity.2.range.
1298	ADCk.3.glcm.homogeneity.3.mean.
1299	ADCk.3.glcm.homogeneity.3.range.
1300	ADCk.3.glcm.energy.1.mean.
1301	ADCk.3.glcm.energy.1.range.
1302	ADCk.3.glcm.energy.2.mean.
1303	ADCk.3.glcm.energy.2.range.
1304	ADCk.3.glcm.energy.3.mean.
1305	ADCk.3.glcm.energy.3.range.
1306	ADCk.3.glcm.correlation.1.mean.
1307	ADCk.3.glcm.correlation.1.range.
1308	ADCk.3.glcm.correlation.2.mean.
1309	ADCk.3.glcm.correlation.2.range.
1310	ADCk.3.glcm.correlation.3.mean.
1311	ADCk.3.glcm.correlation.3.range.
1312	ADCk.3.glcm.ASM.1.mean.

1313	ADCk.3.glcm.ASM.1.range.
1314	ADCk.3.glcm.ASM.2.mean.
1315	ADCk.3.glcm.ASM.2.range.
1316	ADCk.3.glcm.ASM.3.mean.
1317	ADCk.3.glcm.ASM.3.range.
1318	ADCk.5.glcm.contrast.1.mean.
1319	ADCk.5.glcm.contrast.1.range.
1320	ADCk.5.glcm.contrast.2.mean.
1321	ADCk.5.glcm.contrast.2.range.
1322	ADCk.5.glcm.contrast.3.mean.
1323	ADCk.5.glcm.contrast.3.range.
1324	ADCk.5.glcm.contrast.4.mean.
1325	ADCk.5.glcm.contrast.4.range.
1326	ADCk.5.glcm.dissimilarity.1.mean.
1327	ADCk.5.glcm.dissimilarity.1.range.
1328	ADCk.5.glcm.dissimilarity.2.mean.
1329	ADCk.5.glcm.dissimilarity.2.range.
1330	ADCk.5.glcm.dissimilarity.3.mean.
1331	ADCk.5.glcm.dissimilarity.3.range.
1332	ADCk.5.glcm.dissimilarity.4.mean.
1333	ADCk.5.glcm.dissimilarity.4.range.
1334	ADCk.5.glcm.homogeneity.1.mean.
1335	ADCk.5.glcm.homogeneity.1.range.
1336	ADCk.5.glcm.homogeneity.2.mean.
1337	ADCk.5.glcm.homogeneity.2.range.
1338	ADCk.5.glcm.homogeneity.3.mean.
1339	ADCk.5.glcm.homogeneity.3.range.
1340	ADCk.5.glcm.homogeneity.4.mean.
1341	ADCk.5.glcm.homogeneity.4.range.
1342	ADCk.5.glcm.energy.1.mean.
1343	ADCk.5.glcm.energy.1.range.
1344	ADCk.5.glcm.energy.2.mean.
1345	ADCk.5.glcm.energy.2.range.
1346	ADCk.5.glcm.energy.3.mean.
1347	ADCk.5.glcm.energy.3.range.
1348	ADCk.5.glcm.energy.4.mean.
1349	ADCk.5.glcm.energy.4.range.

1350	ADCk.5.glcm.correlation.1.mean.
1351	ADCk.5.glcm.correlation.1.range.
1352	ADCk.5.glcm.correlation.2.mean.
1353	ADCk.5.glcm.correlation.2.range.
1354	ADCk.5.glcm.correlation.3.mean.
1355	ADCk.5.glcm.correlation.3.range.
1356	ADCk.5.glcm.correlation.4.mean.
1357	ADCk.5.glcm.correlation.4.range.
1358	ADCk.5.glcm.ASM.1.mean.
1359	ADCk.5.glcm.ASM.1.range.
1360	ADCk.5.glcm.ASM.2.mean.
1361	ADCk.5.glcm.ASM.2.range.
1362	ADCk.5.glcm.ASM.3.mean.
1363	ADCk.5.glcm.ASM.3.range.
1364	ADCk.5.glcm.ASM.4.mean.
1365	ADCk.5.glcm.ASM.4.range.
1366	ADCk.7.glcm.contrast.1.mean.
1367	ADCk.7.glcm.contrast.1.range.
1368	ADCk.7.glcm.contrast.2.mean.
1369	ADCk.7.glcm.contrast.2.range.
1370	ADCk.7.glcm.contrast.3.mean.
1371	ADCk.7.glcm.contrast.3.range.
1372	ADCk.7.glcm.contrast.4.mean.
1373	ADCk.7.glcm.contrast.4.range.
1374	ADCk.7.glcm.dissimilarity.1.mean.
1375	ADCk.7.glcm.dissimilarity.1.range.
1376	ADCk.7.glcm.dissimilarity.2.mean.
1377	ADCk.7.glcm.dissimilarity.2.range.
1378	ADCk.7.glcm.dissimilarity.3.mean.
1379	ADCk.7.glcm.dissimilarity.3.range.
1380	ADCk.7.glcm.dissimilarity.4.mean.
1381	ADCk.7.glcm.dissimilarity.4.range.
1382	ADCk.7.glcm.homogeneity.1.mean.
1383	ADCk.7.glcm.homogeneity.1.range.
1384	ADCk.7.glcm.homogeneity.2.mean.
1385	ADCk.7.glcm.homogeneity.2.range.
1386	ADCk.7.glcm.homogeneity.3.mean.

1387	ADCk.7.glcm.homogeneity.3.range.
1388	ADCk.7.glcm.homogeneity.4.mean.
1389	ADCk.7.glcm.homogeneity.4.range.
1390	ADCk.7.glcm.energy.1.mean.
1391	ADCk.7.glcm.energy.1.range.
1392	ADCk.7.glcm.energy.2.mean.
1393	ADCk.7.glcm.energy.2.range.
1394	ADCk.7.glcm.energy.3.mean.
1395	ADCk.7.glcm.energy.3.range.
1396	ADCk.7.glcm.energy.4.mean.
1397	ADCk.7.glcm.energy.4.range.
1398	ADCk.7.glcm.correlation.1.mean.
1399	ADCk.7.glcm.correlation.1.range.
1400	ADCk.7.glcm.correlation.2.mean.
1401	ADCk.7.glcm.correlation.2.range.
1402	ADCk.7.glcm.correlation.3.mean.
1403	ADCk.7.glcm.correlation.3.range.
1404	ADCk.7.glcm.correlation.4.mean.
1405	ADCk.7.glcm.correlation.4.range.
1406	ADCk.7.glcm.ASM.1.mean.
1407	ADCk.7.glcm.ASM.1.range.
1408	ADCk.7.glcm.ASM.2.mean.
1409	ADCk.7.glcm.ASM.2.range.
1410	ADCk.7.glcm.ASM.3.mean.
1411	ADCk.7.glcm.ASM.3.range.
1412	ADCk.7.glcm.ASM.4.mean.
1413	ADCk.7.glcm.ASM.4.range.
1414	ADCk.9.glcm.contrast.1.mean.
1415	ADCk.9.glcm.contrast.1.range.
1416	ADCk.9.glcm.contrast.2.mean.
1417	ADCk.9.glcm.contrast.2.range.
1418	ADCk.9.glcm.contrast.3.mean.
1419	ADCk.9.glcm.contrast.3.range.
1420	ADCk.9.glcm.contrast.4.mean.
1421	ADCk.9.glcm.contrast.4.range.
1422	ADCk.9.glcm.dissimilarity.1.mean.
1423	ADCk.9.glcm.dissimilarity.1.range.

1424	ADCk.9.glcm.dissimilarity.2.mean.
1425	ADCk.9.glcm.dissimilarity.2.range.
1426	ADCk.9.glcm.dissimilarity.3.mean.
1427	ADCk.9.glcm.dissimilarity.3.range.
1428	ADCk.9.glcm.dissimilarity.4.mean.
1429	ADCk.9.glcm.dissimilarity.4.range.
1430	ADCk.9.glcm.homogeneity.1.mean.
1431	ADCk.9.glcm.homogeneity.1.range.
1432	ADCk.9.glcm.homogeneity.2.mean.
1433	ADCk.9.glcm.homogeneity.2.range.
1434	ADCk.9.glcm.homogeneity.3.mean.
1435	ADCk.9.glcm.homogeneity.3.range.
1436	ADCk.9.glcm.homogeneity.4.mean.
1437	ADCk.9.glcm.homogeneity.4.range.
1438	ADCk.9.glcm.energy.1.mean.
1439	ADCk.9.glcm.energy.1.range.
1440	ADCk.9.glcm.energy.2.mean.
1441	ADCk.9.glcm.energy.2.range.
1442	ADCk.9.glcm.energy.3.mean.
1443	ADCk.9.glcm.energy.3.range.
1444	ADCk.9.glcm.energy.4.mean.
1445	ADCk.9.glcm.energy.4.range.
1446	ADCk.9.glcm.correlation.1.mean.
1447	ADCk.9.glcm.correlation.1.range.
1448	ADCk.9.glcm.correlation.2.mean.
1449	ADCk.9.glcm.correlation.2.range.
1450	ADCk.9.glcm.correlation.3.mean.
1451	ADCk.9.glcm.correlation.3.range.
1452	ADCk.9.glcm.correlation.4.mean.
1453	ADCk.9.glcm.correlation.4.range.
1454	ADCk.9.glcm.ASM.1.mean.
1455	ADCk.9.glcm.ASM.1.range.
1456	ADCk.9.glcm.ASM.2.mean.
1457	ADCk.9.glcm.ASM.2.range.
1458	ADCk.9.glcm.ASM.3.mean.
1459	ADCk.9.glcm.ASM.3.range.
1460	ADCk.9.glcm.ASM.4.mean.

1461	ADCk.9.glcm.ASM.4.range.
1462	ADCk.11.glcm.contrast.1.mean.
1463	ADCk.11.glcm.contrast.1.range.
1464	ADCk.11.glcm.contrast.2.mean.
1465	ADCk.11.glcm.contrast.2.range.
1466	ADCk.11.glcm.contrast.3.mean.
1467	ADCk.11.glcm.contrast.3.range.
1468	ADCk.11.glcm.contrast.4.mean.
1469	ADCk.11.glcm.contrast.4.range.
1470	ADCk.11.glcm.dissimilarity.1.mean.
1471	ADCk.11.glcm.dissimilarity.1.range.
1472	ADCk.11.glcm.dissimilarity.2.mean.
1473	ADCk.11.glcm.dissimilarity.2.range.
1474	ADCk.11.glcm.dissimilarity.3.mean.
1475	ADCk.11.glcm.dissimilarity.3.range.
1476	ADCk.11.glcm.dissimilarity.4.mean.
1477	ADCk.11.glcm.dissimilarity.4.range.
1478	ADCk.11.glcm.homogeneity.1.mean.
1479	ADCk.11.glcm.homogeneity.1.range.
1480	ADCk.11.glcm.homogeneity.2.mean.
1481	ADCk.11.glcm.homogeneity.2.range.
1482	ADCk.11.glcm.homogeneity.3.mean.
1483	ADCk.11.glcm.homogeneity.3.range.
1484	ADCk.11.glcm.homogeneity.4.mean.
1485	ADCk.11.glcm.homogeneity.4.range.
1486	ADCk.11.glcm.energy.1.mean.
1487	ADCk.11.glcm.energy.1.range.
1488	ADCk.11.glcm.energy.2.mean.
1489	ADCk.11.glcm.energy.2.range.
1490	ADCk.11.glcm.energy.3.mean.
1491	ADCk.11.glcm.energy.3.range.
1492	ADCk.11.glcm.energy.4.mean.
1493	ADCk.11.glcm.energy.4.range.
1494	ADCk.11.glcm.correlation.1.mean.
1495	ADCk.11.glcm.correlation.1.range.
1496	ADCk.11.glcm.correlation.2.mean.
1497	ADCk.11.glcm.correlation.2.range.

1498	ADCk.11.glcm.correlation.3.mean.
1499	ADCk.11.glcm.correlation.3.range.
1500	ADCk.11.glcm.correlation.4.mean.
1501	ADCk.11.glcm.correlation.4.range.
1502	ADCk.11.glcm.ASM.1.mean.
1503	ADCk.11.glcm.ASM.1.range.
1504	ADCk.11.glcm.ASM.2.mean.
1505	ADCk.11.glcm.ASM.2.range.
1506	ADCk.11.glcm.ASM.3.mean.
1507	ADCk.11.glcm.ASM.3.range.
1508	ADCk.11.glcm.ASM.4.mean.
1509	ADCk.11.glcm.ASM.4.range.
1510	ADCk.13.glcm.contrast.1.mean.
1511	ADCk.13.glcm.contrast.1.range.
1512	ADCk.13.glcm.contrast.2.mean.
1513	ADCk.13.glcm.contrast.2.range.
1514	ADCk.13.glcm.contrast.3.mean.
1515	ADCk.13.glcm.contrast.3.range.
1516	ADCk.13.glcm.contrast.4.mean.
1517	ADCk.13.glcm.contrast.4.range.
1518	ADCk.13.glcm.dissimilarity.1.mean.
1519	ADCk.13.glcm.dissimilarity.1.range.
1520	ADCk.13.glcm.dissimilarity.2.mean.
1521	ADCk.13.glcm.dissimilarity.2.range.
1522	ADCk.13.glcm.dissimilarity.3.mean.
1523	ADCk.13.glcm.dissimilarity.3.range.
1524	ADCk.13.glcm.dissimilarity.4.mean.
1525	ADCk.13.glcm.dissimilarity.4.range.
1526	ADCk.13.glcm.homogeneity.1.mean.
1527	ADCk.13.glcm.homogeneity.1.range.
1528	ADCk.13.glcm.homogeneity.2.mean.
1529	ADCk.13.glcm.homogeneity.2.range.
1530	ADCk.13.glcm.homogeneity.3.mean.
1531	ADCk.13.glcm.homogeneity.3.range.
1532	ADCk.13.glcm.homogeneity.4.mean.
1533	ADCk.13.glcm.homogeneity.4.range.
1534	ADCk.13.glcm.energy.1.mean.

1535	ADCk.13.glcm.energy.1.range.
1536	ADCk.13.glcm.energy.2.mean.
1537	ADCk.13.glcm.energy.2.range.
1538	ADCk.13.glcm.energy.3.mean.
1539	ADCk.13.glcm.energy.3.range.
1540	ADCk.13.glcm.energy.4.mean.
1541	ADCk.13.glcm.energy.4.range.
1542	ADCk.13.glcm.correlation.1.mean.
1543	ADCk.13.glcm.correlation.1.range.
1544	ADCk.13.glcm.correlation.2.mean.
1545	ADCk.13.glcm.correlation.2.range.
1546	ADCk.13.glcm.correlation.3.mean.
1547	ADCk.13.glcm.correlation.3.range.
1548	ADCk.13.glcm.correlation.4.mean.
1549	ADCk.13.glcm.correlation.4.range.
1550	ADCk.13.glcm.ASM.1.mean.
1551	ADCk.13.glcm.ASM.1.range.
1552	ADCk.13.glcm.ASM.2.mean.
1553	ADCk.13.glcm.ASM.2.range.
1554	ADCk.13.glcm.ASM.3.mean.
1555	ADCk.13.glcm.ASM.3.range.
1556	ADCk.13.glcm.ASM.4.mean.
1557	ADCk.13.glcm.ASM.4.range.
1558	ADCk.15.glcm.contrast.1.mean.
1559	ADCk.15.glcm.contrast.1.range.
1560	ADCk.15.glcm.contrast.2.mean.
1561	ADCk.15.glcm.contrast.2.range.
1562	ADCk.15.glcm.contrast.3.mean.
1563	ADCk.15.glcm.contrast.3.range.
1564	ADCk.15.glcm.contrast.4.mean.
1565	ADCk.15.glcm.contrast.4.range.
1566	ADCk.15.glcm.dissimilarity.1.mean.
1567	ADCk.15.glcm.dissimilarity.1.range.
1568	ADCk.15.glcm.dissimilarity.2.mean.
1569	ADCk.15.glcm.dissimilarity.2.range.
1570	ADCk.15.glcm.dissimilarity.3.mean.
1571	ADCk.15.glcm.dissimilarity.3.range.

1572	ADCk.15.glcm.dissimilarity.4.mean.
1573	ADCk.15.glcm.dissimilarity.4.range.
1574	ADCk.15.glcm.homogeneity.1.mean.
1575	ADCk.15.glcm.homogeneity.1.range.
1576	ADCk.15.glcm.homogeneity.2.mean.
1577	ADCk.15.glcm.homogeneity.2.range.
1578	ADCk.15.glcm.homogeneity.3.mean.
1579	ADCk.15.glcm.homogeneity.3.range.
1580	ADCk.15.glcm.homogeneity.4.mean.
1581	ADCk.15.glcm.homogeneity.4.range.
1582	ADCk.15.glcm.energy.1.mean.
1583	ADCk.15.glcm.energy.1.range.
1584	ADCk.15.glcm.energy.2.mean.
1585	ADCk.15.glcm.energy.2.range.
1586	ADCk.15.glcm.energy.3.mean.
1587	ADCk.15.glcm.energy.3.range.
1588	ADCk.15.glcm.energy.4.mean.
1589	ADCk.15.glcm.energy.4.range.
1590	ADCk.15.glcm.correlation.1.mean.
1591	ADCk.15.glcm.correlation.1.range.
1592	ADCk.15.glcm.correlation.2.mean.
1593	ADCk.15.glcm.correlation.2.range.
1594	ADCk.15.glcm.correlation.3.mean.
1595	ADCk.15.glcm.correlation.3.range.
1596	ADCk.15.glcm.correlation.4.mean.
1597	ADCk.15.glcm.correlation.4.range.
1598	ADCk.15.glcm.ASM.1.mean.
1599	ADCk.15.glcm.ASM.1.range.
1600	ADCk.15.glcm.ASM.2.mean.
1601	ADCk.15.glcm.ASM.2.range.
1602	ADCk.15.glcm.ASM.3.mean.
1603	ADCk.15.glcm.ASM.3.range.
1604	ADCk.15.glcm.ASM.4.mean.
1605	ADCk.15.glcm.ASM.4.range.
1606	ADCk.mbb.glcm.contrast.1.mean.
1607	ADCk.mbb.glcm.contrast.1.range.
1608	ADCk.mbb.glcm.contrast.2.mean.

1609	ADCk.mbb.glcm.contrast.2.range.
1610	ADCk.mbb.glcm.contrast.3.mean.
1611	ADCk.mbb.glcm.contrast.3.range.
1612	ADCk.mbb.glcm.contrast.4.mean.
1613	ADCk.mbb.glcm.contrast.4.range.
1614	ADCk.mbb.glcm.dissimilarity.1.mean.
1615	ADCk.mbb.glcm.dissimilarity.1.range.
1616	ADCk.mbb.glcm.dissimilarity.2.mean.
1617	ADCk.mbb.glcm.dissimilarity.2.range.
1618	ADCk.mbb.glcm.dissimilarity.3.mean.
1619	ADCk.mbb.glcm.dissimilarity.3.range.
1620	ADCk.mbb.glcm.dissimilarity.4.mean.
1621	ADCk.mbb.glcm.dissimilarity.4.range.
1622	ADCk.mbb.glcm.homogeneity.1.mean.
1623	ADCk.mbb.glcm.homogeneity.1.range.
1624	ADCk.mbb.glcm.homogeneity.2.mean.
1625	ADCk.mbb.glcm.homogeneity.2.range.
1626	ADCk.mbb.glcm.homogeneity.3.mean.
1627	ADCk.mbb.glcm.homogeneity.3.range.
1628	ADCk.mbb.glcm.homogeneity.4.mean.
1629	ADCk.mbb.glcm.homogeneity.4.range.
1630	ADCk.mbb.glcm.energy.1.mean.
1631	ADCk.mbb.glcm.energy.1.range.
1632	ADCk.mbb.glcm.energy.2.mean.
1633	ADCk.mbb.glcm.energy.2.range.
1634	ADCk.mbb.glcm.energy.3.mean.
1635	ADCk.mbb.glcm.energy.3.range.
1636	ADCk.mbb.glcm.energy.4.mean.
1637	ADCk.mbb.glcm.energy.4.range.
1638	ADCk.mbb.glcm.correlation.1.mean.
1639	ADCk.mbb.glcm.correlation.1.range.
1640	ADCk.mbb.glcm.correlation.2.mean.

1641	ADCk.mbb.glcm.correlation.2.range.
1642	ADCk.mbb.glcm.correlation.3.mean.
1643	ADCk.mbb.glcm.correlation.3.range.
1644	ADCk.mbb.glcm.correlation.4.mean.
1645	ADCk.mbb.glcm.correlation.4.range.
1646	ADCk.mbb.glcm.ASM.1.mean.
1647	ADCk.mbb.glcm.ASM.1.range.
1648	ADCk.mbb.glcm.ASM.2.mean.
1649	ADCk.mbb.glcm.ASM.2.range.
1650	ADCk.mbb.glcm.ASM.3.mean.
1651	ADCk.mbb.glcm.ASM.3.range.
1652	ADCk.mbb.glcm.ASM.4.mean.
1653	ADCk.mbb.glcm.ASM.4.range.
1654	ADCk.3.lbp.1.0.
1655	ADCk.3.lbp.1.1.
1656	ADCk.3.lbp.1.2.
1657	ADCk.3.lbp.1.3.
1658	ADCk.3.lbp.1.4.
1659	ADCk.3.lbp.1.5.
1660	ADCk.3.lbp.1.6.
1661	ADCk.3.lbp.1.7.
1662	ADCk.3.lbp.1.8.
1663	ADCk.3.lbp.1.9.
1664	ADCk.5.lbp.2.0.
1665	ADCk.5.lbp.2.1.
1666	ADCk.5.lbp.2.2.
1667	ADCk.5.lbp.2.3.
1668	ADCk.5.lbp.2.4.
1669	ADCk.5.lbp.2.5.
1670	ADCk.5.lbp.2.6.
1671	ADCk.5.lbp.2.7.
1672	ADCk.5.lbp.2.8.
1673	ADCk.5.lbp.2.9.
1674	ADCk.7.lbp.3.0.
1675	ADCk.7.lbp.3.1.
1676	ADCk.7.lbp.3.2.
1677	ADCk.7.lbp.3.3.

1678	ADCk.7.lbp.3.4.
1679	ADCk.7.lbp.3.5.
1680	ADCk.7.lbp.3.6.
1681	ADCk.7.lbp.3.7.
1682	ADCk.7.lbp.3.8.
1683	ADCk.7.lbp.3.9.
1684	ADCk.9.lbp.4.0.
1685	ADCk.9.lbp.4.1.
1686	ADCk.9.lbp.4.2.
1687	ADCk.9.lbp.4.3.
1688	ADCk.9.lbp.4.4.
1689	ADCk.9.lbp.4.5.
1690	ADCk.9.lbp.4.6.
1691	ADCk.9.lbp.4.7.
1692	ADCk.9.lbp.4.8.
1693	ADCk.9.lbp.4.9.
1694	ADCk.11.lbp.5.0.
1695	ADCk.11.lbp.5.1.
1696	ADCk.11.lbp.5.2.
1697	ADCk.11.lbp.5.3.
1698	ADCk.11.lbp.5.4.
1699	ADCk.11.lbp.5.5.
1700	ADCk.11.lbp.5.6.
1701	ADCk.11.lbp.5.7.
1702	ADCk.11.lbp.5.8.
1703	ADCk.11.lbp.5.9.
1704	ADCk.13.lbp.6.0.
1705	ADCk.13.lbp.6.1.
1706	ADCk.13.lbp.6.2.
1707	ADCk.13.lbp.6.3.
1708	ADCk.13.lbp.6.4.
1709	ADCk.13.lbp.6.5.
1710	ADCk.13.lbp.6.6.
1711	ADCk.13.lbp.6.7.
1712	ADCk.13.lbp.6.8.
1713	ADCk.13.lbp.6.9.
1714	ADCk.15.lbp.7.0.

1715	ADCk.15.lbp.7.1.
1716	ADCk.15.lbp.7.2.
1717	ADCk.15.lbp.7.3.
1718	ADCk.15.lbp.7.4.
1719	ADCk.15.lbp.7.5.
1720	ADCk.15.lbp.7.6.
1721	ADCk.15.lbp.7.7.
1722	ADCk.15.lbp.7.8.
1723	ADCk.15.lbp.7.9.
1724	ADCk.3.hog
1725	ADCk.5.hog
1726	ADCk.7.hog
1727	ADCk.9.hog
1728	ADCk.11.hog
1729	ADCk.13.hog
1730	ADCk.15.hog
1731	ADCk.3.gabor.1.0.1.mean.
1732	ADCk.3.gabor.1.0.1.var.
1733	ADCk.3.gabor.1.0.1.absmean.
1734	ADCk.3.gabor.1.0.1.mag.
1735	ADCk.3.gabor.1.0.2.mean.
1736	ADCk.3.gabor.1.0.2.var.
1737	ADCk.3.gabor.1.0.2.absmean.
1738	ADCk.3.gabor.1.0.2.mag.
1739	ADCk.3.gabor.1.0.3.mean.
1740	ADCk.3.gabor.1.0.3.var.
1741	ADCk.3.gabor.1.0.3.absmean.
1742	ADCk.3.gabor.1.0.3.mag.
1743	ADCk.3.gabor.1.0.4.mean.
1744	ADCk.3.gabor.1.0.4.var.
1745	ADCk.3.gabor.1.0.4.absmean.
1746	ADCk.3.gabor.1.0.4.mag.
1747	ADCk.3.gabor.1.0.5.mean.
1748	ADCk.3.gabor.1.0.5.var.
1749	ADCk.3.gabor.1.0.5.absmean.
1750	ADCk.3.gabor.1.0.5.mag.
1751	ADCk.3.gabor.2.0.1.mean.

1752	ADCk.3.gabor.2.0.1.var.
1753	ADCk.3.gabor.2.0.1.absmean.
1754	ADCk.3.gabor.2.0.1.mag.
1755	ADCk.3.gabor.2.0.2.mean.
1756	ADCk.3.gabor.2.0.2.var.
1757	ADCk.3.gabor.2.0.2.absmean.
1758	ADCk.3.gabor.2.0.2.mag.
1759	ADCk.3.gabor.2.0.3.mean.
1760	ADCk.3.gabor.2.0.3.var.
1761	ADCk.3.gabor.2.0.3.absmean.
1762	ADCk.3.gabor.2.0.3.mag.
1763	ADCk.3.gabor.2.0.4.mean.
1764	ADCk.3.gabor.2.0.4.var.
1765	ADCk.3.gabor.2.0.4.absmean.
1766	ADCk.3.gabor.2.0.4.mag.
1767	ADCk.3.gabor.2.0.5.mean.
1768	ADCk.3.gabor.2.0.5.var.
1769	ADCk.3.gabor.2.0.5.absmean.
1770	ADCk.3.gabor.2.0.5.mag.
1771	ADCk.3.gabor.3.0.1.mean.
1772	ADCk.3.gabor.3.0.1.var.
1773	ADCk.3.gabor.3.0.1.absmean.
1774	ADCk.3.gabor.3.0.1.mag.
1775	ADCk.3.gabor.3.0.2.mean.
1776	ADCk.3.gabor.3.0.2.var.
1777	ADCk.3.gabor.3.0.2.absmean.
1778	ADCk.3.gabor.3.0.2.mag.
1779	ADCk.3.gabor.3.0.3.mean.
1780	ADCk.3.gabor.3.0.3.var.
1781	ADCk.3.gabor.3.0.3.absmean.
1782	ADCk.3.gabor.3.0.3.mag.
1783	ADCk.3.gabor.3.0.4.mean.
1784	ADCk.3.gabor.3.0.4.var.
1785	ADCk.3.gabor.3.0.4.absmean.
1786	ADCk.3.gabor.3.0.4.mag.
1787	ADCk.3.gabor.3.0.5.mean.
1788	ADCk.3.gabor.3.0.5.var.

1789	ADCk.3.gabor.3.0.5.absmean.
1790	ADCk.3.gabor.3.0.5.mag.
1791	ADCk.5.gabor.1.0.1.mean.
1792	ADCk.5.gabor.1.0.1.var.
1793	ADCk.5.gabor.1.0.1.absmean.
1794	ADCk.5.gabor.1.0.1.mag.
1795	ADCk.5.gabor.1.0.2.mean.
1796	ADCk.5.gabor.1.0.2.var.
1797	ADCk.5.gabor.1.0.2.absmean.
1798	ADCk.5.gabor.1.0.2.mag.
1799	ADCk.5.gabor.1.0.3.mean.
1800	ADCk.5.gabor.1.0.3.var.
1801	ADCk.5.gabor.1.0.3.absmean.
1802	ADCk.5.gabor.1.0.3.mag.
1803	ADCk.5.gabor.1.0.4.mean.
1804	ADCk.5.gabor.1.0.4.var.
1805	ADCk.5.gabor.1.0.4.absmean.
1806	ADCk.5.gabor.1.0.4.mag.
1807	ADCk.5.gabor.1.0.5.mean.
1808	ADCk.5.gabor.1.0.5.var.
1809	ADCk.5.gabor.1.0.5.absmean.
1810	ADCk.5.gabor.1.0.5.mag.
1811	ADCk.5.gabor.2.0.1.mean.
1812	ADCk.5.gabor.2.0.1.var.
1813	ADCk.5.gabor.2.0.1.absmean.
1814	ADCk.5.gabor.2.0.1.mag.
1815	ADCk.5.gabor.2.0.2.mean.
1816	ADCk.5.gabor.2.0.2.var.
1817	ADCk.5.gabor.2.0.2.absmean.
1818	ADCk.5.gabor.2.0.2.mag.
1819	ADCk.5.gabor.2.0.3.mean.
1820	ADCk.5.gabor.2.0.3.var.
1821	ADCk.5.gabor.2.0.3.absmean.
1822	ADCk.5.gabor.2.0.3.mag.
1823	ADCk.5.gabor.2.0.4.mean.
1824	ADCk.5.gabor.2.0.4.var.
1825	ADCk.5.gabor.2.0.4.absmean.

1826	ADCk.5.gabor.2.0.4.mag.
1827	ADCk.5.gabor.2.0.5.mean.
1828	ADCk.5.gabor.2.0.5.var.
1829	ADCk.5.gabor.2.0.5.absmean.
1830	ADCk.5.gabor.2.0.5.mag.
1831	ADCk.5.gabor.3.0.1.mean.
1832	ADCk.5.gabor.3.0.1.var.
1833	ADCk.5.gabor.3.0.1.absmean.
1834	ADCk.5.gabor.3.0.1.mag.
1835	ADCk.5.gabor.3.0.2.mean.
1836	ADCk.5.gabor.3.0.2.var.
1837	ADCk.5.gabor.3.0.2.absmean.
1838	ADCk.5.gabor.3.0.2.mag.
1839	ADCk.5.gabor.3.0.3.mean.
1840	ADCk.5.gabor.3.0.3.var.
1841	ADCk.5.gabor.3.0.3.absmean.
1842	ADCk.5.gabor.3.0.3.mag.
1843	ADCk.5.gabor.3.0.4.mean.
1844	ADCk.5.gabor.3.0.4.var.
1845	ADCk.5.gabor.3.0.4.absmean.
1846	ADCk.5.gabor.3.0.4.mag.
1847	ADCk.5.gabor.3.0.5.mean.
1848	ADCk.5.gabor.3.0.5.var.
1849	ADCk.5.gabor.3.0.5.absmean.
1850	ADCk.5.gabor.3.0.5.mag.
1851	ADCk.7.gabor.1.0.1.mean.
1852	ADCk.7.gabor.1.0.1.var.
1853	ADCk.7.gabor.1.0.1.absmean.
1854	ADCk.7.gabor.1.0.1.mag.
1855	ADCk.7.gabor.1.0.2.mean.
1856	ADCk.7.gabor.1.0.2.var.
1857	ADCk.7.gabor.1.0.2.absmean.
1858	ADCk.7.gabor.1.0.2.mag.
1859	ADCk.7.gabor.1.0.3.mean.
1860	ADCk.7.gabor.1.0.3.var.
1861	ADCk.7.gabor.1.0.3.absmean.
1862	ADCk.7.gabor.1.0.3.mag.

1863	ADCk.7.gabor.1.0.4.mean.
1864	ADCk.7.gabor.1.0.4.var.
1865	ADCk.7.gabor.1.0.4.absmean.
1866	ADCk.7.gabor.1.0.4.mag.
1867	ADCk.7.gabor.1.0.5.mean.
1868	ADCk.7.gabor.1.0.5.var.
1869	ADCk.7.gabor.1.0.5.absmean.
1870	ADCk.7.gabor.1.0.5.mag.
1871	ADCk.7.gabor.2.0.1.mean.
1872	ADCk.7.gabor.2.0.1.var.
1873	ADCk.7.gabor.2.0.1.absmean.
1874	ADCk.7.gabor.2.0.1.mag.
1875	ADCk.7.gabor.2.0.2.mean.
1876	ADCk.7.gabor.2.0.2.var.
1877	ADCk.7.gabor.2.0.2.absmean.
1878	ADCk.7.gabor.2.0.2.mag.
1879	ADCk.7.gabor.2.0.3.mean.
1880	ADCk.7.gabor.2.0.3.var.
1881	ADCk.7.gabor.2.0.3.absmean.
1882	ADCk.7.gabor.2.0.3.mag.
1883	ADCk.7.gabor.2.0.4.mean.
1884	ADCk.7.gabor.2.0.4.var.
1885	ADCk.7.gabor.2.0.4.absmean.
1886	ADCk.7.gabor.2.0.4.mag.
1887	ADCk.7.gabor.2.0.5.mean.
1888	ADCk.7.gabor.2.0.5.var.
1889	ADCk.7.gabor.2.0.5.absmean.
1890	ADCk.7.gabor.2.0.5.mag.
1891	ADCk.7.gabor.3.0.1.mean.
1892	ADCk.7.gabor.3.0.1.var.
1893	ADCk.7.gabor.3.0.1.absmean.
1894	ADCk.7.gabor.3.0.1.mag.
1895	ADCk.7.gabor.3.0.2.mean.
1896	ADCk.7.gabor.3.0.2.var.
1897	ADCk.7.gabor.3.0.2.absmean.
1898	ADCk.7.gabor.3.0.2.mag.
1899	ADCk.7.gabor.3.0.3.mean.

1900	ADCk.7.gabor.3.0.3.var.
1901	ADCk.7.gabor.3.0.3.absmean.
1902	ADCk.7.gabor.3.0.3.mag.
1903	ADCk.7.gabor.3.0.4.mean.
1904	ADCk.7.gabor.3.0.4.var.
1905	ADCk.7.gabor.3.0.4.absmean.
1906	ADCk.7.gabor.3.0.4.mag.
1907	ADCk.7.gabor.3.0.5.mean.
1908	ADCk.7.gabor.3.0.5.var.
1909	ADCk.7.gabor.3.0.5.absmean.
1910	ADCk.7.gabor.3.0.5.mag.
1911	ADCk.9.gabor.1.0.1.mean.
1912	ADCk.9.gabor.1.0.1.var.
1913	ADCk.9.gabor.1.0.1.absmean.
1914	ADCk.9.gabor.1.0.1.mag.
1915	ADCk.9.gabor.1.0.2.mean.
1916	ADCk.9.gabor.1.0.2.var.
1917	ADCk.9.gabor.1.0.2.absmean.
1918	ADCk.9.gabor.1.0.2.mag.
1919	ADCk.9.gabor.1.0.3.mean.
1920	ADCk.9.gabor.1.0.3.var.
1921	ADCk.9.gabor.1.0.3.absmean.
1922	ADCk.9.gabor.1.0.3.mag.
1923	ADCk.9.gabor.1.0.4.mean.
1924	ADCk.9.gabor.1.0.4.var.
1925	ADCk.9.gabor.1.0.4.absmean.
1926	ADCk.9.gabor.1.0.4.mag.
1927	ADCk.9.gabor.1.0.5.mean.
1928	ADCk.9.gabor.1.0.5.var.
1929	ADCk.9.gabor.1.0.5.absmean.
1930	ADCk.9.gabor.1.0.5.mag.
1931	ADCk.9.gabor.2.0.1.mean.
1932	ADCk.9.gabor.2.0.1.var.
1933	ADCk.9.gabor.2.0.1.absmean.
1934	ADCk.9.gabor.2.0.1.mag.
1935	ADCk.9.gabor.2.0.2.mean.
1936	ADCk.9.gabor.2.0.2.var.

1937	ADCk.9.gabor.2.0.2.absmean.
1938	ADCk.9.gabor.2.0.2.mag.
1939	ADCk.9.gabor.2.0.3.mean.
1940	ADCk.9.gabor.2.0.3.var.
1941	ADCk.9.gabor.2.0.3.absmean.
1942	ADCk.9.gabor.2.0.3.mag.
1943	ADCk.9.gabor.2.0.4.mean.
1944	ADCk.9.gabor.2.0.4.var.
1945	ADCk.9.gabor.2.0.4.absmean.
1946	ADCk.9.gabor.2.0.4.mag.
1947	ADCk.9.gabor.2.0.5.mean.
1948	ADCk.9.gabor.2.0.5.var.
1949	ADCk.9.gabor.2.0.5.absmean.
1950	ADCk.9.gabor.2.0.5.mag.
1951	ADCk.9.gabor.3.0.1.mean.
1952	ADCk.9.gabor.3.0.1.var.
1953	ADCk.9.gabor.3.0.1.absmean.
1954	ADCk.9.gabor.3.0.1.mag.
1955	ADCk.9.gabor.3.0.2.mean.
1956	ADCk.9.gabor.3.0.2.var.
1957	ADCk.9.gabor.3.0.2.absmean.
1958	ADCk.9.gabor.3.0.2.mag.
1959	ADCk.9.gabor.3.0.3.mean.
1960	ADCk.9.gabor.3.0.3.var.
1961	ADCk.9.gabor.3.0.3.absmean.
1962	ADCk.9.gabor.3.0.3.mag.
1963	ADCk.9.gabor.3.0.4.mean.
1964	ADCk.9.gabor.3.0.4.var.
1965	ADCk.9.gabor.3.0.4.absmean.
1966	ADCk.9.gabor.3.0.4.mag.
1967	ADCk.9.gabor.3.0.5.mean.
1968	ADCk.9.gabor.3.0.5.var.
1969	ADCk.9.gabor.3.0.5.absmean.
1970	ADCk.9.gabor.3.0.5.mag.
1971	ADCk.11.gabor.1.0.1.mean.
1972	ADCk.11.gabor.1.0.1.var.
1973	ADCk.11.gabor.1.0.1.absmean.

1974	ADCk.11.gabor.1.0.1.mag.
1975	ADCk.11.gabor.1.0.2.mean.
1976	ADCk.11.gabor.1.0.2.var.
1977	ADCk.11.gabor.1.0.2.absmean.
1978	ADCk.11.gabor.1.0.2.mag.
1979	ADCk.11.gabor.1.0.3.mean.
1980	ADCk.11.gabor.1.0.3.var.
1981	ADCk.11.gabor.1.0.3.absmean.
1982	ADCk.11.gabor.1.0.3.mag.
1983	ADCk.11.gabor.1.0.4.mean.
1984	ADCk.11.gabor.1.0.4.var.
1985	ADCk.11.gabor.1.0.4.absmean.
1986	ADCk.11.gabor.1.0.4.mag.
1987	ADCk.11.gabor.1.0.5.mean.
1988	ADCk.11.gabor.1.0.5.var.
1989	ADCk.11.gabor.1.0.5.absmean.
1990	ADCk.11.gabor.1.0.5.mag.
1991	ADCk.11.gabor.2.0.1.mean.
1992	ADCk.11.gabor.2.0.1.var.
1993	ADCk.11.gabor.2.0.1.absmean.
1994	ADCk.11.gabor.2.0.1.mag.
1995	ADCk.11.gabor.2.0.2.mean.
1996	ADCk.11.gabor.2.0.2.var.
1997	ADCk.11.gabor.2.0.2.absmean.
1998	ADCk.11.gabor.2.0.2.mag.
1999	ADCk.11.gabor.2.0.3.mean.
2000	ADCk.11.gabor.2.0.3.var.
2001	ADCk.11.gabor.2.0.3.absmean.
2002	ADCk.11.gabor.2.0.3.mag.
2003	ADCk.11.gabor.2.0.4.mean.
2004	ADCk.11.gabor.2.0.4.var.
2005	ADCk.11.gabor.2.0.4.absmean.
2006	ADCk.11.gabor.2.0.4.mag.
2007	ADCk.11.gabor.2.0.5.mean.
2008	ADCk.11.gabor.2.0.5.var.
2009	ADCk.11.gabor.2.0.5.absmean.
2010	ADCk.11.gabor.2.0.5.mag.

2011	ADCk.11.gabor.3.0.1.mean.
2012	ADCk.11.gabor.3.0.1.var.
2013	ADCk.11.gabor.3.0.1.absmean.
2014	ADCk.11.gabor.3.0.1.mag.
2015	ADCk.11.gabor.3.0.2.mean.
2016	ADCk.11.gabor.3.0.2.var.
2017	ADCk.11.gabor.3.0.2.absmean.
2018	ADCk.11.gabor.3.0.2.mag.
2019	ADCk.11.gabor.3.0.3.mean.
2020	ADCk.11.gabor.3.0.3.var.
2021	ADCk.11.gabor.3.0.3.absmean.
2022	ADCk.11.gabor.3.0.3.mag.
2023	ADCk.11.gabor.3.0.4.mean.
2024	ADCk.11.gabor.3.0.4.var.
2025	ADCk.11.gabor.3.0.4.absmean.
2026	ADCk.11.gabor.3.0.4.mag.
2027	ADCk.11.gabor.3.0.5.mean.
2028	ADCk.11.gabor.3.0.5.var.
2029	ADCk.11.gabor.3.0.5.absmean.
2030	ADCk.11.gabor.3.0.5.mag.
2031	ADCk.13.gabor.1.0.1.mean.
2032	ADCk.13.gabor.1.0.1.var.
2033	ADCk.13.gabor.1.0.1.absmean.
2034	ADCk.13.gabor.1.0.1.mag.
2035	ADCk.13.gabor.1.0.2.mean.
2036	ADCk.13.gabor.1.0.2.var.
2037	ADCk.13.gabor.1.0.2.absmean.
2038	ADCk.13.gabor.1.0.2.mag.
2039	ADCk.13.gabor.1.0.3.mean.
2040	ADCk.13.gabor.1.0.3.var.
2041	ADCk.13.gabor.1.0.3.absmean.
2042	ADCk.13.gabor.1.0.3.mag.
2043	ADCk.13.gabor.1.0.4.mean.
2044	ADCk.13.gabor.1.0.4.var.
2045	ADCk.13.gabor.1.0.4.absmean.
2046	ADCk.13.gabor.1.0.4.mag.
2047	ADCk.13.gabor.1.0.5.mean.

2048	ADCk.13.gabor.1.0.5.var.
2049	ADCk.13.gabor.1.0.5.absmean.
2050	ADCk.13.gabor.1.0.5.mag.
2051	ADCk.13.gabor.2.0.1.mean.
2052	ADCk.13.gabor.2.0.1.var.
2053	ADCk.13.gabor.2.0.1.absmean.
2054	ADCk.13.gabor.2.0.1.mag.
2055	ADCk.13.gabor.2.0.2.mean.
2056	ADCk.13.gabor.2.0.2.var.
2057	ADCk.13.gabor.2.0.2.absmean.
2058	ADCk.13.gabor.2.0.2.mag.
2059	ADCk.13.gabor.2.0.3.mean.
2060	ADCk.13.gabor.2.0.3.var.
2061	ADCk.13.gabor.2.0.3.absmean.
2062	ADCk.13.gabor.2.0.3.mag.
2063	ADCk.13.gabor.2.0.4.mean.
2064	ADCk.13.gabor.2.0.4.var.
2065	ADCk.13.gabor.2.0.4.absmean.
2066	ADCk.13.gabor.2.0.4.mag.
2067	ADCk.13.gabor.2.0.5.mean.
2068	ADCk.13.gabor.2.0.5.var.
2069	ADCk.13.gabor.2.0.5.absmean.
2070	ADCk.13.gabor.2.0.5.mag.
2071	ADCk.13.gabor.3.0.1.mean.
2072	ADCk.13.gabor.3.0.1.var.
2073	ADCk.13.gabor.3.0.1.absmean.
2074	ADCk.13.gabor.3.0.1.mag.
2075	ADCk.13.gabor.3.0.2.mean.
2076	ADCk.13.gabor.3.0.2.var.
2077	ADCk.13.gabor.3.0.2.absmean.
2078	ADCk.13.gabor.3.0.2.mag.
2079	ADCk.13.gabor.3.0.3.mean.
2080	ADCk.13.gabor.3.0.3.var.
2081	ADCk.13.gabor.3.0.3.absmean.
2082	ADCk.13.gabor.3.0.3.mag.
2083	ADCk.13.gabor.3.0.4.mean.
2084	ADCk.13.gabor.3.0.4.var.

2085	ADCk.13.gabor.3.0.4.absmean.
2086	ADCk.13.gabor.3.0.4.mag.
2087	ADCk.13.gabor.3.0.5.mean.
2088	ADCk.13.gabor.3.0.5.var.
2089	ADCk.13.gabor.3.0.5.absmean.
2090	ADCk.13.gabor.3.0.5.mag.
2091	ADCk.15.gabor.1.0.1.mean.
2092	ADCk.15.gabor.1.0.1.var.
2093	ADCk.15.gabor.1.0.1.absmean.
2094	ADCk.15.gabor.1.0.1.mag.
2095	ADCk.15.gabor.1.0.2.mean.
2096	ADCk.15.gabor.1.0.2.var.
2097	ADCk.15.gabor.1.0.2.absmean.
2098	ADCk.15.gabor.1.0.2.mag.
2099	ADCk.15.gabor.1.0.3.mean.
2100	ADCk.15.gabor.1.0.3.var.
2101	ADCk.15.gabor.1.0.3.absmean.
2102	ADCk.15.gabor.1.0.3.mag.
2103	ADCk.15.gabor.1.0.4.mean.
2104	ADCk.15.gabor.1.0.4.var.
2105	ADCk.15.gabor.1.0.4.absmean.
2106	ADCk.15.gabor.1.0.4.mag.
2107	ADCk.15.gabor.1.0.5.mean.
2108	ADCk.15.gabor.1.0.5.var.
2109	ADCk.15.gabor.1.0.5.absmean.
2110	ADCk.15.gabor.1.0.5.mag.
2111	ADCk.15.gabor.2.0.1.mean.
2112	ADCk.15.gabor.2.0.1.var.
2113	ADCk.15.gabor.2.0.1.absmean.
2114	ADCk.15.gabor.2.0.1.mag.
2115	ADCk.15.gabor.2.0.2.mean.
2116	ADCk.15.gabor.2.0.2.var.
2117	ADCk.15.gabor.2.0.2.absmean.
2118	ADCk.15.gabor.2.0.2.mag.
2119	ADCk.15.gabor.2.0.3.mean.
2120	ADCk.15.gabor.2.0.3.var.
2121	ADCk.15.gabor.2.0.3.absmean.

2122	ADCk.15.gabor.2.0.3.mag.
2123	ADCk.15.gabor.2.0.4.mean.
2124	ADCk.15.gabor.2.0.4.var.
2125	ADCk.15.gabor.2.0.4.absmean.
2126	ADCk.15.gabor.2.0.4.mag.
2127	ADCk.15.gabor.2.0.5.mean.
2128	ADCk.15.gabor.2.0.5.var.
2129	ADCk.15.gabor.2.0.5.absmean.
2130	ADCk.15.gabor.2.0.5.mag.
2131	ADCk.15.gabor.3.0.1.mean.
2132	ADCk.15.gabor.3.0.1.var.
2133	ADCk.15.gabor.3.0.1.absmean.
2134	ADCk.15.gabor.3.0.1.mag.
2135	ADCk.15.gabor.3.0.2.mean.
2136	ADCk.15.gabor.3.0.2.var.
2137	ADCk.15.gabor.3.0.2.absmean.
2138	ADCk.15.gabor.3.0.2.mag.
2139	ADCk.15.gabor.3.0.3.mean.
2140	ADCk.15.gabor.3.0.3.var.
2141	ADCk.15.gabor.3.0.3.absmean.
2142	ADCk.15.gabor.3.0.3.mag.
2143	ADCk.15.gabor.3.0.4.mean.
2144	ADCk.15.gabor.3.0.4.var.
2145	ADCk.15.gabor.3.0.4.absmean.
2146	ADCk.15.gabor.3.0.4.mag.
2147	ADCk.15.gabor.3.0.5.mean.
2148	ADCk.15.gabor.3.0.5.var.
2149	ADCk.15.gabor.3.0.5.absmean.
2150	ADCk.15.gabor.3.0.5.mag.
2151	ADCk.3.haar.1.1.aav.
2152	ADCk.3.haar.1.1.std.
2153	ADCk.3.haar.1.2.aav.
2154	ADCk.3.haar.1.2.std.
2155	ADCk.3.haar.1.3.aav.
2156	ADCk.3.haar.1.3.std.
2157	ADCk.3.haar.2.1.aav.
2158	ADCk.3.haar.2.1.std.

2159	ADCk.3.haar.2.2.aav.
2160	ADCk.3.haar.2.2.std.
2161	ADCk.3.haar.2.3.aav.
2162	ADCk.3.haar.2.3.std.
2163	ADCk.3.haar.3.1.aav.
2164	ADCk.3.haar.3.1.std.
2165	ADCk.3.haar.3.2.aav.
2166	ADCk.3.haar.3.2.std.
2167	ADCk.3.haar.3.3.aav.
2168	ADCk.3.haar.3.3.std.
2169	ADCk.3.haar.4.1.aav.
2170	ADCk.3.haar.4.1.std.
2171	ADCk.3.haar.4.2.aav.
2172	ADCk.3.haar.4.2.std.
2173	ADCk.3.haar.4.3.aav.
2174	ADCk.3.haar.4.3.std.
2175	ADCk.5.haar.1.1.aav.
2176	ADCk.5.haar.1.1.std.
2177	ADCk.5.haar.1.2.aav.
2178	ADCk.5.haar.1.2.std.
2179	ADCk.5.haar.1.3.aav.
2180	ADCk.5.haar.1.3.std.
2181	ADCk.5.haar.2.1.aav.
2182	ADCk.5.haar.2.1.std.
2183	ADCk.5.haar.2.2.aav.
2184	ADCk.5.haar.2.2.std.
2185	ADCk.5.haar.2.3.aav.
2186	ADCk.5.haar.2.3.std.
2187	ADCk.5.haar.3.1.aav.
2188	ADCk.5.haar.3.1.std.
2189	ADCk.5.haar.3.2.aav.
2190	ADCk.5.haar.3.2.std.
2191	ADCk.5.haar.3.3.aav.
2192	ADCk.5.haar.3.3.std.
2193	ADCk.5.haar.4.1.aav.
2194	ADCk.5.haar.4.1.std.
2195	ADCk.5.haar.4.2.aav.

2196	ADCk.5.haar.4.2.std.
2197	ADCk.5.haar.4.3.aav.
2198	ADCk.5.haar.4.3.std.
2199	ADCk.7.haar.1.1.aav.
2200	ADCk.7.haar.1.1.std.
2201	ADCk.7.haar.1.2.aav.
2202	ADCk.7.haar.1.2.std.
2203	ADCk.7.haar.1.3.aav.
2204	ADCk.7.haar.1.3.std.
2205	ADCk.7.haar.2.1.aav.
2206	ADCk.7.haar.2.1.std.
2207	ADCk.7.haar.2.2.aav.
2208	ADCk.7.haar.2.2.std.
2209	ADCk.7.haar.2.3.aav.
2210	ADCk.7.haar.2.3.std.
2211	ADCk.7.haar.3.1.aav.
2212	ADCk.7.haar.3.1.std.
2213	ADCk.7.haar.3.2.aav.
2214	ADCk.7.haar.3.2.std.
2215	ADCk.7.haar.3.3.aav.
2216	ADCk.7.haar.3.3.std.
2217	ADCk.7.haar.4.1.aav.
2218	ADCk.7.haar.4.1.std.
2219	ADCk.7.haar.4.2.aav.
2220	ADCk.7.haar.4.2.std.
2221	ADCk.7.haar.4.3.aav.
2222	ADCk.7.haar.4.3.std.
2223	ADCk.9.haar.1.1.aav.
2224	ADCk.9.haar.1.1.std.
2225	ADCk.9.haar.1.2.aav.
2226	ADCk.9.haar.1.2.std.
2227	ADCk.9.haar.1.3.aav.
2228	ADCk.9.haar.1.3.std.
2229	ADCk.9.haar.2.1.aav.
2230	ADCk.9.haar.2.1.std.
2231	ADCk.9.haar.2.2.aav.
2232	ADCk.9.haar.2.2.std.

2233	ADCk.9.haar.2.3.aav.
2234	ADCk.9.haar.2.3.std.
2235	ADCk.9.haar.3.1.aav.
2236	ADCk.9.haar.3.1.std.
2237	ADCk.9.haar.3.2.aav.
2238	ADCk.9.haar.3.2.std.
2239	ADCk.9.haar.3.3.aav.
2240	ADCk.9.haar.3.3.std.
2241	ADCk.9.haar.4.1.aav.
2242	ADCk.9.haar.4.1.std.
2243	ADCk.9.haar.4.2.aav.
2244	ADCk.9.haar.4.2.std.
2245	ADCk.9.haar.4.3.aav.
2246	ADCk.9.haar.4.3.std.
2247	ADCk.11.haar.1.1.aav.
2248	ADCk.11.haar.1.1.std.
2249	ADCk.11.haar.1.2.aav.
2250	ADCk.11.haar.1.2.std.
2251	ADCk.11.haar.1.3.aav.
2252	ADCk.11.haar.1.3.std.
2253	ADCk.11.haar.2.1.aav.
2254	ADCk.11.haar.2.1.std.
2255	ADCk.11.haar.2.2.aav.
2256	ADCk.11.haar.2.2.std.
2257	ADCk.11.haar.2.3.aav.
2258	ADCk.11.haar.2.3.std.
2259	ADCk.11.haar.3.1.aav.
2260	ADCk.11.haar.3.1.std.
2261	ADCk.11.haar.3.2.aav.
2262	ADCk.11.haar.3.2.std.
2263	ADCk.11.haar.3.3.aav.
2264	ADCk.11.haar.3.3.std.
2265	ADCk.11.haar.4.1.aav.
2266	ADCk.11.haar.4.1.std.
2267	ADCk.11.haar.4.2.aav.
2268	ADCk.11.haar.4.2.std.
2269	ADCk.11.haar.4.3.aav.

2270	ADCk.11.haar.4.3.std.
2271	ADCk.13.haar.1.1.aav.
2272	ADCk.13.haar.1.1.std.
2273	ADCk.13.haar.1.2.aav.
2274	ADCk.13.haar.1.2.std.
2275	ADCk.13.haar.1.3.aav.
2276	ADCk.13.haar.1.3.std.
2277	ADCk.13.haar.2.1.aav.
2278	ADCk.13.haar.2.1.std.
2279	ADCk.13.haar.2.2.aav.
2280	ADCk.13.haar.2.2.std.
2281	ADCk.13.haar.2.3.aav.
2282	ADCk.13.haar.2.3.std.
2283	ADCk.13.haar.3.1.aav.
2284	ADCk.13.haar.3.1.std.
2285	ADCk.13.haar.3.2.aav.
2286	ADCk.13.haar.3.2.std.
2287	ADCk.13.haar.3.3.aav.
2288	ADCk.13.haar.3.3.std.
2289	ADCk.13.haar.4.1.aav.
2290	ADCk.13.haar.4.1.std.
2291	ADCk.13.haar.4.2.aav.
2292	ADCk.13.haar.4.2.std.
2293	ADCk.13.haar.4.3.aav.
2294	ADCk.13.haar.4.3.std.
2295	ADCk.15.haar.1.1.aav.
2296	ADCk.15.haar.1.1.std.
2297	ADCk.15.haar.1.2.aav.
2298	ADCk.15.haar.1.2.std.
2299	ADCk.15.haar.1.3.aav.
2300	ADCk.15.haar.1.3.std.
2301	ADCk.15.haar.2.1.aav.
2302	ADCk.15.haar.2.1.std.
2303	ADCk.15.haar.2.2.aav.
2304	ADCk.15.haar.2.2.std.
2305	ADCk.15.haar.2.3.aav.
2306	ADCk.15.haar.2.3.std.

2307	ADCk.15.haar.3.1.aav.
2308	ADCk.15.haar.3.1.std.
2309	ADCk.15.haar.3.2.aav.
2310	ADCk.15.haar.3.2.std.
2311	ADCk.15.haar.3.3.aav.
2312	ADCk.15.haar.3.3.std.
2313	ADCk.15.haar.4.1.aav.
2314	ADCk.15.haar.4.1.std.
2315	ADCk.15.haar.4.2.aav.
2316	ADCk.15.haar.4.2.std.
2317	ADCk.15.haar.4.3.aav.
2318	ADCk.15.haar.4.3.std.
2319	ADCk.3.hu.0.
2320	ADCk.3.hu.1.
2321	ADCk.3.hu.2.
2322	ADCk.3.hu.3.
2323	ADCk.3.hu.4.
2324	ADCk.3.hu.5.
2325	ADCk.3.hu.6.
2326	ADCk.5.hu.0.
2327	ADCk.5.hu.1.
2328	ADCk.5.hu.2.
2329	ADCk.5.hu.3.
2330	ADCk.5.hu.4.
2331	ADCk.5.hu.5.
2332	ADCk.5.hu.6.
2333	ADCk.7.hu.0.
2334	ADCk.7.hu.1.
2335	ADCk.7.hu.2.
2336	ADCk.7.hu.3.
2337	ADCk.7.hu.4.
2338	ADCk.7.hu.5.
2339	ADCk.7.hu.6.
2340	ADCk.9.hu.0.
2341	ADCk.9.hu.1.
2342	ADCk.9.hu.2.
2343	ADCk.9.hu.3.

2344	ADCk.9.hu.4.
2345	ADCk.9.hu.5.
2346	ADCk.9.hu.6.
2347	ADCk.11.hu.0.
2348	ADCk.11.hu.1.
2349	ADCk.11.hu.2.
2350	ADCk.11.hu.3.
2351	ADCk.11.hu.4.
2352	ADCk.11.hu.5.
2353	ADCk.11.hu.6.
2354	ADCk.13.hu.0.
2355	ADCk.13.hu.1.
2356	ADCk.13.hu.2.
2357	ADCk.13.hu.3.
2358	ADCk.13.hu.4.
2359	ADCk.13.hu.5.
2360	ADCk.13.hu.6.
2361	ADCk.15.hu.0.
2362	ADCk.15.hu.1.
2363	ADCk.15.hu.2.
2364	ADCk.15.hu.3.
2365	ADCk.15.hu.4.
2366	ADCk.15.hu.5.
2367	ADCk.15.hu.6.
2368	ADCk.3.zernike.0.
2369	ADCk.3.zernike.1.
2370	ADCk.3.zernike.2.
2371	ADCk.3.zernike.3.
2372	ADCk.3.zernike.4.
2373	ADCk.3.zernike.5.
2374	ADCk.3.zernike.6.
2375	ADCk.3.zernike.7.
2376	ADCk.3.zernike.8.
2377	ADCk.3.zernike.9.
2378	ADCk.3.zernike.10.
2379	ADCk.3.zernike.11.
2380	ADCk.3.zernike.12.

2381	ADCk.3.zernike.13.
2382	ADCk.3.zernike.14.
2383	ADCk.3.zernike.15.
2384	ADCk.3.zernike.16.
2385	ADCk.3.zernike.17.
2386	ADCk.3.zernike.18.
2387	ADCk.3.zernike.19.
2388	ADCk.3.zernike.20.
2389	ADCk.3.zernike.21.
2390	ADCk.3.zernike.22.
2391	ADCk.3.zernike.23.
2392	ADCk.3.zernike.24.
2393	ADCk.5.zernike.0.
2394	ADCk.5.zernike.1.
2395	ADCk.5.zernike.2.
2396	ADCk.5.zernike.3.
2397	ADCk.5.zernike.4.
2398	ADCk.5.zernike.5.
2399	ADCk.5.zernike.6.
2400	ADCk.5.zernike.7.
2401	ADCk.5.zernike.8.
2402	ADCk.5.zernike.9.
2403	ADCk.5.zernike.10.
2404	ADCk.5.zernike.11.
2405	ADCk.5.zernike.12.
2406	ADCk.5.zernike.13.
2407	ADCk.5.zernike.14.
2408	ADCk.5.zernike.15.
2409	ADCk.5.zernike.16.
2410	ADCk.5.zernike.17.
2411	ADCk.5.zernike.18.
2412	ADCk.5.zernike.19.
2413	ADCk.5.zernike.20.
2414	ADCk.5.zernike.21.
2415	ADCk.5.zernike.22.
2416	ADCk.5.zernike.23.
2417	ADCk.5.zernike.24.

2418	ADCk.7.zernike.0.
2419	ADCk.7.zernike.1.
2420	ADCk.7.zernike.2.
2421	ADCk.7.zernike.3.
2422	ADCk.7.zernike.4.
2423	ADCk.7.zernike.5.
2424	ADCk.7.zernike.6.
2425	ADCk.7.zernike.7.
2426	ADCk.7.zernike.8.
2427	ADCk.7.zernike.9.
2428	ADCk.7.zernike.10.
2429	ADCk.7.zernike.11.
2430	ADCk.7.zernike.12.
2431	ADCk.7.zernike.13.
2432	ADCk.7.zernike.14.
2433	ADCk.7.zernike.15.
2434	ADCk.7.zernike.16.
2435	ADCk.7.zernike.17.
2436	ADCk.7.zernike.18.
2437	ADCk.7.zernike.19.
2438	ADCk.7.zernike.20.
2439	ADCk.7.zernike.21.
2440	ADCk.7.zernike.22.
2441	ADCk.7.zernike.23.
2442	ADCk.7.zernike.24.
2443	ADCk.9.zernike.0.
2444	ADCk.9.zernike.1.
2445	ADCk.9.zernike.2.
2446	ADCk.9.zernike.3.
2447	ADCk.9.zernike.4.
2448	ADCk.9.zernike.5.
2449	ADCk.9.zernike.6.
2450	ADCk.9.zernike.7.
2451	ADCk.9.zernike.8.
2452	ADCk.9.zernike.9.
2453	ADCk.9.zernike.10.
2454	ADCk.9.zernike.11.

2455	ADCk.9.zernike.12.
2456	ADCk.9.zernike.13.
2457	ADCk.9.zernike.14.
2458	ADCk.9.zernike.15.
2459	ADCk.9.zernike.16.
2460	ADCk.9.zernike.17.
2461	ADCk.9.zernike.18.
2462	ADCk.9.zernike.19.
2463	ADCk.9.zernike.20.
2464	ADCk.9.zernike.21.
2465	ADCk.9.zernike.22.
2466	ADCk.9.zernike.23.
2467	ADCk.9.zernike.24.
2468	ADCk.11.zernike.0.
2469	ADCk.11.zernike.1.
2470	ADCk.11.zernike.2.
2471	ADCk.11.zernike.3.
2472	ADCk.11.zernike.4.
2473	ADCk.11.zernike.5.
2474	ADCk.11.zernike.6.
2475	ADCk.11.zernike.7.
2476	ADCk.11.zernike.8.
2477	ADCk.11.zernike.9.
2478	ADCk.11.zernike.10.
2479	ADCk.11.zernike.11.
2480	ADCk.11.zernike.12.
2481	ADCk.11.zernike.13.
2482	ADCk.11.zernike.14.
2483	ADCk.11.zernike.15.
2484	ADCk.11.zernike.16.
2485	ADCk.11.zernike.17.
2486	ADCk.11.zernike.18.
2487	ADCk.11.zernike.19.
2488	ADCk.11.zernike.20.
2489	ADCk.11.zernike.21.
2490	ADCk.11.zernike.22.
2491	ADCk.11.zernike.23.

2492	ADCk.11.zernike.24.
2493	ADCk.13.zernike.0.
2494	ADCk.13.zernike.1.
2495	ADCk.13.zernike.2.
2496	ADCk.13.zernike.3.
2497	ADCk.13.zernike.4.
2498	ADCk.13.zernike.5.
2499	ADCk.13.zernike.6.
2500	ADCk.13.zernike.7.
2501	ADCk.13.zernike.8.
2502	ADCk.13.zernike.9.
2503	ADCk.13.zernike.10.
2504	ADCk.13.zernike.11.
2505	ADCk.13.zernike.12.
2506	ADCk.13.zernike.13.
2507	ADCk.13.zernike.14.
2508	ADCk.13.zernike.15.
2509	ADCk.13.zernike.16.
2510	ADCk.13.zernike.17.
2511	ADCk.13.zernike.18.
2512	ADCk.13.zernike.19.
2513	ADCk.13.zernike.20.
2514	ADCk.13.zernike.21.
2515	ADCk.13.zernike.22.
2516	ADCk.13.zernike.23.
2517	ADCk.13.zernike.24.
2518	ADCk.15.zernike.0.
2519	ADCk.15.zernike.1.
2520	ADCk.15.zernike.2.
2521	ADCk.15.zernike.3.
2522	ADCk.15.zernike.4.
2523	ADCk.15.zernike.5.
2524	ADCk.15.zernike.6.
2525	ADCk.15.zernike.7.
2526	ADCk.15.zernike.8.
2527	ADCk.15.zernike.9.
2528	ADCk.15.zernike.10.

2529	ADCk.15.zernike.11.
2530	ADCk.15.zernike.12.
2531	ADCk.15.zernike.13.
2532	ADCk.15.zernike.14.
2533	ADCk.15.zernike.15.
2534	ADCk.15.zernike.16.
2535	ADCk.15.zernike.17.
2536	ADCk.15.zernike.18.
2537	ADCk.15.zernike.19.
2538	ADCk.15.zernike.20.
2539	ADCk.15.zernike.21.
2540	ADCk.15.zernike.22.
2541	ADCk.15.zernike.23.
2542	ADCk.15.zernike.24.
2543	ADCk.3.sobel
2544	ADCk.3.sobel_mask
2545	ADCk.all.stats.p000.
2546	ADCk.all.stats.p010.
2547	ADCk.all.stats.p020.
2548	ADCk.all.stats.p025.
2549	ADCk.all.stats.p030.
2550	ADCk.all.stats.p040.
2551	ADCk.all.stats.p050.
2552	ADCk.all.stats.p060.
2553	ADCk.all.stats.p070.
2554	ADCk.all.stats.p075.
2555	ADCk.all.stats.p080.
2556	ADCk.all.stats.p090.
2557	ADCk.all.stats.p100.
2558	ADCk.all.stats.range.
2559	ADCk.all.stats.mean.
2560	ADCk.all.stats.stddev.
2561	ADCk.all.stats.kurtosis.
2562	ADCk.all.stats.skewness.
2563	K.3.glcm.contrast.1.mean.
2564	K.3.glcm.contrast.1.range.
2565	K.3.glcm.contrast.2.mean.

2566	K.3.glcm.contrast.2.range.
2567	K.3.glcm.contrast.3.mean.
2568	K.3.glcm.contrast.3.range.
2569	K.3.glcm.dissimilarity.1.mean.
2570	K.3.glcm.dissimilarity.1.range.
2571	K.3.glcm.dissimilarity.2.mean.
2572	K.3.glcm.dissimilarity.2.range.
2573	K.3.glcm.dissimilarity.3.mean.
2574	K.3.glcm.dissimilarity.3.range.
2575	K.3.glcm.homogeneity.1.mean.
2576	K.3.glcm.homogeneity.1.range.
2577	K.3.glcm.homogeneity.2.mean.
2578	K.3.glcm.homogeneity.2.range.
2579	K.3.glcm.homogeneity.3.mean.
2580	K.3.glcm.homogeneity.3.range.
2581	K.3.glcm.energy.1.mean.
2582	K.3.glcm.energy.1.range.
2583	K.3.glcm.energy.2.mean.
2584	K.3.glcm.energy.2.range.
2585	K.3.glcm.energy.3.mean.
2586	K.3.glcm.energy.3.range.
2587	K.3.glcm.correlation.1.mean.
2588	K.3.glcm.correlation.1.range.
2589	K.3.glcm.correlation.2.mean.
2590	K.3.glcm.correlation.2.range.
2591	K.3.glcm.correlation.3.mean.
2592	K.3.glcm.correlation.3.range.
2593	K.3.glcm.ASM.1.mean.
2594	K.3.glcm.ASM.1.range.
2595	K.3.glcm.ASM.2.mean.
2596	K.3.glcm.ASM.2.range.
2597	K.3.glcm.ASM.3.mean.
2598	K.3.glcm.ASM.3.range.
2599	K.5.glcm.contrast.1.mean.
2600	K.5.glcm.contrast.1.range.
2601	K.5.glcm.contrast.2.mean.
2602	K.5.glcm.contrast.2.range.

2603	K.5.glcm.contrast.3.mean.
2604	K.5.glcm.contrast.3.range.
2605	K.5.glcm.contrast.4.mean.
2606	K.5.glcm.contrast.4.range.
2607	K.5.glcm.dissimilarity.1.mean.
2608	K.5.glcm.dissimilarity.1.range.
2609	K.5.glcm.dissimilarity.2.mean.
2610	K.5.glcm.dissimilarity.2.range.
2611	K.5.glcm.dissimilarity.3.mean.
2612	K.5.glcm.dissimilarity.3.range.
2613	K.5.glcm.dissimilarity.4.mean.
2614	K.5.glcm.dissimilarity.4.range.
2615	K.5.glcm.homogeneity.1.mean.
2616	K.5.glcm.homogeneity.1.range.
2617	K.5.glcm.homogeneity.2.mean.
2618	K.5.glcm.homogeneity.2.range.
2619	K.5.glcm.homogeneity.3.mean.
2620	K.5.glcm.homogeneity.3.range.
2621	K.5.glcm.homogeneity.4.mean.
2622	K.5.glcm.homogeneity.4.range.
2623	K.5.glcm.energy.1.mean.
2624	K.5.glcm.energy.1.range.
2625	K.5.glcm.energy.2.mean.
2626	K.5.glcm.energy.2.range.
2627	K.5.glcm.energy.3.mean.
2628	K.5.glcm.energy.3.range.
2629	K.5.glcm.energy.4.mean.
2630	K.5.glcm.energy.4.range.
2631	K.5.glcm.correlation.1.mean.
2632	K.5.glcm.correlation.1.range.
2633	K.5.glcm.correlation.2.mean.
2634	K.5.glcm.correlation.2.range.
2635	K.5.glcm.correlation.3.mean.
2636	K.5.glcm.correlation.3.range.
2637	K.5.glcm.correlation.4.mean.
2638	K.5.glcm.correlation.4.range.
2639	K.5.glcm.ASM.1.mean.

2640	K.5.glcm.ASM.1.range.
2641	K.5.glcm.ASM.2.mean.
2642	K.5.glcm.ASM.2.range.
2643	K.5.glcm.ASM.3.mean.
2644	K.5.glcm.ASM.3.range.
2645	K.5.glcm.ASM.4.mean.
2646	K.5.glcm.ASM.4.range.
2647	K.7.glcm.contrast.1.mean.
2648	K.7.glcm.contrast.1.range.
2649	K.7.glcm.contrast.2.mean.
2650	K.7.glcm.contrast.2.range.
2651	K.7.glcm.contrast.3.mean.
2652	K.7.glcm.contrast.3.range.
2653	K.7.glcm.contrast.4.mean.
2654	K.7.glcm.contrast.4.range.
2655	K.7.glcm.dissimilarity.1.mean.
2656	K.7.glcm.dissimilarity.1.range.
2657	K.7.glcm.dissimilarity.2.mean.
2658	K.7.glcm.dissimilarity.2.range.
2659	K.7.glcm.dissimilarity.3.mean.
2660	K.7.glcm.dissimilarity.3.range.
2661	K.7.glcm.dissimilarity.4.mean.
2662	K.7.glcm.dissimilarity.4.range.
2663	K.7.glcm.homogeneity.1.mean.
2664	K.7.glcm.homogeneity.1.range.
2665	K.7.glcm.homogeneity.2.mean.
2666	K.7.glcm.homogeneity.2.range.
2667	K.7.glcm.homogeneity.3.mean.
2668	K.7.glcm.homogeneity.3.range.
2669	K.7.glcm.homogeneity.4.mean.
2670	K.7.glcm.homogeneity.4.range.
2671	K.7.glcm.energy.1.mean.
2672	K.7.glcm.energy.1.range.
2673	K.7.glcm.energy.2.mean.
2674	K.7.glcm.energy.2.range.
2675	K.7.glcm.energy.3.mean.
2676	K.7.glcm.energy.3.range.

2677	K.7.glcm.energy.4.mean.
2678	K.7.glcm.energy.4.range.
2679	K.7.glcm.correlation.1.mean.
2680	K.7.glcm.correlation.1.range.
2681	K.7.glcm.correlation.2.mean.
2682	K.7.glcm.correlation.2.range.
2683	K.7.glcm.correlation.3.mean.
2684	K.7.glcm.correlation.3.range.
2685	K.7.glcm.correlation.4.mean.
2686	K.7.glcm.correlation.4.range.
2687	K.7.glcm.ASM.1.mean.
2688	K.7.glcm.ASM.1.range.
2689	K.7.glcm.ASM.2.mean.
2690	K.7.glcm.ASM.2.range.
2691	K.7.glcm.ASM.3.mean.
2692	K.7.glcm.ASM.3.range.
2693	K.7.glcm.ASM.4.mean.
2694	K.7.glcm.ASM.4.range.
2695	K.9.glcm.contrast.1.mean.
2696	K.9.glcm.contrast.1.range.
2697	K.9.glcm.contrast.2.mean.
2698	K.9.glcm.contrast.2.range.
2699	K.9.glcm.contrast.3.mean.
2700	K.9.glcm.contrast.3.range.
2701	K.9.glcm.contrast.4.mean.
2702	K.9.glcm.contrast.4.range.
2703	K.9.glcm.dissimilarity.1.mean.
2704	K.9.glcm.dissimilarity.1.range.
2705	K.9.glcm.dissimilarity.2.mean.
2706	K.9.glcm.dissimilarity.2.range.
2707	K.9.glcm.dissimilarity.3.mean.
2708	K.9.glcm.dissimilarity.3.range.
2709	K.9.glcm.dissimilarity.4.mean.
2710	K.9.glcm.dissimilarity.4.range.
2711	K.9.glcm.homogeneity.1.mean.
2712	K.9.glcm.homogeneity.1.range.
2713	K.9.glcm.homogeneity.2.mean.

2714	K.9.glcm.homogeneity.2.range.
2715	K.9.glcm.homogeneity.3.mean.
2716	K.9.glcm.homogeneity.3.range.
2717	K.9.glcm.homogeneity.4.mean.
2718	K.9.glcm.homogeneity.4.range.
2719	K.9.glcm.energy.1.mean.
2720	K.9.glcm.energy.1.range.
2721	K.9.glcm.energy.2.mean.
2722	K.9.glcm.energy.2.range.
2723	K.9.glcm.energy.3.mean.
2724	K.9.glcm.energy.3.range.
2725	K.9.glcm.energy.4.mean.
2726	K.9.glcm.energy.4.range.
2727	K.9.glcm.correlation.1.mean.
2728	K.9.glcm.correlation.1.range.
2729	K.9.glcm.correlation.2.mean.
2730	K.9.glcm.correlation.2.range.
2731	K.9.glcm.correlation.3.mean.
2732	K.9.glcm.correlation.3.range.
2733	K.9.glcm.correlation.4.mean.
2734	K.9.glcm.correlation.4.range.
2735	K.9.glcm.ASM.1.mean.
2736	K.9.glcm.ASM.1.range.
2737	K.9.glcm.ASM.2.mean.
2738	K.9.glcm.ASM.2.range.
2739	K.9.glcm.ASM.3.mean.
2740	K.9.glcm.ASM.3.range.
2741	K.9.glcm.ASM.4.mean.
2742	K.9.glcm.ASM.4.range.
2743	K.11.glcm.contrast.1.mean.
2744	K.11.glcm.contrast.1.range.
2745	K.11.glcm.contrast.2.mean.
2746	K.11.glcm.contrast.2.range.
2747	K.11.glcm.contrast.3.mean.
2748	K.11.glcm.contrast.3.range.
2749	K.11.glcm.contrast.4.mean.
2750	K.11.glcm.contrast.4.range.

2751	K.11.glcm.dissimilarity.1.mean.
2752	K.11.glcm.dissimilarity.1.range.
2753	K.11.glcm.dissimilarity.2.mean.
2754	K.11.glcm.dissimilarity.2.range.
2755	K.11.glcm.dissimilarity.3.mean.
2756	K.11.glcm.dissimilarity.3.range.
2757	K.11.glcm.dissimilarity.4.mean.
2758	K.11.glcm.dissimilarity.4.range.
2759	K.11.glcm.homogeneity.1.mean.
2760	K.11.glcm.homogeneity.1.range.
2761	K.11.glcm.homogeneity.2.mean.
2762	K.11.glcm.homogeneity.2.range.
2763	K.11.glcm.homogeneity.3.mean.
2764	K.11.glcm.homogeneity.3.range.
2765	K.11.glcm.homogeneity.4.mean.
2766	K.11.glcm.homogeneity.4.range.
2767	K.11.glcm.energy.1.mean.
2768	K.11.glcm.energy.1.range.
2769	K.11.glcm.energy.2.mean.
2770	K.11.glcm.energy.2.range.
2771	K.11.glcm.energy.3.mean.
2772	K.11.glcm.energy.3.range.
2773	K.11.glcm.energy.4.mean.
2774	K.11.glcm.energy.4.range.
2775	K.11.glcm.correlation.1.mean.
2776	K.11.glcm.correlation.1.range.
2777	K.11.glcm.correlation.2.mean.
2778	K.11.glcm.correlation.2.range.
2779	K.11.glcm.correlation.3.mean.
2780	K.11.glcm.correlation.3.range.
2781	K.11.glcm.correlation.4.mean.
2782	K.11.glcm.correlation.4.range.
2783	K.11.glcm.ASM.1.mean.
2784	K.11.glcm.ASM.1.range.
2785	K.11.glcm.ASM.2.mean.
2786	K.11.glcm.ASM.2.range.
2787	K.11.glcm.ASM.3.mean.

2788	K.11.glcm.ASM.3.range.
2789	K.11.glcm.ASM.4.mean.
2790	K.11.glcm.ASM.4.range.
2791	K.13.glcm.contrast.1.mean.
2792	K.13.glcm.contrast.1.range.
2793	K.13.glcm.contrast.2.mean.
2794	K.13.glcm.contrast.2.range.
2795	K.13.glcm.contrast.3.mean.
2796	K.13.glcm.contrast.3.range.
2797	K.13.glcm.contrast.4.mean.
2798	K.13.glcm.contrast.4.range.
2799	K.13.glcm.dissimilarity.1.mean.
2800	K.13.glcm.dissimilarity.1.range.
2801	K.13.glcm.dissimilarity.2.mean.
2802	K.13.glcm.dissimilarity.2.range.
2803	K.13.glcm.dissimilarity.3.mean.
2804	K.13.glcm.dissimilarity.3.range.
2805	K.13.glcm.dissimilarity.4.mean.
2806	K.13.glcm.dissimilarity.4.range.
2807	K.13.glcm.homogeneity.1.mean.
2808	K.13.glcm.homogeneity.1.range.
2809	K.13.glcm.homogeneity.2.mean.
2810	K.13.glcm.homogeneity.2.range.
2811	K.13.glcm.homogeneity.3.mean.
2812	K.13.glcm.homogeneity.3.range.
2813	K.13.glcm.homogeneity.4.mean.
2814	K.13.glcm.homogeneity.4.range.
2815	K.13.glcm.energy.1.mean.
2816	K.13.glcm.energy.1.range.
2817	K.13.glcm.energy.2.mean.
2818	K.13.glcm.energy.2.range.
2819	K.13.glcm.energy.3.mean.
2820	K.13.glcm.energy.3.range.
2821	K.13.glcm.energy.4.mean.
2822	K.13.glcm.energy.4.range.
2823	K.13.glcm.correlation.1.mean.
2824	K.13.glcm.correlation.1.range.

2825	K.13.glcm.correlation.2.mean.
2826	K.13.glcm.correlation.2.range.
2827	K.13.glcm.correlation.3.mean.
2828	K.13.glcm.correlation.3.range.
2829	K.13.glcm.correlation.4.mean.
2830	K.13.glcm.correlation.4.range.
2831	K.13.glcm.ASM.1.mean.
2832	K.13.glcm.ASM.1.range.
2833	K.13.glcm.ASM.2.mean.
2834	K.13.glcm.ASM.2.range.
2835	K.13.glcm.ASM.3.mean.
2836	K.13.glcm.ASM.3.range.
2837	K.13.glcm.ASM.4.mean.
2838	K.13.glcm.ASM.4.range.
2839	K.15.glcm.contrast.1.mean.
2840	K.15.glcm.contrast.1.range.
2841	K.15.glcm.contrast.2.mean.
2842	K.15.glcm.contrast.2.range.
2843	K.15.glcm.contrast.3.mean.
2844	K.15.glcm.contrast.3.range.
2845	K.15.glcm.contrast.4.mean.
2846	K.15.glcm.contrast.4.range.
2847	K.15.glcm.dissimilarity.1.mean.
2848	K.15.glcm.dissimilarity.1.range.
2849	K.15.glcm.dissimilarity.2.mean.
2850	K.15.glcm.dissimilarity.2.range.
2851	K.15.glcm.dissimilarity.3.mean.
2852	K.15.glcm.dissimilarity.3.range.
2853	K.15.glcm.dissimilarity.4.mean.
2854	K.15.glcm.dissimilarity.4.range.
2855	K.15.glcm.homogeneity.1.mean.
2856	K.15.glcm.homogeneity.1.range.
2857	K.15.glcm.homogeneity.2.mean.
2858	K.15.glcm.homogeneity.2.range.
2859	K.15.glcm.homogeneity.3.mean.
2860	K.15.glcm.homogeneity.3.range.
2861	K.15.glcm.homogeneity.4.mean.

2862	K.15.glcm.homogeneity.4.range.
2863	K.15.glcm.energy.1.mean.
2864	K.15.glcm.energy.1.range.
2865	K.15.glcm.energy.2.mean.
2866	K.15.glcm.energy.2.range.
2867	K.15.glcm.energy.3.mean.
2868	K.15.glcm.energy.3.range.
2869	K.15.glcm.energy.4.mean.
2870	K.15.glcm.energy.4.range.
2871	K.15.glcm.correlation.1.mean.
2872	K.15.glcm.correlation.1.range.
2873	K.15.glcm.correlation.2.mean.
2874	K.15.glcm.correlation.2.range.
2875	K.15.glcm.correlation.3.mean.
2876	K.15.glcm.correlation.3.range.
2877	K.15.glcm.correlation.4.mean.
2878	K.15.glcm.correlation.4.range.
2879	K.15.glcm.ASM.1.mean.
2880	K.15.glcm.ASM.1.range.
2881	K.15.glcm.ASM.2.mean.
2882	K.15.glcm.ASM.2.range.
2883	K.15.glcm.ASM.3.mean.
2884	K.15.glcm.ASM.3.range.
2885	K.15.glcm.ASM.4.mean.
2886	K.15.glcm.ASM.4.range.
2887	K.mbb.glcm.contrast.1.mean.
2888	K.mbb.glcm.contrast.1.range.
2889	K.mbb.glcm.contrast.2.mean.
2890	K.mbb.glcm.contrast.2.range.
2891	K.mbb.glcm.contrast.3.mean.
2892	K.mbb.glcm.contrast.3.range.
2893	K.mbb.glcm.contrast.4.mean.
2894	K.mbb.glcm.contrast.4.range.
2895	K.mbb.glcm.dissimilarity.1.mean.
2896	K.mbb.glcm.dissimilarity.1.range.
2897	K.mbb.glcm.dissimilarity.2.mean.
2898	K.mbb.glcm.dissimilarity.2.range.

2899	K.mbb.glcm.dissimilarity.3.mean.
2900	K.mbb.glcm.dissimilarity.3.range.
2901	K.mbb.glcm.dissimilarity.4.mean.
2902	K.mbb.glcm.dissimilarity.4.range.
2903	K.mbb.glcm.homogeneity.1.mean.
2904	K.mbb.glcm.homogeneity.1.range.
2905	K.mbb.glcm.homogeneity.2.mean.
2906	K.mbb.glcm.homogeneity.2.range.
2907	K.mbb.glcm.homogeneity.3.mean.
2908	K.mbb.glcm.homogeneity.3.range.
2909	K.mbb.glcm.homogeneity.4.mean.
2910	K.mbb.glcm.homogeneity.4.range.
2911	K.mbb.glcm.energy.1.mean.
2912	K.mbb.glcm.energy.1.range.
2913	K.mbb.glcm.energy.2.mean.
2914	K.mbb.glcm.energy.2.range.
2915	K.mbb.glcm.energy.3.mean.
2916	K.mbb.glcm.energy.3.range.
2917	K.mbb.glcm.energy.4.mean.
2918	K.mbb.glcm.energy.4.range.
2919	K.mbb.glcm.correlation.1.mean.
2920	K.mbb.glcm.correlation.1.range.
2921	K.mbb.glcm.correlation.2.mean.
2922	K.mbb.glcm.correlation.2.range.
2923	K.mbb.glcm.correlation.3.mean.
2924	K.mbb.glcm.correlation.3.range.
2925	K.mbb.glcm.correlation.4.mean.
2926	K.mbb.glcm.correlation.4.range.
2927	K.mbb.glcm.ASM.1.mean.
2928	K.mbb.glcm.ASM.1.range.
2929	K.mbb.glcm.ASM.2.mean.
2930	K.mbb.glcm.ASM.2.range.
2931	K.mbb.glcm.ASM.3.mean.
2932	K.mbb.glcm.ASM.3.range.
2933	K.mbb.glcm.ASM.4.mean.
2934	K.mbb.glcm.ASM.4.range.
2935	K.3.lbp.1.0.

2936	K.3.lbp.1.1.
2937	K.3.lbp.1.2.
2938	K.3.lbp.1.3.
2939	K.3.lbp.1.4.
2940	K.3.lbp.1.5.
2941	K.3.lbp.1.6.
2942	K.3.lbp.1.7.
2943	K.3.lbp.1.8.
2944	K.3.lbp.1.9.
2945	K.5.lbp.2.0.
2946	K.5.lbp.2.1.
2947	K.5.lbp.2.2.
2948	K.5.lbp.2.3.
2949	K.5.lbp.2.4.
2950	K.5.lbp.2.5.
2951	K.5.lbp.2.6.
2952	K.5.lbp.2.7.
2953	K.5.lbp.2.8.
2954	K.5.lbp.2.9.
2955	K.7.lbp.3.0.
2956	K.7.lbp.3.1.
2957	K.7.lbp.3.2.
2958	K.7.lbp.3.3.
2959	K.7.lbp.3.4.
2960	K.7.lbp.3.5.
2961	K.7.lbp.3.6.
2962	K.7.lbp.3.7.
2963	K.7.lbp.3.8.
2964	K.7.lbp.3.9.
2965	K.9.lbp.4.0.
2966	K.9.lbp.4.1.
2967	K.9.lbp.4.2.
2968	K.9.lbp.4.3.
2969	K.9.lbp.4.4.
2970	K.9.lbp.4.5.
2971	K.9.lbp.4.6.
2972	K.9.lbp.4.7.

2973	K.9.lbp.4.8.
2974	K.9.lbp.4.9.
2975	K.11.lbp.5.0.
2976	K.11.lbp.5.1.
2977	K.11.lbp.5.2.
2978	K.11.lbp.5.3.
2979	K.11.lbp.5.4.
2980	K.11.lbp.5.5.
2981	K.11.lbp.5.6.
2982	K.11.lbp.5.7.
2983	K.11.lbp.5.8.
2984	K.11.lbp.5.9.
2985	K.13.lbp.6.0.
2986	K.13.lbp.6.1.
2987	K.13.lbp.6.2.
2988	K.13.lbp.6.3.
2989	K.13.lbp.6.4.
2990	K.13.lbp.6.5.
2991	K.13.lbp.6.6.
2992	K.13.lbp.6.7.
2993	K.13.lbp.6.8.
2994	K.13.lbp.6.9.
2995	K.15.lbp.7.0.
2996	K.15.lbp.7.1.
2997	K.15.lbp.7.2.
2998	K.15.lbp.7.3.
2999	K.15.lbp.7.4.
3000	K.15.lbp.7.5.
3001	K.15.lbp.7.6.
3002	K.15.lbp.7.7.
3003	K.15.lbp.7.8.
3004	K.15.lbp.7.9.
3005	K.3.hog
3006	K.5.hog
3007	K.7.hog
3008	K.9.hog
3009	K.11.hog

3010	K.13.hog
3011	K.15.hog
3012	K.3.gabor.1.0.1.mean.
3013	K.3.gabor.1.0.1.var.
3014	K.3.gabor.1.0.1.absmean.
3015	K.3.gabor.1.0.1.mag.
3016	K.3.gabor.1.0.2.mean.
3017	K.3.gabor.1.0.2.var.
3018	K.3.gabor.1.0.2.absmean.
3019	K.3.gabor.1.0.2.mag.
3020	K.3.gabor.1.0.3.mean.
3021	K.3.gabor.1.0.3.var.
3022	K.3.gabor.1.0.3.absmean.
3023	K.3.gabor.1.0.3.mag.
3024	K.3.gabor.1.0.4.mean.
3025	K.3.gabor.1.0.4.var.
3026	K.3.gabor.1.0.4.absmean.
3027	K.3.gabor.1.0.4.mag.
3028	K.3.gabor.1.0.5.mean.
3029	K.3.gabor.1.0.5.var.
3030	K.3.gabor.1.0.5.absmean.
3031	K.3.gabor.1.0.5.mag.
3032	K.3.gabor.2.0.1.mean.
3033	K.3.gabor.2.0.1.var.
3034	K.3.gabor.2.0.1.absmean.
3035	K.3.gabor.2.0.1.mag.
3036	K.3.gabor.2.0.2.mean.
3037	K.3.gabor.2.0.2.var.
3038	K.3.gabor.2.0.2.absmean.
3039	K.3.gabor.2.0.2.mag.
3040	K.3.gabor.2.0.3.mean.
3041	K.3.gabor.2.0.3.var.
3042	K.3.gabor.2.0.3.absmean.
3043	K.3.gabor.2.0.3.mag.
3044	K.3.gabor.2.0.4.mean.
3045	K.3.gabor.2.0.4.var.
3046	K.3.gabor.2.0.4.absmean.

3047	K.3.gabor.2.0.4.mag.
3048	K.3.gabor.2.0.5.mean.
3049	K.3.gabor.2.0.5.var.
3050	K.3.gabor.2.0.5.absmean.
3051	K.3.gabor.2.0.5.mag.
3052	K.3.gabor.3.0.1.mean.
3053	K.3.gabor.3.0.1.var.
3054	K.3.gabor.3.0.1.absmean.
3055	K.3.gabor.3.0.1.mag.
3056	K.3.gabor.3.0.2.mean.
3057	K.3.gabor.3.0.2.var.
3058	K.3.gabor.3.0.2.absmean.
3059	K.3.gabor.3.0.2.mag.
3060	K.3.gabor.3.0.3.mean.
3061	K.3.gabor.3.0.3.var.
3062	K.3.gabor.3.0.3.absmean.
3063	K.3.gabor.3.0.3.mag.
3064	K.3.gabor.3.0.4.mean.
3065	K.3.gabor.3.0.4.var.
3066	K.3.gabor.3.0.4.absmean.
3067	K.3.gabor.3.0.4.mag.
3068	K.3.gabor.3.0.5.mean.
3069	K.3.gabor.3.0.5.var.
3070	K.3.gabor.3.0.5.absmean.
3071	K.3.gabor.3.0.5.mag.
3072	K.5.gabor.1.0.1.mean.
3073	K.5.gabor.1.0.1.var.
3074	K.5.gabor.1.0.1.absmean.
3075	K.5.gabor.1.0.1.mag.
3076	K.5.gabor.1.0.2.mean.
3077	K.5.gabor.1.0.2.var.
3078	K.5.gabor.1.0.2.absmean.
3079	K.5.gabor.1.0.2.mag.
3080	K.5.gabor.1.0.3.mean.
3081	K.5.gabor.1.0.3.var.
3082	K.5.gabor.1.0.3.absmean.
3083	K.5.gabor.1.0.3.mag.

3084	K.5.gabor.1.0.4.mean.
3085	K.5.gabor.1.0.4.var.
3086	K.5.gabor.1.0.4.absmean.
3087	K.5.gabor.1.0.4.mag.
3088	K.5.gabor.1.0.5.mean.
3089	K.5.gabor.1.0.5.var.
3090	K.5.gabor.1.0.5.absmean.
3091	K.5.gabor.1.0.5.mag.
3092	K.5.gabor.2.0.1.mean.
3093	K.5.gabor.2.0.1.var.
3094	K.5.gabor.2.0.1.absmean.
3095	K.5.gabor.2.0.1.mag.
3096	K.5.gabor.2.0.2.mean.
3097	K.5.gabor.2.0.2.var.
3098	K.5.gabor.2.0.2.absmean.
3099	K.5.gabor.2.0.2.mag.
3100	K.5.gabor.2.0.3.mean.
3101	K.5.gabor.2.0.3.var.
3102	K.5.gabor.2.0.3.absmean.
3103	K.5.gabor.2.0.3.mag.
3104	K.5.gabor.2.0.4.mean.
3105	K.5.gabor.2.0.4.var.
3106	K.5.gabor.2.0.4.absmean.
3107	K.5.gabor.2.0.4.mag.
3108	K.5.gabor.2.0.5.mean.
3109	K.5.gabor.2.0.5.var.
3110	K.5.gabor.2.0.5.absmean.
3111	K.5.gabor.2.0.5.mag.
3112	K.5.gabor.3.0.1.mean.
3113	K.5.gabor.3.0.1.var.
3114	K.5.gabor.3.0.1.absmean.
3115	K.5.gabor.3.0.1.mag.
3116	K.5.gabor.3.0.2.mean.
3117	K.5.gabor.3.0.2.var.
3118	K.5.gabor.3.0.2.absmean.
3119	K.5.gabor.3.0.2.mag.
3120	K.5.gabor.3.0.3.mean.

3121	K.5.gabor.3.0.3.var.
3122	K.5.gabor.3.0.3.absmean.
3123	K.5.gabor.3.0.3.mag.
3124	K.5.gabor.3.0.4.mean.
3125	K.5.gabor.3.0.4.var.
3126	K.5.gabor.3.0.4.absmean.
3127	K.5.gabor.3.0.4.mag.
3128	K.5.gabor.3.0.5.mean.
3129	K.5.gabor.3.0.5.var.
3130	K.5.gabor.3.0.5.absmean.
3131	K.5.gabor.3.0.5.mag.
3132	K.7.gabor.1.0.1.mean.
3133	K.7.gabor.1.0.1.var.
3134	K.7.gabor.1.0.1.absmean.
3135	K.7.gabor.1.0.1.mag.
3136	K.7.gabor.1.0.2.mean.
3137	K.7.gabor.1.0.2.var.
3138	K.7.gabor.1.0.2.absmean.
3139	K.7.gabor.1.0.2.mag.
3140	K.7.gabor.1.0.3.mean.
3141	K.7.gabor.1.0.3.var.
3142	K.7.gabor.1.0.3.absmean.
3143	K.7.gabor.1.0.3.mag.
3144	K.7.gabor.1.0.4.mean.
3145	K.7.gabor.1.0.4.var.
3146	K.7.gabor.1.0.4.absmean.
3147	K.7.gabor.1.0.4.mag.
3148	K.7.gabor.1.0.5.mean.
3149	K.7.gabor.1.0.5.var.
3150	K.7.gabor.1.0.5.absmean.
3151	K.7.gabor.1.0.5.mag.
3152	K.7.gabor.2.0.1.mean.
3153	K.7.gabor.2.0.1.var.
3154	K.7.gabor.2.0.1.absmean.
3155	K.7.gabor.2.0.1.mag.
3156	K.7.gabor.2.0.2.mean.
3157	K.7.gabor.2.0.2.var.

3158	K.7.gabor.2.0.2.absmean.
3159	K.7.gabor.2.0.2.mag.
3160	K.7.gabor.2.0.3.mean.
3161	K.7.gabor.2.0.3.var.
3162	K.7.gabor.2.0.3.absmean.
3163	K.7.gabor.2.0.3.mag.
3164	K.7.gabor.2.0.4.mean.
3165	K.7.gabor.2.0.4.var.
3166	K.7.gabor.2.0.4.absmean.
3167	K.7.gabor.2.0.4.mag.
3168	K.7.gabor.2.0.5.mean.
3169	K.7.gabor.2.0.5.var.
3170	K.7.gabor.2.0.5.absmean.
3171	K.7.gabor.2.0.5.mag.
3172	K.7.gabor.3.0.1.mean.
3173	K.7.gabor.3.0.1.var.
3174	K.7.gabor.3.0.1.absmean.
3175	K.7.gabor.3.0.1.mag.
3176	K.7.gabor.3.0.2.mean.
3177	K.7.gabor.3.0.2.var.
3178	K.7.gabor.3.0.2.absmean.
3179	K.7.gabor.3.0.2.mag.
3180	K.7.gabor.3.0.3.mean.
3181	K.7.gabor.3.0.3.var.
3182	K.7.gabor.3.0.3.absmean.
3183	K.7.gabor.3.0.3.mag.
3184	K.7.gabor.3.0.4.mean.
3185	K.7.gabor.3.0.4.var.
3186	K.7.gabor.3.0.4.absmean.
3187	K.7.gabor.3.0.4.mag.
3188	K.7.gabor.3.0.5.mean.
3189	K.7.gabor.3.0.5.var.
3190	K.7.gabor.3.0.5.absmean.
3191	K.7.gabor.3.0.5.mag.
3192	K.9.gabor.1.0.1.mean.
3193	K.9.gabor.1.0.1.var.
3194	K.9.gabor.1.0.1.absmean.

3195	K.9.gabor.1.0.1.mag.
3196	K.9.gabor.1.0.2.mean.
3197	K.9.gabor.1.0.2.var.
3198	K.9.gabor.1.0.2.absmean.
3199	K.9.gabor.1.0.2.mag.
3200	K.9.gabor.1.0.3.mean.
3201	K.9.gabor.1.0.3.var.
3202	K.9.gabor.1.0.3.absmean.
3203	K.9.gabor.1.0.3.mag.
3204	K.9.gabor.1.0.4.mean.
3205	K.9.gabor.1.0.4.var.
3206	K.9.gabor.1.0.4.absmean.
3207	K.9.gabor.1.0.4.mag.
3208	K.9.gabor.1.0.5.mean.
3209	K.9.gabor.1.0.5.var.
3210	K.9.gabor.1.0.5.absmean.
3211	K.9.gabor.1.0.5.mag.
3212	K.9.gabor.2.0.1.mean.
3213	K.9.gabor.2.0.1.var.
3214	K.9.gabor.2.0.1.absmean.
3215	K.9.gabor.2.0.1.mag.
3216	K.9.gabor.2.0.2.mean.
3217	K.9.gabor.2.0.2.var.
3218	K.9.gabor.2.0.2.absmean.
3219	K.9.gabor.2.0.2.mag.
3220	K.9.gabor.2.0.3.mean.
3221	K.9.gabor.2.0.3.var.
3222	K.9.gabor.2.0.3.absmean.
3223	K.9.gabor.2.0.3.mag.
3224	K.9.gabor.2.0.4.mean.
3225	K.9.gabor.2.0.4.var.
3226	K.9.gabor.2.0.4.absmean.
3227	K.9.gabor.2.0.4.mag.
3228	K.9.gabor.2.0.5.mean.
3229	K.9.gabor.2.0.5.var.
3230	K.9.gabor.2.0.5.absmean.
3231	K.9.gabor.2.0.5.mag.

3232	K.9.gabor.3.0.1.mean.
3233	K.9.gabor.3.0.1.var.
3234	K.9.gabor.3.0.1.absmean.
3235	K.9.gabor.3.0.1.mag.
3236	K.9.gabor.3.0.2.mean.
3237	K.9.gabor.3.0.2.var.
3238	K.9.gabor.3.0.2.absmean.
3239	K.9.gabor.3.0.2.mag.
3240	K.9.gabor.3.0.3.mean.
3241	K.9.gabor.3.0.3.var.
3242	K.9.gabor.3.0.3.absmean.
3243	K.9.gabor.3.0.3.mag.
3244	K.9.gabor.3.0.4.mean.
3245	K.9.gabor.3.0.4.var.
3246	K.9.gabor.3.0.4.absmean.
3247	K.9.gabor.3.0.4.mag.
3248	K.9.gabor.3.0.5.mean.
3249	K.9.gabor.3.0.5.var.
3250	K.9.gabor.3.0.5.absmean.
3251	K.9.gabor.3.0.5.mag.
3252	K.11.gabor.1.0.1.mean.
3253	K.11.gabor.1.0.1.var.
3254	K.11.gabor.1.0.1.absmean.
3255	K.11.gabor.1.0.1.mag.
3256	K.11.gabor.1.0.2.mean.
3257	K.11.gabor.1.0.2.var.
3258	K.11.gabor.1.0.2.absmean.
3259	K.11.gabor.1.0.2.mag.
3260	K.11.gabor.1.0.3.mean.
3261	K.11.gabor.1.0.3.var.
3262	K.11.gabor.1.0.3.absmean.
3263	K.11.gabor.1.0.3.mag.
3264	K.11.gabor.1.0.4.mean.
3265	K.11.gabor.1.0.4.var.
3266	K.11.gabor.1.0.4.absmean.
3267	K.11.gabor.1.0.4.mag.
3268	K.11.gabor.1.0.5.mean.

3269	K.11.gabor.1.0.5.var.
3270	K.11.gabor.1.0.5.absmean.
3271	K.11.gabor.1.0.5.mag.
3272	K.11.gabor.2.0.1.mean.
3273	K.11.gabor.2.0.1.var.
3274	K.11.gabor.2.0.1.absmean.
3275	K.11.gabor.2.0.1.mag.
3276	K.11.gabor.2.0.2.mean.
3277	K.11.gabor.2.0.2.var.
3278	K.11.gabor.2.0.2.absmean.
3279	K.11.gabor.2.0.2.mag.
3280	K.11.gabor.2.0.3.mean.
3281	K.11.gabor.2.0.3.var.
3282	K.11.gabor.2.0.3.absmean.
3283	K.11.gabor.2.0.3.mag.
3284	K.11.gabor.2.0.4.mean.
3285	K.11.gabor.2.0.4.var.
3286	K.11.gabor.2.0.4.absmean.
3287	K.11.gabor.2.0.4.mag.
3288	K.11.gabor.2.0.5.mean.
3289	K.11.gabor.2.0.5.var.
3290	K.11.gabor.2.0.5.absmean.
3291	K.11.gabor.2.0.5.mag.
3292	K.11.gabor.3.0.1.mean.
3293	K.11.gabor.3.0.1.var.
3294	K.11.gabor.3.0.1.absmean.
3295	K.11.gabor.3.0.1.mag.
3296	K.11.gabor.3.0.2.mean.
3297	K.11.gabor.3.0.2.var.
3298	K.11.gabor.3.0.2.absmean.
3299	K.11.gabor.3.0.2.mag.
3300	K.11.gabor.3.0.3.mean.
3301	K.11.gabor.3.0.3.var.
3302	K.11.gabor.3.0.3.absmean.
3303	K.11.gabor.3.0.3.mag.
3304	K.11.gabor.3.0.4.mean.
3305	K.11.gabor.3.0.4.var.

3306	K.11.gabor.3.0.4.absmean.
3307	K.11.gabor.3.0.4.mag.
3308	K.11.gabor.3.0.5.mean.
3309	K.11.gabor.3.0.5.var.
3310	K.11.gabor.3.0.5.absmean.
3311	K.11.gabor.3.0.5.mag.
3312	K.13.gabor.1.0.1.mean.
3313	K.13.gabor.1.0.1.var.
3314	K.13.gabor.1.0.1.absmean.
3315	K.13.gabor.1.0.1.mag.
3316	K.13.gabor.1.0.2.mean.
3317	K.13.gabor.1.0.2.var.
3318	K.13.gabor.1.0.2.absmean.
3319	K.13.gabor.1.0.2.mag.
3320	K.13.gabor.1.0.3.mean.
3321	K.13.gabor.1.0.3.var.
3322	K.13.gabor.1.0.3.absmean.
3323	K.13.gabor.1.0.3.mag.
3324	K.13.gabor.1.0.4.mean.
3325	K.13.gabor.1.0.4.var.
3326	K.13.gabor.1.0.4.absmean.
3327	K.13.gabor.1.0.4.mag.
3328	K.13.gabor.1.0.5.mean.
3329	K.13.gabor.1.0.5.var.
3330	K.13.gabor.1.0.5.absmean.
3331	K.13.gabor.1.0.5.mag.
3332	K.13.gabor.2.0.1.mean.
3333	K.13.gabor.2.0.1.var.
3334	K.13.gabor.2.0.1.absmean.
3335	K.13.gabor.2.0.1.mag.
3336	K.13.gabor.2.0.2.mean.
3337	K.13.gabor.2.0.2.var.
3338	K.13.gabor.2.0.2.absmean.
3339	K.13.gabor.2.0.2.mag.
3340	K.13.gabor.2.0.3.mean.
3341	K.13.gabor.2.0.3.var.
3342	K.13.gabor.2.0.3.absmean.

3343	K.13.gabor.2.0.3.mag.
3344	K.13.gabor.2.0.4.mean.
3345	K.13.gabor.2.0.4.var.
3346	K.13.gabor.2.0.4.absmean.
3347	K.13.gabor.2.0.4.mag.
3348	K.13.gabor.2.0.5.mean.
3349	K.13.gabor.2.0.5.var.
3350	K.13.gabor.2.0.5.absmean.
3351	K.13.gabor.2.0.5.mag.
3352	K.13.gabor.3.0.1.mean.
3353	K.13.gabor.3.0.1.var.
3354	K.13.gabor.3.0.1.absmean.
3355	K.13.gabor.3.0.1.mag.
3356	K.13.gabor.3.0.2.mean.
3357	K.13.gabor.3.0.2.var.
3358	K.13.gabor.3.0.2.absmean.
3359	K.13.gabor.3.0.2.mag.
3360	K.13.gabor.3.0.3.mean.
3361	K.13.gabor.3.0.3.var.
3362	K.13.gabor.3.0.3.absmean.
3363	K.13.gabor.3.0.3.mag.
3364	K.13.gabor.3.0.4.mean.
3365	K.13.gabor.3.0.4.var.
3366	K.13.gabor.3.0.4.absmean.
3367	K.13.gabor.3.0.4.mag.
3368	K.13.gabor.3.0.5.mean.
3369	K.13.gabor.3.0.5.var.
3370	K.13.gabor.3.0.5.absmean.
3371	K.13.gabor.3.0.5.mag.
3372	K.15.gabor.1.0.1.mean.
3373	K.15.gabor.1.0.1.var.
3374	K.15.gabor.1.0.1.absmean.
3375	K.15.gabor.1.0.1.mag.
3376	K.15.gabor.1.0.2.mean.
3377	K.15.gabor.1.0.2.var.
3378	K.15.gabor.1.0.2.absmean.
3379	K.15.gabor.1.0.2.mag.

3380	K.15.gabor.1.0.3.mean.
3381	K.15.gabor.1.0.3.var.
3382	K.15.gabor.1.0.3.absmean.
3383	K.15.gabor.1.0.3.mag.
3384	K.15.gabor.1.0.4.mean.
3385	K.15.gabor.1.0.4.var.
3386	K.15.gabor.1.0.4.absmean.
3387	K.15.gabor.1.0.4.mag.
3388	K.15.gabor.1.0.5.mean.
3389	K.15.gabor.1.0.5.var.
3390	K.15.gabor.1.0.5.absmean.
3391	K.15.gabor.1.0.5.mag.
3392	K.15.gabor.2.0.1.mean.
3393	K.15.gabor.2.0.1.var.
3394	K.15.gabor.2.0.1.absmean.
3395	K.15.gabor.2.0.1.mag.
3396	K.15.gabor.2.0.2.mean.
3397	K.15.gabor.2.0.2.var.
3398	K.15.gabor.2.0.2.absmean.
3399	K.15.gabor.2.0.2.mag.
3400	K.15.gabor.2.0.3.mean.
3401	K.15.gabor.2.0.3.var.
3402	K.15.gabor.2.0.3.absmean.
3403	K.15.gabor.2.0.3.mag.
3404	K.15.gabor.2.0.4.mean.
3405	K.15.gabor.2.0.4.var.
3406	K.15.gabor.2.0.4.absmean.
3407	K.15.gabor.2.0.4.mag.
3408	K.15.gabor.2.0.5.mean.
3409	K.15.gabor.2.0.5.var.
3410	K.15.gabor.2.0.5.absmean.
3411	K.15.gabor.2.0.5.mag.
3412	K.15.gabor.3.0.1.mean.
3413	K.15.gabor.3.0.1.var.
3414	K.15.gabor.3.0.1.absmean.
3415	K.15.gabor.3.0.1.mag.
3416	K.15.gabor.3.0.2.mean.

3417	K.15.gabor.3.0.2.var.
3418	K.15.gabor.3.0.2.absmean.
3419	K.15.gabor.3.0.2.mag.
3420	K.15.gabor.3.0.3.mean.
3421	K.15.gabor.3.0.3.var.
3422	K.15.gabor.3.0.3.absmean.
3423	K.15.gabor.3.0.3.mag.
3424	K.15.gabor.3.0.4.mean.
3425	K.15.gabor.3.0.4.var.
3426	K.15.gabor.3.0.4.absmean.
3427	K.15.gabor.3.0.4.mag.
3428	K.15.gabor.3.0.5.mean.
3429	K.15.gabor.3.0.5.var.
3430	K.15.gabor.3.0.5.absmean.
3431	K.15.gabor.3.0.5.mag.
3432	K.3.haar.1.1.aav.
3433	K.3.haar.1.1.std.
3434	K.3.haar.1.2.aav.
3435	K.3.haar.1.2.std.
3436	K.3.haar.1.3.aav.
3437	K.3.haar.1.3.std.
3438	K.3.haar.2.1.aav.
3439	K.3.haar.2.1.std.
3440	K.3.haar.2.2.aav.
3441	K.3.haar.2.2.std.
3442	K.3.haar.2.3.aav.
3443	K.3.haar.2.3.std.
3444	K.3.haar.3.1.aav.
3445	K.3.haar.3.1.std.
3446	K.3.haar.3.2.aav.
3447	K.3.haar.3.2.std.
3448	K.3.haar.3.3.aav.
3449	K.3.haar.3.3.std.
3450	K.3.haar.4.1.aav.
3451	K.3.haar.4.1.std.
3452	K.3.haar.4.2.aav.
3453	K.3.haar.4.2.std.

3454	K.3.haar.4.3.aav.
3455	K.3.haar.4.3.std.
3456	K.5.haar.1.1.aav.
3457	K.5.haar.1.1.std.
3458	K.5.haar.1.2.aav.
3459	K.5.haar.1.2.std.
3460	K.5.haar.1.3.aav.
3461	K.5.haar.1.3.std.
3462	K.5.haar.2.1.aav.
3463	K.5.haar.2.1.std.
3464	K.5.haar.2.2.aav.
3465	K.5.haar.2.2.std.
3466	K.5.haar.2.3.aav.
3467	K.5.haar.2.3.std.
3468	K.5.haar.3.1.aav.
3469	K.5.haar.3.1.std.
3470	K.5.haar.3.2.aav.
3471	K.5.haar.3.2.std.
3472	K.5.haar.3.3.aav.
3473	K.5.haar.3.3.std.
3474	K.5.haar.4.1.aav.
3475	K.5.haar.4.1.std.
3476	K.5.haar.4.2.aav.
3477	K.5.haar.4.2.std.
3478	K.5.haar.4.3.aav.
3479	K.5.haar.4.3.std.
3480	K.7.haar.1.1.aav.
3481	K.7.haar.1.1.std.
3482	K.7.haar.1.2.aav.
3483	K.7.haar.1.2.std.
3484	K.7.haar.1.3.aav.
3485	K.7.haar.1.3.std.
3486	K.7.haar.2.1.aav.
3487	K.7.haar.2.1.std.
3488	K.7.haar.2.2.aav.
3489	K.7.haar.2.2.std.
3490	K.7.haar.2.3.aav.

3491	K.7.haar.2.3.std.
3492	K.7.haar.3.1.aav.
3493	K.7.haar.3.1.std.
3494	K.7.haar.3.2.aav.
3495	K.7.haar.3.2.std.
3496	K.7.haar.3.3.aav.
3497	K.7.haar.3.3.std.
3498	K.7.haar.4.1.aav.
3499	K.7.haar.4.1.std.
3500	K.7.haar.4.2.aav.
3501	K.7.haar.4.2.std.
3502	K.7.haar.4.3.aav.
3503	K.7.haar.4.3.std.
3504	K.9.haar.1.1.aav.
3505	K.9.haar.1.1.std.
3506	K.9.haar.1.2.aav.
3507	K.9.haar.1.2.std.
3508	K.9.haar.1.3.aav.
3509	K.9.haar.1.3.std.
3510	K.9.haar.2.1.aav.
3511	K.9.haar.2.1.std.
3512	K.9.haar.2.2.aav.
3513	K.9.haar.2.2.std.
3514	K.9.haar.2.3.aav.
3515	K.9.haar.2.3.std.
3516	K.9.haar.3.1.aav.
3517	K.9.haar.3.1.std.
3518	K.9.haar.3.2.aav.
3519	K.9.haar.3.2.std.
3520	K.9.haar.3.3.aav.
3521	K.9.haar.3.3.std.
3522	K.9.haar.4.1.aav.
3523	K.9.haar.4.1.std.
3524	K.9.haar.4.2.aav.
3525	K.9.haar.4.2.std.
3526	K.9.haar.4.3.aav.
3527	K.9.haar.4.3.std.

3528	K.11.haar.1.1.aav.
3529	K.11.haar.1.1.std.
3530	K.11.haar.1.2.aav.
3531	K.11.haar.1.2.std.
3532	K.11.haar.1.3.aav.
3533	K.11.haar.1.3.std.
3534	K.11.haar.2.1.aav.
3535	K.11.haar.2.1.std.
3536	K.11.haar.2.2.aav.
3537	K.11.haar.2.2.std.
3538	K.11.haar.2.3.aav.
3539	K.11.haar.2.3.std.
3540	K.11.haar.3.1.aav.
3541	K.11.haar.3.1.std.
3542	K.11.haar.3.2.aav.
3543	K.11.haar.3.2.std.
3544	K.11.haar.3.3.aav.
3545	K.11.haar.3.3.std.
3546	K.11.haar.4.1.aav.
3547	K.11.haar.4.1.std.
3548	K.11.haar.4.2.aav.
3549	K.11.haar.4.2.std.
3550	K.11.haar.4.3.aav.
3551	K.11.haar.4.3.std.
3552	K.13.haar.1.1.aav.
3553	K.13.haar.1.1.std.
3554	K.13.haar.1.2.aav.
3555	K.13.haar.1.2.std.
3556	K.13.haar.1.3.aav.
3557	K.13.haar.1.3.std.
3558	K.13.haar.2.1.aav.
3559	K.13.haar.2.1.std.
3560	K.13.haar.2.2.aav.
3561	K.13.haar.2.2.std.
3562	K.13.haar.2.3.aav.
3563	K.13.haar.2.3.std.
3564	K.13.haar.3.1.aav.

3565	K.13.haar.3.1.std.
3566	K.13.haar.3.2.aav.
3567	K.13.haar.3.2.std.
3568	K.13.haar.3.3.aav.
3569	K.13.haar.3.3.std.
3570	K.13.haar.4.1.aav.
3571	K.13.haar.4.1.std.
3572	K.13.haar.4.2.aav.
3573	K.13.haar.4.2.std.
3574	K.13.haar.4.3.aav.
3575	K.13.haar.4.3.std.
3576	K.15.haar.1.1.aav.
3577	K.15.haar.1.1.std.
3578	K.15.haar.1.2.aav.
3579	K.15.haar.1.2.std.
3580	K.15.haar.1.3.aav.
3581	K.15.haar.1.3.std.
3582	K.15.haar.2.1.aav.
3583	K.15.haar.2.1.std.
3584	K.15.haar.2.2.aav.
3585	K.15.haar.2.2.std.
3586	K.15.haar.2.3.aav.
3587	K.15.haar.2.3.std.
3588	K.15.haar.3.1.aav.
3589	K.15.haar.3.1.std.
3590	K.15.haar.3.2.aav.
3591	K.15.haar.3.2.std.
3592	K.15.haar.3.3.aav.
3593	K.15.haar.3.3.std.
3594	K.15.haar.4.1.aav.
3595	K.15.haar.4.1.std.
3596	K.15.haar.4.2.aav.
3597	K.15.haar.4.2.std.
3598	K.15.haar.4.3.aav.
3599	K.15.haar.4.3.std.
3600	K.3.hu.0.
3601	K.3.hu.1.

3602	K.3.hu.2.
3603	K.3.hu.3.
3604	K.3.hu.4.
3605	K.3.hu.5.
3606	K.3.hu.6.
3607	K.5.hu.0.
3608	K.5.hu.1.
3609	K.5.hu.2.
3610	K.5.hu.3.
3611	K.5.hu.4.
3612	K.5.hu.5.
3613	K.5.hu.6.
3614	K.7.hu.0.
3615	K.7.hu.1.
3616	K.7.hu.2.
3617	K.7.hu.3.
3618	K.7.hu.4.
3619	K.7.hu.5.
3620	K.7.hu.6.
3621	K.9.hu.0.
3622	K.9.hu.1.
3623	K.9.hu.2.
3624	K.9.hu.3.
3625	K.9.hu.4.
3626	K.9.hu.5.
3627	K.9.hu.6.
3628	K.11.hu.0.
3629	K.11.hu.1.
3630	K.11.hu.2.
3631	K.11.hu.3.
3632	K.11.hu.4.
3633	K.11.hu.5.
3634	K.11.hu.6.
3635	K.13.hu.0.
3636	K.13.hu.1.
3637	K.13.hu.2.
3638	K.13.hu.3.

3639	K.13.hu.4.
3640	K.13.hu.5.
3641	K.13.hu.6.
3642	K.15.hu.0.
3643	K.15.hu.1.
3644	K.15.hu.2.
3645	K.15.hu.3.
3646	K.15.hu.4.
3647	K.15.hu.5.
3648	K.15.hu.6.
3649	K.3.zernike.0.
3650	K.3.zernike.1.
3651	K.3.zernike.2.
3652	K.3.zernike.3.
3653	K.3.zernike.4.
3654	K.3.zernike.5.
3655	K.3.zernike.6.
3656	K.3.zernike.7.
3657	K.3.zernike.8.
3658	K.3.zernike.9.
3659	K.3.zernike.10.
3660	K.3.zernike.11.
3661	K.3.zernike.12.
3662	K.3.zernike.13.
3663	K.3.zernike.14.
3664	K.3.zernike.15.
3665	K.3.zernike.16.
3666	K.3.zernike.17.
3667	K.3.zernike.18.
3668	K.3.zernike.19.
3669	K.3.zernike.20.
3670	K.3.zernike.21.
3671	K.3.zernike.22.
3672	K.3.zernike.23.
3673	K.3.zernike.24.
3674	K.5.zernike.0.
3675	K.5.zernike.1.

3676	K.5.zernike.2.
3677	K.5.zernike.3.
3678	K.5.zernike.4.
3679	K.5.zernike.5.
3680	K.5.zernike.6.
3681	K.5.zernike.7.
3682	K.5.zernike.8.
3683	K.5.zernike.9.
3684	K.5.zernike.10.
3685	K.5.zernike.11.
3686	K.5.zernike.12.
3687	K.5.zernike.13.
3688	K.5.zernike.14.
3689	K.5.zernike.15.
3690	K.5.zernike.16.
3691	K.5.zernike.17.
3692	K.5.zernike.18.
3693	K.5.zernike.19.
3694	K.5.zernike.20.
3695	K.5.zernike.21.
3696	K.5.zernike.22.
3697	K.5.zernike.23.
3698	K.5.zernike.24.
3699	K.7.zernike.0.
3700	K.7.zernike.1.
3701	K.7.zernike.2.
3702	K.7.zernike.3.
3703	K.7.zernike.4.
3704	K.7.zernike.5.
3705	K.7.zernike.6.
3706	K.7.zernike.7.
3707	K.7.zernike.8.
3708	K.7.zernike.9.
3709	K.7.zernike.10.
3710	K.7.zernike.11.
3711	K.7.zernike.12.
3712	K.7.zernike.13.

3713	K.7.zernike.14.
3714	K.7.zernike.15.
3715	K.7.zernike.16.
3716	K.7.zernike.17.
3717	K.7.zernike.18.
3718	K.7.zernike.19.
3719	K.7.zernike.20.
3720	K.7.zernike.21.
3721	K.7.zernike.22.
3722	K.7.zernike.23.
3723	K.7.zernike.24.
3724	K.9.zernike.0.
3725	K.9.zernike.1.
3726	K.9.zernike.2.
3727	K.9.zernike.3.
3728	K.9.zernike.4.
3729	K.9.zernike.5.
3730	K.9.zernike.6.
3731	K.9.zernike.7.
3732	K.9.zernike.8.
3733	K.9.zernike.9.
3734	K.9.zernike.10.
3735	K.9.zernike.11.
3736	K.9.zernike.12.
3737	K.9.zernike.13.
3738	K.9.zernike.14.
3739	K.9.zernike.15.
3740	K.9.zernike.16.
3741	K.9.zernike.17.
3742	K.9.zernike.18.
3743	K.9.zernike.19.
3744	K.9.zernike.20.
3745	K.9.zernike.21.
3746	K.9.zernike.22.
3747	K.9.zernike.23.
3748	K.9.zernike.24.
3749	K.11.zernike.0.

3750	K.11.zernike.1.
3751	K.11.zernike.2.
3752	K.11.zernike.3.
3753	K.11.zernike.4.
3754	K.11.zernike.5.
3755	K.11.zernike.6.
3756	K.11.zernike.7.
3757	K.11.zernike.8.
3758	K.11.zernike.9.
3759	K.11.zernike.10.
3760	K.11.zernike.11.
3761	K.11.zernike.12.
3762	K.11.zernike.13.
3763	K.11.zernike.14.
3764	K.11.zernike.15.
3765	K.11.zernike.16.
3766	K.11.zernike.17.
3767	K.11.zernike.18.
3768	K.11.zernike.19.
3769	K.11.zernike.20.
3770	K.11.zernike.21.
3771	K.11.zernike.22.
3772	K.11.zernike.23.
3773	K.11.zernike.24.
3774	K.13.zernike.0.
3775	K.13.zernike.1.
3776	K.13.zernike.2.
3777	K.13.zernike.3.
3778	K.13.zernike.4.
3779	K.13.zernike.5.
3780	K.13.zernike.6.
3781	K.13.zernike.7.
3782	K.13.zernike.8.
3783	K.13.zernike.9.
3784	K.13.zernike.10.
3785	K.13.zernike.11.
3786	K.13.zernike.12.

3787	K.13.zernike.13.
3788	K.13.zernike.14.
3789	K.13.zernike.15.
3790	K.13.zernike.16.
3791	K.13.zernike.17.
3792	K.13.zernike.18.
3793	K.13.zernike.19.
3794	K.13.zernike.20.
3795	K.13.zernike.21.
3796	K.13.zernike.22.
3797	K.13.zernike.23.
3798	K.13.zernike.24.
3799	K.15.zernike.0.
3800	K.15.zernike.1.
3801	K.15.zernike.2.
3802	K.15.zernike.3.
3803	K.15.zernike.4.
3804	K.15.zernike.5.
3805	K.15.zernike.6.
3806	K.15.zernike.7.
3807	K.15.zernike.8.
3808	K.15.zernike.9.
3809	K.15.zernike.10.
3810	K.15.zernike.11.
3811	K.15.zernike.12.
3812	K.15.zernike.13.
3813	K.15.zernike.14.
3814	K.15.zernike.15.
3815	K.15.zernike.16.
3816	K.15.zernike.17.
3817	K.15.zernike.18.
3818	K.15.zernike.19.
3819	K.15.zernike.20.
3820	K.15.zernike.21.
3821	K.15.zernike.22.
3822	K.15.zernike.23.
3823	K.15.zernike.24.

3824	K.3.sobel
3825	K.3.sobel_mask
3826	K.all.stats.p000.
3827	K.all.stats.p010.
3828	K.all.stats.p020.
3829	K.all.stats.p025.
3830	K.all.stats.p030.
3831	K.all.stats.p040.
3832	K.all.stats.p050.
3833	K.all.stats.p060.
3834	K.all.stats.p070.
3835	K.all.stats.p075.
3836	K.all.stats.p080.
3837	K.all.stats.p090.
3838	K.all.stats.p100.
3839	K.all.stats.range.
3840	K.all.stats.mean.
3841	K.all.stats.stddev.
3842	K.all.stats.kurtosis.
3843	K.all.stats.skewness.
3844	T2.3.glcm.contrast.1.mean.
3845	T2.3.glcm.contrast.1.range.
3846	T2.3.glcm.contrast.2.mean.
3847	T2.3.glcm.contrast.2.range.
3848	T2.3.glcm.contrast.3.mean.
3849	T2.3.glcm.contrast.3.range.
3850	T2.3.glcm.dissimilarity.1.mean.
3851	T2.3.glcm.dissimilarity.1.range.
3852	T2.3.glcm.dissimilarity.2.mean.
3853	T2.3.glcm.dissimilarity.2.range.
3854	T2.3.glcm.dissimilarity.3.mean.
3855	T2.3.glcm.dissimilarity.3.range.
3856	T2.3.glcm.homogeneity.1.mean.
3857	T2.3.glcm.homogeneity.1.range.
3858	T2.3.glcm.homogeneity.2.mean.
3859	T2.3.glcm.homogeneity.2.range.
3860	T2.3.glcm.homogeneity.3.mean.

3861	T2.3.glcm.homogeneity.3.range.
3862	T2.3.glcm.energy.1.mean.
3863	T2.3.glcm.energy.1.range.
3864	T2.3.glcm.energy.2.mean.
3865	T2.3.glcm.energy.2.range.
3866	T2.3.glcm.energy.3.mean.
3867	T2.3.glcm.energy.3.range.
3868	T2.3.glcm.correlation.1.mean.
3869	T2.3.glcm.correlation.1.range.
3870	T2.3.glcm.correlation.2.mean.
3871	T2.3.glcm.correlation.2.range.
3872	T2.3.glcm.correlation.3.mean.
3873	T2.3.glcm.correlation.3.range.
3874	T2.3.glcm.ASM.1.mean.
3875	T2.3.glcm.ASM.1.range.
3876	T2.3.glcm.ASM.2.mean.
3877	T2.3.glcm.ASM.2.range.
3878	T2.3.glcm.ASM.3.mean.
3879	T2.3.glcm.ASM.3.range.
3880	T2.7.glcm.contrast.1.mean.
3881	T2.7.glcm.contrast.1.range.
3882	T2.7.glcm.contrast.2.mean.
3883	T2.7.glcm.contrast.2.range.
3884	T2.7.glcm.contrast.3.mean.
3885	T2.7.glcm.contrast.3.range.
3886	T2.7.glcm.contrast.4.mean.
3887	T2.7.glcm.contrast.4.range.
3888	T2.7.glcm.dissimilarity.1.mean.
3889	T2.7.glcm.dissimilarity.1.range.
3890	T2.7.glcm.dissimilarity.2.mean.
3891	T2.7.glcm.dissimilarity.2.range.
3892	T2.7.glcm.dissimilarity.3.mean.
3893	T2.7.glcm.dissimilarity.3.range.
3894	T2.7.glcm.dissimilarity.4.mean.
3895	T2.7.glcm.dissimilarity.4.range.
3896	T2.7.glcm.homogeneity.1.mean.
3897	T2.7.glcm.homogeneity.1.range.

3898	T2.7.glcm.homogeneity.2.mean.
3899	T2.7.glcm.homogeneity.2.range.
3900	T2.7.glcm.homogeneity.3.mean.
3901	T2.7.glcm.homogeneity.3.range.
3902	T2.7.glcm.homogeneity.4.mean.
3903	T2.7.glcm.homogeneity.4.range.
3904	T2.7.glcm.energy.1.mean.
3905	T2.7.glcm.energy.1.range.
3906	T2.7.glcm.energy.2.mean.
3907	T2.7.glcm.energy.2.range.
3908	T2.7.glcm.energy.3.mean.
3909	T2.7.glcm.energy.3.range.
3910	T2.7.glcm.energy.4.mean.
3911	T2.7.glcm.energy.4.range.
3912	T2.7.glcm.correlation.1.mean.
3913	T2.7.glcm.correlation.1.range.
3914	T2.7.glcm.correlation.2.mean.
3915	T2.7.glcm.correlation.2.range.
3916	T2.7.glcm.correlation.3.mean.
3917	T2.7.glcm.correlation.3.range.
3918	T2.7.glcm.correlation.4.mean.
3919	T2.7.glcm.correlation.4.range.
3920	T2.7.glcm.ASM.1.mean.
3921	T2.7.glcm.ASM.1.range.
3922	T2.7.glcm.ASM.2.mean.
3923	T2.7.glcm.ASM.2.range.
3924	T2.7.glcm.ASM.3.mean.
3925	T2.7.glcm.ASM.3.range.
3926	T2.7.glcm.ASM.4.mean.
3927	T2.7.glcm.ASM.4.range.
3928	T2.11.glcm.contrast.1.mean.
3929	T2.11.glcm.contrast.1.range.
3930	T2.11.glcm.contrast.2.mean.
3931	T2.11.glcm.contrast.2.range.
3932	T2.11.glcm.contrast.3.mean.
3933	T2.11.glcm.contrast.3.range.
3934	T2.11.glcm.contrast.4.mean.

3935	T2.11.glcm.contrast.4.range.
3936	T2.11.glcm.dissimilarity.1.mean.
3937	T2.11.glcm.dissimilarity.1.range.
3938	T2.11.glcm.dissimilarity.2.mean.
3939	T2.11.glcm.dissimilarity.2.range.
3940	T2.11.glcm.dissimilarity.3.mean.
3941	T2.11.glcm.dissimilarity.3.range.
3942	T2.11.glcm.dissimilarity.4.mean.
3943	T2.11.glcm.dissimilarity.4.range.
3944	T2.11.glcm.homogeneity.1.mean.
3945	T2.11.glcm.homogeneity.1.range.
3946	T2.11.glcm.homogeneity.2.mean.
3947	T2.11.glcm.homogeneity.2.range.
3948	T2.11.glcm.homogeneity.3.mean.
3949	T2.11.glcm.homogeneity.3.range.
3950	T2.11.glcm.homogeneity.4.mean.
3951	T2.11.glcm.homogeneity.4.range.
3952	T2.11.glcm.energy.1.mean.
3953	T2.11.glcm.energy.1.range.
3954	T2.11.glcm.energy.2.mean.
3955	T2.11.glcm.energy.2.range.
3956	T2.11.glcm.energy.3.mean.
3957	T2.11.glcm.energy.3.range.
3958	T2.11.glcm.energy.4.mean.
3959	T2.11.glcm.energy.4.range.
3960	T2.11.glcm.correlation.1.mean.
3961	T2.11.glcm.correlation.1.range.
3962	T2.11.glcm.correlation.2.mean.
3963	T2.11.glcm.correlation.2.range.
3964	T2.11.glcm.correlation.3.mean.
3965	T2.11.glcm.correlation.3.range.
3966	T2.11.glcm.correlation.4.mean.
3967	T2.11.glcm.correlation.4.range.
3968	T2.11.glcm.ASM.1.mean.
3969	T2.11.glcm.ASM.1.range.
3970	T2.11.glcm.ASM.2.mean.
3971	T2.11.glcm.ASM.2.range.

3972	T2.11.glcm.ASM.3.mean.
3973	T2.11.glcm.ASM.3.range.
3974	T2.11.glcm.ASM.4.mean.
3975	T2.11.glcm.ASM.4.range.
3976	T2.15.glcm.contrast.1.mean.
3977	T2.15.glcm.contrast.1.range.
3978	T2.15.glcm.contrast.2.mean.
3979	T2.15.glcm.contrast.2.range.
3980	T2.15.glcm.contrast.3.mean.
3981	T2.15.glcm.contrast.3.range.
3982	T2.15.glcm.contrast.4.mean.
3983	T2.15.glcm.contrast.4.range.
3984	T2.15.glcm.dissimilarity.1.mean.
3985	T2.15.glcm.dissimilarity.1.range.
3986	T2.15.glcm.dissimilarity.2.mean.
3987	T2.15.glcm.dissimilarity.2.range.
3988	T2.15.glcm.dissimilarity.3.mean.
3989	T2.15.glcm.dissimilarity.3.range.
3990	T2.15.glcm.dissimilarity.4.mean.
3991	T2.15.glcm.dissimilarity.4.range.
3992	T2.15.glcm.homogeneity.1.mean.
3993	T2.15.glcm.homogeneity.1.range.
3994	T2.15.glcm.homogeneity.2.mean.
3995	T2.15.glcm.homogeneity.2.range.
3996	T2.15.glcm.homogeneity.3.mean.
3997	T2.15.glcm.homogeneity.3.range.
3998	T2.15.glcm.homogeneity.4.mean.
3999	T2.15.glcm.homogeneity.4.range.
4000	T2.15.glcm.energy.1.mean.
4001	T2.15.glcm.energy.1.range.
4002	T2.15.glcm.energy.2.mean.
4003	T2.15.glcm.energy.2.range.
4004	T2.15.glcm.energy.3.mean.
4005	T2.15.glcm.energy.3.range.
4006	T2.15.glcm.energy.4.mean.
4007	T2.15.glcm.energy.4.range.
4008	T2.15.glcm.correlation.1.mean.

4009	T2.15.glcm.correlation.1.range.
4010	T2.15.glcm.correlation.2.mean.
4011	T2.15.glcm.correlation.2.range.
4012	T2.15.glcm.correlation.3.mean.
4013	T2.15.glcm.correlation.3.range.
4014	T2.15.glcm.correlation.4.mean.
4015	T2.15.glcm.correlation.4.range.
4016	T2.15.glcm.ASM.1.mean.
4017	T2.15.glcm.ASM.1.range.
4018	T2.15.glcm.ASM.2.mean.
4019	T2.15.glcm.ASM.2.range.
4020	T2.15.glcm.ASM.3.mean.
4021	T2.15.glcm.ASM.3.range.
4022	T2.15.glcm.ASM.4.mean.
4023	T2.15.glcm.ASM.4.range.
4024	T2.19.glcm.contrast.1.mean.
4025	T2.19.glcm.contrast.1.range.
4026	T2.19.glcm.contrast.2.mean.
4027	T2.19.glcm.contrast.2.range.
4028	T2.19.glcm.contrast.3.mean.
4029	T2.19.glcm.contrast.3.range.
4030	T2.19.glcm.contrast.4.mean.
4031	T2.19.glcm.contrast.4.range.
4032	T2.19.glcm.dissimilarity.1.mean.
4033	T2.19.glcm.dissimilarity.1.range.
4034	T2.19.glcm.dissimilarity.2.mean.
4035	T2.19.glcm.dissimilarity.2.range.
4036	T2.19.glcm.dissimilarity.3.mean.
4037	T2.19.glcm.dissimilarity.3.range.
4038	T2.19.glcm.dissimilarity.4.mean.
4039	T2.19.glcm.dissimilarity.4.range.
4040	T2.19.glcm.homogeneity.1.mean.
4041	T2.19.glcm.homogeneity.1.range.
4042	T2.19.glcm.homogeneity.2.mean.
4043	T2.19.glcm.homogeneity.2.range.
4044	T2.19.glcm.homogeneity.3.mean.
4045	T2.19.glcm.homogeneity.3.range.

4046	T2.19.glcm.homogeneity.4.mean.
4047	T2.19.glcm.homogeneity.4.range.
4048	T2.19.glcm.energy.1.mean.
4049	T2.19.glcm.energy.1.range.
4050	T2.19.glcm.energy.2.mean.
4051	T2.19.glcm.energy.2.range.
4052	T2.19.glcm.energy.3.mean.
4053	T2.19.glcm.energy.3.range.
4054	T2.19.glcm.energy.4.mean.
4055	T2.19.glcm.energy.4.range.
4056	T2.19.glcm.correlation.1.mean.
4057	T2.19.glcm.correlation.1.range.
4058	T2.19.glcm.correlation.2.mean.
4059	T2.19.glcm.correlation.2.range.
4060	T2.19.glcm.correlation.3.mean.
4061	T2.19.glcm.correlation.3.range.
4062	T2.19.glcm.correlation.4.mean.
4063	T2.19.glcm.correlation.4.range.
4064	T2.19.glcm.ASM.1.mean.
4065	T2.19.glcm.ASM.1.range.
4066	T2.19.glcm.ASM.2.mean.
4067	T2.19.glcm.ASM.2.range.
4068	T2.19.glcm.ASM.3.mean.
4069	T2.19.glcm.ASM.3.range.
4070	T2.19.glcm.ASM.4.mean.
4071	T2.19.glcm.ASM.4.range.
4072	T2.23.glcm.contrast.1.mean.
4073	T2.23.glcm.contrast.1.range.
4074	T2.23.glcm.contrast.2.mean.
4075	T2.23.glcm.contrast.2.range.
4076	T2.23.glcm.contrast.3.mean.
4077	T2.23.glcm.contrast.3.range.
4078	T2.23.glcm.contrast.4.mean.
4079	T2.23.glcm.contrast.4.range.
4080	T2.23.glcm.dissimilarity.1.mean.
4081	T2.23.glcm.dissimilarity.1.range.
4082	T2.23.glcm.dissimilarity.2.mean.

4083	T2.23.glcm.dissimilarity.2.range.
4084	T2.23.glcm.dissimilarity.3.mean.
4085	T2.23.glcm.dissimilarity.3.range.
4086	T2.23.glcm.dissimilarity.4.mean.
4087	T2.23.glcm.dissimilarity.4.range.
4088	T2.23.glcm.homogeneity.1.mean.
4089	T2.23.glcm.homogeneity.1.range.
4090	T2.23.glcm.homogeneity.2.mean.
4091	T2.23.glcm.homogeneity.2.range.
4092	T2.23.glcm.homogeneity.3.mean.
4093	T2.23.glcm.homogeneity.3.range.
4094	T2.23.glcm.homogeneity.4.mean.
4095	T2.23.glcm.homogeneity.4.range.
4096	T2.23.glcm.energy.1.mean.
4097	T2.23.glcm.energy.1.range.
4098	T2.23.glcm.energy.2.mean.
4099	T2.23.glcm.energy.2.range.
4100	T2.23.glcm.energy.3.mean.
4101	T2.23.glcm.energy.3.range.
4102	T2.23.glcm.energy.4.mean.
4103	T2.23.glcm.energy.4.range.
4104	T2.23.glcm.correlation.1.mean.
4105	T2.23.glcm.correlation.1.range.
4106	T2.23.glcm.correlation.2.mean.
4107	T2.23.glcm.correlation.2.range.
4108	T2.23.glcm.correlation.3.mean.
4109	T2.23.glcm.correlation.3.range.
4110	T2.23.glcm.correlation.4.mean.
4111	T2.23.glcm.correlation.4.range.
4112	T2.23.glcm.ASM.1.mean.
4113	T2.23.glcm.ASM.1.range.
4114	T2.23.glcm.ASM.2.mean.
4115	T2.23.glcm.ASM.2.range.
4116	T2.23.glcm.ASM.3.mean.
4117	T2.23.glcm.ASM.3.range.
4118	T2.23.glcm.ASM.4.mean.
4119	T2.23.glcm.ASM.4.range.

4120	T2.27.glcm.contrast.1.mean.
4121	T2.27.glcm.contrast.1.range.
4122	T2.27.glcm.contrast.2.mean.
4123	T2.27.glcm.contrast.2.range.
4124	T2.27.glcm.contrast.3.mean.
4125	T2.27.glcm.contrast.3.range.
4126	T2.27.glcm.contrast.4.mean.
4127	T2.27.glcm.contrast.4.range.
4128	T2.27.glcm.dissimilarity.1.mean.
4129	T2.27.glcm.dissimilarity.1.range.
4130	T2.27.glcm.dissimilarity.2.mean.
4131	T2.27.glcm.dissimilarity.2.range.
4132	T2.27.glcm.dissimilarity.3.mean.
4133	T2.27.glcm.dissimilarity.3.range.
4134	T2.27.glcm.dissimilarity.4.mean.
4135	T2.27.glcm.dissimilarity.4.range.
4136	T2.27.glcm.homogeneity.1.mean.
4137	T2.27.glcm.homogeneity.1.range.
4138	T2.27.glcm.homogeneity.2.mean.
4139	T2.27.glcm.homogeneity.2.range.
4140	T2.27.glcm.homogeneity.3.mean.
4141	T2.27.glcm.homogeneity.3.range.
4142	T2.27.glcm.homogeneity.4.mean.
4143	T2.27.glcm.homogeneity.4.range.
4144	T2.27.glcm.energy.1.mean.
4145	T2.27.glcm.energy.1.range.
4146	T2.27.glcm.energy.2.mean.
4147	T2.27.glcm.energy.2.range.
4148	T2.27.glcm.energy.3.mean.
4149	T2.27.glcm.energy.3.range.
4150	T2.27.glcm.energy.4.mean.
4151	T2.27.glcm.energy.4.range.
4152	T2.27.glcm.correlation.1.mean.
4153	T2.27.glcm.correlation.1.range.
4154	T2.27.glcm.correlation.2.mean.
4155	T2.27.glcm.correlation.2.range.
4156	T2.27.glcm.correlation.3.mean.

4157	T2.27.glcm.correlation.3.range.
4158	T2.27.glcm.correlation.4.mean.
4159	T2.27.glcm.correlation.4.range.
4160	T2.27.glcm.ASM.1.mean.
4161	T2.27.glcm.ASM.1.range.
4162	T2.27.glcm.ASM.2.mean.
4163	T2.27.glcm.ASM.2.range.
4164	T2.27.glcm.ASM.3.mean.
4165	T2.27.glcm.ASM.3.range.
4166	T2.27.glcm.ASM.4.mean.
4167	T2.27.glcm.ASM.4.range.
4168	T2.31.glcm.contrast.1.mean.
4169	T2.31.glcm.contrast.1.range.
4170	T2.31.glcm.contrast.2.mean.
4171	T2.31.glcm.contrast.2.range.
4172	T2.31.glcm.contrast.3.mean.
4173	T2.31.glcm.contrast.3.range.
4174	T2.31.glcm.contrast.4.mean.
4175	T2.31.glcm.contrast.4.range.
4176	T2.31.glcm.dissimilarity.1.mean.
4177	T2.31.glcm.dissimilarity.1.range.
4178	T2.31.glcm.dissimilarity.2.mean.
4179	T2.31.glcm.dissimilarity.2.range.
4180	T2.31.glcm.dissimilarity.3.mean.
4181	T2.31.glcm.dissimilarity.3.range.
4182	T2.31.glcm.dissimilarity.4.mean.
4183	T2.31.glcm.dissimilarity.4.range.
4184	T2.31.glcm.homogeneity.1.mean.
4185	T2.31.glcm.homogeneity.1.range.
4186	T2.31.glcm.homogeneity.2.mean.
4187	T2.31.glcm.homogeneity.2.range.
4188	T2.31.glcm.homogeneity.3.mean.
4189	T2.31.glcm.homogeneity.3.range.
4190	T2.31.glcm.homogeneity.4.mean.
4191	T2.31.glcm.homogeneity.4.range.
4192	T2.31.glcm.energy.1.mean.
4193	T2.31.glcm.energy.1.range.

4194	T2.31.glcm.energy.2.mean.
4195	T2.31.glcm.energy.2.range.
4196	T2.31.glcm.energy.3.mean.
4197	T2.31.glcm.energy.3.range.
4198	T2.31.glcm.energy.4.mean.
4199	T2.31.glcm.energy.4.range.
4200	T2.31.glcm.correlation.1.mean.
4201	T2.31.glcm.correlation.1.range.
4202	T2.31.glcm.correlation.2.mean.
4203	T2.31.glcm.correlation.2.range.
4204	T2.31.glcm.correlation.3.mean.
4205	T2.31.glcm.correlation.3.range.
4206	T2.31.glcm.correlation.4.mean.
4207	T2.31.glcm.correlation.4.range.
4208	T2.31.glcm.ASM.1.mean.
4209	T2.31.glcm.ASM.1.range.
4210	T2.31.glcm.ASM.2.mean.
4211	T2.31.glcm.ASM.2.range.
4212	T2.31.glcm.ASM.3.mean.
4213	T2.31.glcm.ASM.3.range.
4214	T2.31.glcm.ASM.4.mean.
4215	T2.31.glcm.ASM.4.range.
4216	T2.35.glcm.contrast.1.mean.
4217	T2.35.glcm.contrast.1.range.
4218	T2.35.glcm.contrast.2.mean.
4219	T2.35.glcm.contrast.2.range.
4220	T2.35.glcm.contrast.3.mean.
4221	T2.35.glcm.contrast.3.range.
4222	T2.35.glcm.contrast.4.mean.
4223	T2.35.glcm.contrast.4.range.
4224	T2.35.glcm.dissimilarity.1.mean.
4225	T2.35.glcm.dissimilarity.1.range.
4226	T2.35.glcm.dissimilarity.2.mean.
4227	T2.35.glcm.dissimilarity.2.range.
4228	T2.35.glcm.dissimilarity.3.mean.
4229	T2.35.glcm.dissimilarity.3.range.
4230	T2.35.glcm.dissimilarity.4.mean.

4231	T2.35.glcm.dissimilarity.4.range.
4232	T2.35.glcm.homogeneity.1.mean.
4233	T2.35.glcm.homogeneity.1.range.
4234	T2.35.glcm.homogeneity.2.mean.
4235	T2.35.glcm.homogeneity.2.range.
4236	T2.35.glcm.homogeneity.3.mean.
4237	T2.35.glcm.homogeneity.3.range.
4238	T2.35.glcm.homogeneity.4.mean.
4239	T2.35.glcm.homogeneity.4.range.
4240	T2.35.glcm.energy.1.mean.
4241	T2.35.glcm.energy.1.range.
4242	T2.35.glcm.energy.2.mean.
4243	T2.35.glcm.energy.2.range.
4244	T2.35.glcm.energy.3.mean.
4245	T2.35.glcm.energy.3.range.
4246	T2.35.glcm.energy.4.mean.
4247	T2.35.glcm.energy.4.range.
4248	T2.35.glcm.correlation.1.mean.
4249	T2.35.glcm.correlation.1.range.
4250	T2.35.glcm.correlation.2.mean.
4251	T2.35.glcm.correlation.2.range.
4252	T2.35.glcm.correlation.3.mean.
4253	T2.35.glcm.correlation.3.range.
4254	T2.35.glcm.correlation.4.mean.
4255	T2.35.glcm.correlation.4.range.
4256	T2.35.glcm.ASM.1.mean.
4257	T2.35.glcm.ASM.1.range.
4258	T2.35.glcm.ASM.2.mean.
4259	T2.35.glcm.ASM.2.range.
4260	T2.35.glcm.ASM.3.mean.
4261	T2.35.glcm.ASM.3.range.
4262	T2.35.glcm.ASM.4.mean.
4263	T2.35.glcm.ASM.4.range.
4264	T2.mbb.glcm.contrast.1.mean.
4265	T2.mbb.glcm.contrast.1.range.
4266	T2.mbb.glcm.contrast.2.mean.
4267	T2.mbb.glcm.contrast.2.range.

4268	T2.mbb.glcm.contrast.3.mean.
4269	T2.mbb.glcm.contrast.3.range.
4270	T2.mbb.glcm.contrast.4.mean.
4271	T2.mbb.glcm.contrast.4.range.
4272	T2.mbb.glcm.dissimilarity.1.mean.
4273	T2.mbb.glcm.dissimilarity.1.range.
4274	T2.mbb.glcm.dissimilarity.2.mean.
4275	T2.mbb.glcm.dissimilarity.2.range.
4276	T2.mbb.glcm.dissimilarity.3.mean.
4277	T2.mbb.glcm.dissimilarity.3.range.
4278	T2.mbb.glcm.dissimilarity.4.mean.
4279	T2.mbb.glcm.dissimilarity.4.range.
4280	T2.mbb.glcm.homogeneity.1.mean.
4281	T2.mbb.glcm.homogeneity.1.range.
4282	T2.mbb.glcm.homogeneity.2.mean.
4283	T2.mbb.glcm.homogeneity.2.range.
4284	T2.mbb.glcm.homogeneity.3.mean.
4285	T2.mbb.glcm.homogeneity.3.range.
4286	T2.mbb.glcm.homogeneity.4.mean.
4287	T2.mbb.glcm.homogeneity.4.range.
4288	T2.mbb.glcm.energy.1.mean.
4289	T2.mbb.glcm.energy.1.range.
4290	T2.mbb.glcm.energy.2.mean.
4291	T2.mbb.glcm.energy.2.range.
4292	T2.mbb.glcm.energy.3.mean.
4293	T2.mbb.glcm.energy.3.range.
4294	T2.mbb.glcm.energy.4.mean.
4295	T2.mbb.glcm.energy.4.range.
4296	T2.mbb.glcm.correlation.1.mean.
4297	T2.mbb.glcm.correlation.1.range.
4298	T2.mbb.glcm.correlation.2.mean.
4299	T2.mbb.glcm.correlation.2.range.
4300	T2.mbb.glcm.correlation.3.mean.
4301	T2.mbb.glcm.correlation.3.range.
4302	T2.mbb.glcm.correlation.4.mean.
4303	T2.mbb.glcm.correlation.4.range.
4304	T2.mbb.glcm.ASM.1.mean.

4305	T2.mbb.glcm.ASM.1.range.
4306	T2.mbb.glcm.ASM.2.mean.
4307	T2.mbb.glcm.ASM.2.range.
4308	T2.mbb.glcm.ASM.3.mean.
4309	T2.mbb.glcm.ASM.3.range.
4310	T2.mbb.glcm.ASM.4.mean.
4311	T2.mbb.glcm.ASM.4.range.
4312	T2.3.lbp.1.0.
4313	T2.3.lbp.1.1.
4314	T2.3.lbp.1.2.
4315	T2.3.lbp.1.3.
4316	T2.3.lbp.1.4.
4317	T2.3.lbp.1.5.
4318	T2.3.lbp.1.6.
4319	T2.3.lbp.1.7.
4320	T2.3.lbp.1.8.
4321	T2.3.lbp.1.9.
4322	T2.7.lbp.3.0.
4323	T2.7.lbp.3.1.
4324	T2.7.lbp.3.2.
4325	T2.7.lbp.3.3.
4326	T2.7.lbp.3.4.
4327	T2.7.lbp.3.5.
4328	T2.7.lbp.3.6.
4329	T2.7.lbp.3.7.
4330	T2.7.lbp.3.8.
4331	T2.7.lbp.3.9.
4332	T2.11.lbp.5.0.
4333	T2.11.lbp.5.1.
4334	T2.11.lbp.5.2.
4335	T2.11.lbp.5.3.
4336	T2.11.lbp.5.4.
4337	T2.11.lbp.5.5.
4338	T2.11.lbp.5.6.
4339	T2.11.lbp.5.7.
4340	T2.11.lbp.5.8.
4341	T2.11.lbp.5.9.

4342	T2.15.lbp.7.0.
4343	T2.15.lbp.7.1.
4344	T2.15.lbp.7.2.
4345	T2.15.lbp.7.3.
4346	T2.15.lbp.7.4.
4347	T2.15.lbp.7.5.
4348	T2.15.lbp.7.6.
4349	T2.15.lbp.7.7.
4350	T2.15.lbp.7.8.
4351	T2.15.lbp.7.9.
4352	T2.19.lbp.9.0.
4353	T2.19.lbp.9.1.
4354	T2.19.lbp.9.2.
4355	T2.19.lbp.9.3.
4356	T2.19.lbp.9.4.
4357	T2.19.lbp.9.5.
4358	T2.19.lbp.9.6.
4359	T2.19.lbp.9.7.
4360	T2.19.lbp.9.8.
4361	T2.19.lbp.9.9.
4362	T2.23.lbp.11.0.
4363	T2.23.lbp.11.1.
4364	T2.23.lbp.11.2.
4365	T2.23.lbp.11.3.
4366	T2.23.lbp.11.4.
4367	T2.23.lbp.11.5.
4368	T2.23.lbp.11.6.
4369	T2.23.lbp.11.7.
4370	T2.23.lbp.11.8.
4371	T2.23.lbp.11.9.
4372	T2.27.lbp.13.0.
4373	T2.27.lbp.13.1.
4374	T2.27.lbp.13.2.
4375	T2.27.lbp.13.3.
4376	T2.27.lbp.13.4.
4377	T2.27.lbp.13.5.
4378	T2.27.lbp.13.6.

4379	T2.27.lbp.13.7.
4380	T2.27.lbp.13.8.
4381	T2.27.lbp.13.9.
4382	T2.31.lbp.15.0.
4383	T2.31.lbp.15.1.
4384	T2.31.lbp.15.2.
4385	T2.31.lbp.15.3.
4386	T2.31.lbp.15.4.
4387	T2.31.lbp.15.5.
4388	T2.31.lbp.15.6.
4389	T2.31.lbp.15.7.
4390	T2.31.lbp.15.8.
4391	T2.31.lbp.15.9.
4392	T2.35.lbp.17.0.
4393	T2.35.lbp.17.1.
4394	T2.35.lbp.17.2.
4395	T2.35.lbp.17.3.
4396	T2.35.lbp.17.4.
4397	T2.35.lbp.17.5.
4398	T2.35.lbp.17.6.
4399	T2.35.lbp.17.7.
4400	T2.35.lbp.17.8.
4401	T2.35.lbp.17.9.
4402	T2.3.hog
4403	T2.7.hog
4404	T2.11.hog
4405	T2.15.hog
4406	T2.19.hog
4407	T2.23.hog
4408	T2.27.hog
4409	T2.31.hog
4410	T2.35.hog
4411	T2.3.gabor.1.0.1.mean.
4412	T2.3.gabor.1.0.1.var.
4413	T2.3.gabor.1.0.1.absmean.
4414	T2.3.gabor.1.0.1.mag.
4415	T2.3.gabor.1.0.2.mean.

4416	T2.3.gabor.1.0.2.var.
4417	T2.3.gabor.1.0.2.absmean.
4418	T2.3.gabor.1.0.2.mag.
4419	T2.3.gabor.1.0.3.mean.
4420	T2.3.gabor.1.0.3.var.
4421	T2.3.gabor.1.0.3.absmean.
4422	T2.3.gabor.1.0.3.mag.
4423	T2.3.gabor.1.0.4.mean.
4424	T2.3.gabor.1.0.4.var.
4425	T2.3.gabor.1.0.4.absmean.
4426	T2.3.gabor.1.0.4.mag.
4427	T2.3.gabor.1.0.5.mean.
4428	T2.3.gabor.1.0.5.var.
4429	T2.3.gabor.1.0.5.absmean.
4430	T2.3.gabor.1.0.5.mag.
4431	T2.3.gabor.2.0.1.mean.
4432	T2.3.gabor.2.0.1.var.
4433	T2.3.gabor.2.0.1.absmean.
4434	T2.3.gabor.2.0.1.mag.
4435	T2.3.gabor.2.0.2.mean.
4436	T2.3.gabor.2.0.2.var.
4437	T2.3.gabor.2.0.2.absmean.
4438	T2.3.gabor.2.0.2.mag.
4439	T2.3.gabor.2.0.3.mean.
4440	T2.3.gabor.2.0.3.var.
4441	T2.3.gabor.2.0.3.absmean.
4442	T2.3.gabor.2.0.3.mag.
4443	T2.3.gabor.2.0.4.mean.
4444	T2.3.gabor.2.0.4.var.
4445	T2.3.gabor.2.0.4.absmean.
4446	T2.3.gabor.2.0.4.mag.
4447	T2.3.gabor.2.0.5.mean.
4448	T2.3.gabor.2.0.5.var.
4449	T2.3.gabor.2.0.5.absmean.
4450	T2.3.gabor.2.0.5.mag.
4451	T2.3.gabor.3.0.1.mean.
4452	T2.3.gabor.3.0.1.var.

4453	T2.3.gabor.3.0.1.absmean.
4454	T2.3.gabor.3.0.1.mag.
4455	T2.3.gabor.3.0.2.mean.
4456	T2.3.gabor.3.0.2.var.
4457	T2.3.gabor.3.0.2.absmean.
4458	T2.3.gabor.3.0.2.mag.
4459	T2.3.gabor.3.0.3.mean.
4460	T2.3.gabor.3.0.3.var.
4461	T2.3.gabor.3.0.3.absmean.
4462	T2.3.gabor.3.0.3.mag.
4463	T2.3.gabor.3.0.4.mean.
4464	T2.3.gabor.3.0.4.var.
4465	T2.3.gabor.3.0.4.absmean.
4466	T2.3.gabor.3.0.4.mag.
4467	T2.3.gabor.3.0.5.mean.
4468	T2.3.gabor.3.0.5.var.
4469	T2.3.gabor.3.0.5.absmean.
4470	T2.3.gabor.3.0.5.mag.
4471	T2.7.gabor.1.0.1.mean.
4472	T2.7.gabor.1.0.1.var.
4473	T2.7.gabor.1.0.1.absmean.
4474	T2.7.gabor.1.0.1.mag.
4475	T2.7.gabor.1.0.2.mean.
4476	T2.7.gabor.1.0.2.var.
4477	T2.7.gabor.1.0.2.absmean.
4478	T2.7.gabor.1.0.2.mag.
4479	T2.7.gabor.1.0.3.mean.
4480	T2.7.gabor.1.0.3.var.
4481	T2.7.gabor.1.0.3.absmean.
4482	T2.7.gabor.1.0.3.mag.
4483	T2.7.gabor.1.0.4.mean.
4484	T2.7.gabor.1.0.4.var.
4485	T2.7.gabor.1.0.4.absmean.
4486	T2.7.gabor.1.0.4.mag.
4487	T2.7.gabor.1.0.5.mean.
4488	T2.7.gabor.1.0.5.var.
4489	T2.7.gabor.1.0.5.absmean.

4490	T2.7.gabor.1.0.5.mag.
4491	T2.7.gabor.2.0.1.mean.
4492	T2.7.gabor.2.0.1.var.
4493	T2.7.gabor.2.0.1.absmean.
4494	T2.7.gabor.2.0.1.mag.
4495	T2.7.gabor.2.0.2.mean.
4496	T2.7.gabor.2.0.2.var.
4497	T2.7.gabor.2.0.2.absmean.
4498	T2.7.gabor.2.0.2.mag.
4499	T2.7.gabor.2.0.3.mean.
4500	T2.7.gabor.2.0.3.var.
4501	T2.7.gabor.2.0.3.absmean.
4502	T2.7.gabor.2.0.3.mag.
4503	T2.7.gabor.2.0.4.mean.
4504	T2.7.gabor.2.0.4.var.
4505	T2.7.gabor.2.0.4.absmean.
4506	T2.7.gabor.2.0.4.mag.
4507	T2.7.gabor.2.0.5.mean.
4508	T2.7.gabor.2.0.5.var.
4509	T2.7.gabor.2.0.5.absmean.
4510	T2.7.gabor.2.0.5.mag.
4511	T2.7.gabor.3.0.1.mean.
4512	T2.7.gabor.3.0.1.var.
4513	T2.7.gabor.3.0.1.absmean.
4514	T2.7.gabor.3.0.1.mag.
4515	T2.7.gabor.3.0.2.mean.
4516	T2.7.gabor.3.0.2.var.
4517	T2.7.gabor.3.0.2.absmean.
4518	T2.7.gabor.3.0.2.mag.
4519	T2.7.gabor.3.0.3.mean.
4520	T2.7.gabor.3.0.3.var.
4521	T2.7.gabor.3.0.3.absmean.
4522	T2.7.gabor.3.0.3.mag.
4523	T2.7.gabor.3.0.4.mean.
4524	T2.7.gabor.3.0.4.var.
4525	T2.7.gabor.3.0.4.absmean.
4526	T2.7.gabor.3.0.4.mag.

4527	T2.7.gabor.3.0.5.mean.
4528	T2.7.gabor.3.0.5.var.
4529	T2.7.gabor.3.0.5.absmean.
4530	T2.7.gabor.3.0.5.mag.
4531	T2.11.gabor.1.0.1.mean.
4532	T2.11.gabor.1.0.1.var.
4533	T2.11.gabor.1.0.1.absmean.
4534	T2.11.gabor.1.0.1.mag.
4535	T2.11.gabor.1.0.2.mean.
4536	T2.11.gabor.1.0.2.var.
4537	T2.11.gabor.1.0.2.absmean.
4538	T2.11.gabor.1.0.2.mag.
4539	T2.11.gabor.1.0.3.mean.
4540	T2.11.gabor.1.0.3.var.
4541	T2.11.gabor.1.0.3.absmean.
4542	T2.11.gabor.1.0.3.mag.
4543	T2.11.gabor.1.0.4.mean.
4544	T2.11.gabor.1.0.4.var.
4545	T2.11.gabor.1.0.4.absmean.
4546	T2.11.gabor.1.0.4.mag.
4547	T2.11.gabor.1.0.5.mean.
4548	T2.11.gabor.1.0.5.var.
4549	T2.11.gabor.1.0.5.absmean.
4550	T2.11.gabor.1.0.5.mag.
4551	T2.11.gabor.2.0.1.mean.
4552	T2.11.gabor.2.0.1.var.
4553	T2.11.gabor.2.0.1.absmean.
4554	T2.11.gabor.2.0.1.mag.
4555	T2.11.gabor.2.0.2.mean.
4556	T2.11.gabor.2.0.2.var.
4557	T2.11.gabor.2.0.2.absmean.
4558	T2.11.gabor.2.0.2.mag.
4559	T2.11.gabor.2.0.3.mean.
4560	T2.11.gabor.2.0.3.var.
4561	T2.11.gabor.2.0.3.absmean.
4562	T2.11.gabor.2.0.3.mag.
4563	T2.11.gabor.2.0.4.mean.

4564	T2.11.gabor.2.0.4.var.
4565	T2.11.gabor.2.0.4.absmean.
4566	T2.11.gabor.2.0.4.mag.
4567	T2.11.gabor.2.0.5.mean.
4568	T2.11.gabor.2.0.5.var.
4569	T2.11.gabor.2.0.5.absmean.
4570	T2.11.gabor.2.0.5.mag.
4571	T2.11.gabor.3.0.1.mean.
4572	T2.11.gabor.3.0.1.var.
4573	T2.11.gabor.3.0.1.absmean.
4574	T2.11.gabor.3.0.1.mag.
4575	T2.11.gabor.3.0.2.mean.
4576	T2.11.gabor.3.0.2.var.
4577	T2.11.gabor.3.0.2.absmean.
4578	T2.11.gabor.3.0.2.mag.
4579	T2.11.gabor.3.0.3.mean.
4580	T2.11.gabor.3.0.3.var.
4581	T2.11.gabor.3.0.3.absmean.
4582	T2.11.gabor.3.0.3.mag.
4583	T2.11.gabor.3.0.4.mean.
4584	T2.11.gabor.3.0.4.var.
4585	T2.11.gabor.3.0.4.absmean.
4586	T2.11.gabor.3.0.4.mag.
4587	T2.11.gabor.3.0.5.mean.
4588	T2.11.gabor.3.0.5.var.
4589	T2.11.gabor.3.0.5.absmean.
4590	T2.11.gabor.3.0.5.mag.
4591	T2.15.gabor.1.0.1.mean.
4592	T2.15.gabor.1.0.1.var.
4593	T2.15.gabor.1.0.1.absmean.
4594	T2.15.gabor.1.0.1.mag.
4595	T2.15.gabor.1.0.2.mean.
4596	T2.15.gabor.1.0.2.var.
4597	T2.15.gabor.1.0.2.absmean.
4598	T2.15.gabor.1.0.2.mag.
4599	T2.15.gabor.1.0.3.mean.
4600	T2.15.gabor.1.0.3.var.

4601	T2.15.gabor.1.0.3.absmean.
4602	T2.15.gabor.1.0.3.mag.
4603	T2.15.gabor.1.0.4.mean.
4604	T2.15.gabor.1.0.4.var.
4605	T2.15.gabor.1.0.4.absmean.
4606	T2.15.gabor.1.0.4.mag.
4607	T2.15.gabor.1.0.5.mean.
4608	T2.15.gabor.1.0.5.var.
4609	T2.15.gabor.1.0.5.absmean.
4610	T2.15.gabor.1.0.5.mag.
4611	T2.15.gabor.2.0.1.mean.
4612	T2.15.gabor.2.0.1.var.
4613	T2.15.gabor.2.0.1.absmean.
4614	T2.15.gabor.2.0.1.mag.
4615	T2.15.gabor.2.0.2.mean.
4616	T2.15.gabor.2.0.2.var.
4617	T2.15.gabor.2.0.2.absmean.
4618	T2.15.gabor.2.0.2.mag.
4619	T2.15.gabor.2.0.3.mean.
4620	T2.15.gabor.2.0.3.var.
4621	T2.15.gabor.2.0.3.absmean.
4622	T2.15.gabor.2.0.3.mag.
4623	T2.15.gabor.2.0.4.mean.
4624	T2.15.gabor.2.0.4.var.
4625	T2.15.gabor.2.0.4.absmean.
4626	T2.15.gabor.2.0.4.mag.
4627	T2.15.gabor.2.0.5.mean.
4628	T2.15.gabor.2.0.5.var.
4629	T2.15.gabor.2.0.5.absmean.
4630	T2.15.gabor.2.0.5.mag.
4631	T2.15.gabor.3.0.1.mean.
4632	T2.15.gabor.3.0.1.var.
4633	T2.15.gabor.3.0.1.absmean.
4634	T2.15.gabor.3.0.1.mag.
4635	T2.15.gabor.3.0.2.mean.
4636	T2.15.gabor.3.0.2.var.
4637	T2.15.gabor.3.0.2.absmean.

4638	T2.15.gabor.3.0.2.mag.
4639	T2.15.gabor.3.0.3.mean.
4640	T2.15.gabor.3.0.3.var.
4641	T2.15.gabor.3.0.3.absmean.
4642	T2.15.gabor.3.0.3.mag.
4643	T2.15.gabor.3.0.4.mean.
4644	T2.15.gabor.3.0.4.var.
4645	T2.15.gabor.3.0.4.absmean.
4646	T2.15.gabor.3.0.4.mag.
4647	T2.15.gabor.3.0.5.mean.
4648	T2.15.gabor.3.0.5.var.
4649	T2.15.gabor.3.0.5.absmean.
4650	T2.15.gabor.3.0.5.mag.
4651	T2.19.gabor.1.0.1.mean.
4652	T2.19.gabor.1.0.1.var.
4653	T2.19.gabor.1.0.1.absmean.
4654	T2.19.gabor.1.0.1.mag.
4655	T2.19.gabor.1.0.2.mean.
4656	T2.19.gabor.1.0.2.var.
4657	T2.19.gabor.1.0.2.absmean.
4658	T2.19.gabor.1.0.2.mag.
4659	T2.19.gabor.1.0.3.mean.
4660	T2.19.gabor.1.0.3.var.
4661	T2.19.gabor.1.0.3.absmean.
4662	T2.19.gabor.1.0.3.mag.
4663	T2.19.gabor.1.0.4.mean.
4664	T2.19.gabor.1.0.4.var.
4665	T2.19.gabor.1.0.4.absmean.
4666	T2.19.gabor.1.0.4.mag.
4667	T2.19.gabor.1.0.5.mean.
4668	T2.19.gabor.1.0.5.var.
4669	T2.19.gabor.1.0.5.absmean.
4670	T2.19.gabor.1.0.5.mag.
4671	T2.19.gabor.2.0.1.mean.
4672	T2.19.gabor.2.0.1.var.
4673	T2.19.gabor.2.0.1.absmean.
4674	T2.19.gabor.2.0.1.mag.

4675	T2.19.gabor.2.0.2.mean.
4676	T2.19.gabor.2.0.2.var.
4677	T2.19.gabor.2.0.2.absmean.
4678	T2.19.gabor.2.0.2.mag.
4679	T2.19.gabor.2.0.3.mean.
4680	T2.19.gabor.2.0.3.var.
4681	T2.19.gabor.2.0.3.absmean.
4682	T2.19.gabor.2.0.3.mag.
4683	T2.19.gabor.2.0.4.mean.
4684	T2.19.gabor.2.0.4.var.
4685	T2.19.gabor.2.0.4.absmean.
4686	T2.19.gabor.2.0.4.mag.
4687	T2.19.gabor.2.0.5.mean.
4688	T2.19.gabor.2.0.5.var.
4689	T2.19.gabor.2.0.5.absmean.
4690	T2.19.gabor.2.0.5.mag.
4691	T2.19.gabor.3.0.1.mean.
4692	T2.19.gabor.3.0.1.var.
4693	T2.19.gabor.3.0.1.absmean.
4694	T2.19.gabor.3.0.1.mag.
4695	T2.19.gabor.3.0.2.mean.
4696	T2.19.gabor.3.0.2.var.
4697	T2.19.gabor.3.0.2.absmean.
4698	T2.19.gabor.3.0.2.mag.
4699	T2.19.gabor.3.0.3.mean.
4700	T2.19.gabor.3.0.3.var.
4701	T2.19.gabor.3.0.3.absmean.
4702	T2.19.gabor.3.0.3.mag.
4703	T2.19.gabor.3.0.4.mean.
4704	T2.19.gabor.3.0.4.var.
4705	T2.19.gabor.3.0.4.absmean.
4706	T2.19.gabor.3.0.4.mag.
4707	T2.19.gabor.3.0.5.mean.
4708	T2.19.gabor.3.0.5.var.
4709	T2.19.gabor.3.0.5.absmean.
4710	T2.19.gabor.3.0.5.mag.
4711	T2.23.gabor.1.0.1.mean.

4712	T2.23.gabor.1.0.1.var.
4713	T2.23.gabor.1.0.1.absmean.
4714	T2.23.gabor.1.0.1.mag.
4715	T2.23.gabor.1.0.2.mean.
4716	T2.23.gabor.1.0.2.var.
4717	T2.23.gabor.1.0.2.absmean.
4718	T2.23.gabor.1.0.2.mag.
4719	T2.23.gabor.1.0.3.mean.
4720	T2.23.gabor.1.0.3.var.
4721	T2.23.gabor.1.0.3.absmean.
4722	T2.23.gabor.1.0.3.mag.
4723	T2.23.gabor.1.0.4.mean.
4724	T2.23.gabor.1.0.4.var.
4725	T2.23.gabor.1.0.4.absmean.
4726	T2.23.gabor.1.0.4.mag.
4727	T2.23.gabor.1.0.5.mean.
4728	T2.23.gabor.1.0.5.var.
4729	T2.23.gabor.1.0.5.absmean.
4730	T2.23.gabor.1.0.5.mag.
4731	T2.23.gabor.2.0.1.mean.
4732	T2.23.gabor.2.0.1.var.
4733	T2.23.gabor.2.0.1.absmean.
4734	T2.23.gabor.2.0.1.mag.
4735	T2.23.gabor.2.0.2.mean.
4736	T2.23.gabor.2.0.2.var.
4737	T2.23.gabor.2.0.2.absmean.
4738	T2.23.gabor.2.0.2.mag.
4739	T2.23.gabor.2.0.3.mean.
4740	T2.23.gabor.2.0.3.var.
4741	T2.23.gabor.2.0.3.absmean.
4742	T2.23.gabor.2.0.3.mag.
4743	T2.23.gabor.2.0.4.mean.
4744	T2.23.gabor.2.0.4.var.
4745	T2.23.gabor.2.0.4.absmean.
4746	T2.23.gabor.2.0.4.mag.
4747	T2.23.gabor.2.0.5.mean.
4748	T2.23.gabor.2.0.5.var.

4749	T2.23.gabor.2.0.5.absmean.
4750	T2.23.gabor.2.0.5.mag.
4751	T2.23.gabor.3.0.1.mean.
4752	T2.23.gabor.3.0.1.var.
4753	T2.23.gabor.3.0.1.absmean.
4754	T2.23.gabor.3.0.1.mag.
4755	T2.23.gabor.3.0.2.mean.
4756	T2.23.gabor.3.0.2.var.
4757	T2.23.gabor.3.0.2.absmean.
4758	T2.23.gabor.3.0.2.mag.
4759	T2.23.gabor.3.0.3.mean.
4760	T2.23.gabor.3.0.3.var.
4761	T2.23.gabor.3.0.3.absmean.
4762	T2.23.gabor.3.0.3.mag.
4763	T2.23.gabor.3.0.4.mean.
4764	T2.23.gabor.3.0.4.var.
4765	T2.23.gabor.3.0.4.absmean.
4766	T2.23.gabor.3.0.4.mag.
4767	T2.23.gabor.3.0.5.mean.
4768	T2.23.gabor.3.0.5.var.
4769	T2.23.gabor.3.0.5.absmean.
4770	T2.23.gabor.3.0.5.mag.
4771	T2.27.gabor.1.0.1.mean.
4772	T2.27.gabor.1.0.1.var.
4773	T2.27.gabor.1.0.1.absmean.
4774	T2.27.gabor.1.0.1.mag.
4775	T2.27.gabor.1.0.2.mean.
4776	T2.27.gabor.1.0.2.var.
4777	T2.27.gabor.1.0.2.absmean.
4778	T2.27.gabor.1.0.2.mag.
4779	T2.27.gabor.1.0.3.mean.
4780	T2.27.gabor.1.0.3.var.
4781	T2.27.gabor.1.0.3.absmean.
4782	T2.27.gabor.1.0.3.mag.
4783	T2.27.gabor.1.0.4.mean.
4784	T2.27.gabor.1.0.4.var.
4785	T2.27.gabor.1.0.4.absmean.

4786	T2.27.gabor.1.0.4.mag.
4787	T2.27.gabor.1.0.5.mean.
4788	T2.27.gabor.1.0.5.var.
4789	T2.27.gabor.1.0.5.absmean.
4790	T2.27.gabor.1.0.5.mag.
4791	T2.27.gabor.2.0.1.mean.
4792	T2.27.gabor.2.0.1.var.
4793	T2.27.gabor.2.0.1.absmean.
4794	T2.27.gabor.2.0.1.mag.
4795	T2.27.gabor.2.0.2.mean.
4796	T2.27.gabor.2.0.2.var.
4797	T2.27.gabor.2.0.2.absmean.
4798	T2.27.gabor.2.0.2.mag.
4799	T2.27.gabor.2.0.3.mean.
4800	T2.27.gabor.2.0.3.var.
4801	T2.27.gabor.2.0.3.absmean.
4802	T2.27.gabor.2.0.3.mag.
4803	T2.27.gabor.2.0.4.mean.
4804	T2.27.gabor.2.0.4.var.
4805	T2.27.gabor.2.0.4.absmean.
4806	T2.27.gabor.2.0.4.mag.
4807	T2.27.gabor.2.0.5.mean.
4808	T2.27.gabor.2.0.5.var.
4809	T2.27.gabor.2.0.5.absmean.
4810	T2.27.gabor.2.0.5.mag.
4811	T2.27.gabor.3.0.1.mean.
4812	T2.27.gabor.3.0.1.var.
4813	T2.27.gabor.3.0.1.absmean.
4814	T2.27.gabor.3.0.1.mag.
4815	T2.27.gabor.3.0.2.mean.
4816	T2.27.gabor.3.0.2.var.
4817	T2.27.gabor.3.0.2.absmean.
4818	T2.27.gabor.3.0.2.mag.
4819	T2.27.gabor.3.0.3.mean.
4820	T2.27.gabor.3.0.3.var.
4821	T2.27.gabor.3.0.3.absmean.
4822	T2.27.gabor.3.0.3.mag.

4823	T2.27.gabor.3.0.4.mean.
4824	T2.27.gabor.3.0.4.var.
4825	T2.27.gabor.3.0.4.absmean.
4826	T2.27.gabor.3.0.4.mag.
4827	T2.27.gabor.3.0.5.mean.
4828	T2.27.gabor.3.0.5.var.
4829	T2.27.gabor.3.0.5.absmean.
4830	T2.27.gabor.3.0.5.mag.
4831	T2.31.gabor.1.0.1.mean.
4832	T2.31.gabor.1.0.1.var.
4833	T2.31.gabor.1.0.1.absmean.
4834	T2.31.gabor.1.0.1.mag.
4835	T2.31.gabor.1.0.2.mean.
4836	T2.31.gabor.1.0.2.var.
4837	T2.31.gabor.1.0.2.absmean.
4838	T2.31.gabor.1.0.2.mag.
4839	T2.31.gabor.1.0.3.mean.
4840	T2.31.gabor.1.0.3.var.
4841	T2.31.gabor.1.0.3.absmean.
4842	T2.31.gabor.1.0.3.mag.
4843	T2.31.gabor.1.0.4.mean.
4844	T2.31.gabor.1.0.4.var.
4845	T2.31.gabor.1.0.4.absmean.
4846	T2.31.gabor.1.0.4.mag.
4847	T2.31.gabor.1.0.5.mean.
4848	T2.31.gabor.1.0.5.var.
4849	T2.31.gabor.1.0.5.absmean.
4850	T2.31.gabor.1.0.5.mag.
4851	T2.31.gabor.2.0.1.mean.
4852	T2.31.gabor.2.0.1.var.
4853	T2.31.gabor.2.0.1.absmean.
4854	T2.31.gabor.2.0.1.mag.
4855	T2.31.gabor.2.0.2.mean.
4856	T2.31.gabor.2.0.2.var.
4857	T2.31.gabor.2.0.2.absmean.
4858	T2.31.gabor.2.0.2.mag.
4859	T2.31.gabor.2.0.3.mean.

4860	T2.31.gabor.2.0.3.var.
4861	T2.31.gabor.2.0.3.absmean.
4862	T2.31.gabor.2.0.3.mag.
4863	T2.31.gabor.2.0.4.mean.
4864	T2.31.gabor.2.0.4.var.
4865	T2.31.gabor.2.0.4.absmean.
4866	T2.31.gabor.2.0.4.mag.
4867	T2.31.gabor.2.0.5.mean.
4868	T2.31.gabor.2.0.5.var.
4869	T2.31.gabor.2.0.5.absmean.
4870	T2.31.gabor.2.0.5.mag.
4871	T2.31.gabor.3.0.1.mean.
4872	T2.31.gabor.3.0.1.var.
4873	T2.31.gabor.3.0.1.absmean.
4874	T2.31.gabor.3.0.1.mag.
4875	T2.31.gabor.3.0.2.mean.
4876	T2.31.gabor.3.0.2.var.
4877	T2.31.gabor.3.0.2.absmean.
4878	T2.31.gabor.3.0.2.mag.
4879	T2.31.gabor.3.0.3.mean.
4880	T2.31.gabor.3.0.3.var.
4881	T2.31.gabor.3.0.3.absmean.
4882	T2.31.gabor.3.0.3.mag.
4883	T2.31.gabor.3.0.4.mean.
4884	T2.31.gabor.3.0.4.var.
4885	T2.31.gabor.3.0.4.absmean.
4886	T2.31.gabor.3.0.4.mag.
4887	T2.31.gabor.3.0.5.mean.
4888	T2.31.gabor.3.0.5.var.
4889	T2.31.gabor.3.0.5.absmean.
4890	T2.31.gabor.3.0.5.mag.
4891	T2.35.gabor.1.0.1.mean.
4892	T2.35.gabor.1.0.1.var.
4893	T2.35.gabor.1.0.1.absmean.
4894	T2.35.gabor.1.0.1.mag.
4895	T2.35.gabor.1.0.2.mean.
4896	T2.35.gabor.1.0.2.var.

4897	T2.35.gabor.1.0.2.absmean.
4898	T2.35.gabor.1.0.2.mag.
4899	T2.35.gabor.1.0.3.mean.
4900	T2.35.gabor.1.0.3.var.
4901	T2.35.gabor.1.0.3.absmean.
4902	T2.35.gabor.1.0.3.mag.
4903	T2.35.gabor.1.0.4.mean.
4904	T2.35.gabor.1.0.4.var.
4905	T2.35.gabor.1.0.4.absmean.
4906	T2.35.gabor.1.0.4.mag.
4907	T2.35.gabor.1.0.5.mean.
4908	T2.35.gabor.1.0.5.var.
4909	T2.35.gabor.1.0.5.absmean.
4910	T2.35.gabor.1.0.5.mag.
4911	T2.35.gabor.2.0.1.mean.
4912	T2.35.gabor.2.0.1.var.
4913	T2.35.gabor.2.0.1.absmean.
4914	T2.35.gabor.2.0.1.mag.
4915	T2.35.gabor.2.0.2.mean.
4916	T2.35.gabor.2.0.2.var.
4917	T2.35.gabor.2.0.2.absmean.
4918	T2.35.gabor.2.0.2.mag.
4919	T2.35.gabor.2.0.3.mean.
4920	T2.35.gabor.2.0.3.var.
4921	T2.35.gabor.2.0.3.absmean.
4922	T2.35.gabor.2.0.3.mag.
4923	T2.35.gabor.2.0.4.mean.
4924	T2.35.gabor.2.0.4.var.
4925	T2.35.gabor.2.0.4.absmean.
4926	T2.35.gabor.2.0.4.mag.
4927	T2.35.gabor.2.0.5.mean.
4928	T2.35.gabor.2.0.5.var.
4929	T2.35.gabor.2.0.5.absmean.
4930	T2.35.gabor.2.0.5.mag.
4931	T2.35.gabor.3.0.1.mean.
4932	T2.35.gabor.3.0.1.var.
4933	T2.35.gabor.3.0.1.absmean.

4934	T2.35.gabor.3.0.1.mag.
4935	T2.35.gabor.3.0.2.mean.
4936	T2.35.gabor.3.0.2.var.
4937	T2.35.gabor.3.0.2.absmean.
4938	T2.35.gabor.3.0.2.mag.
4939	T2.35.gabor.3.0.3.mean.
4940	T2.35.gabor.3.0.3.var.
4941	T2.35.gabor.3.0.3.absmean.
4942	T2.35.gabor.3.0.3.mag.
4943	T2.35.gabor.3.0.4.mean.
4944	T2.35.gabor.3.0.4.var.
4945	T2.35.gabor.3.0.4.absmean.
4946	T2.35.gabor.3.0.4.mag.
4947	T2.35.gabor.3.0.5.mean.
4948	T2.35.gabor.3.0.5.var.
4949	T2.35.gabor.3.0.5.absmean.
4950	T2.35.gabor.3.0.5.mag.
4951	T2.3.haar.1.1.aav.
4952	T2.3.haar.1.1.std.
4953	T2.3.haar.1.2.aav.
4954	T2.3.haar.1.2.std.
4955	T2.3.haar.1.3.aav.
4956	T2.3.haar.1.3.std.
4957	T2.3.haar.2.1.aav.
4958	T2.3.haar.2.1.std.
4959	T2.3.haar.2.2.aav.
4960	T2.3.haar.2.2.std.
4961	T2.3.haar.2.3.aav.
4962	T2.3.haar.2.3.std.
4963	T2.3.haar.3.1.aav.
4964	T2.3.haar.3.1.std.
4965	T2.3.haar.3.2.aav.
4966	T2.3.haar.3.2.std.
4967	T2.3.haar.3.3.aav.
4968	T2.3.haar.3.3.std.
4969	T2.3.haar.4.1.aav.
4970	T2.3.haar.4.1.std.

4971	T2.3.haar.4.2.aav.
4972	T2.3.haar.4.2.std.
4973	T2.3.haar.4.3.aav.
4974	T2.3.haar.4.3.std.
4975	T2.7.haar.1.1.aav.
4976	T2.7.haar.1.1.std.
4977	T2.7.haar.1.2.aav.
4978	T2.7.haar.1.2.std.
4979	T2.7.haar.1.3.aav.
4980	T2.7.haar.1.3.std.
4981	T2.7.haar.2.1.aav.
4982	T2.7.haar.2.1.std.
4983	T2.7.haar.2.2.aav.
4984	T2.7.haar.2.2.std.
4985	T2.7.haar.2.3.aav.
4986	T2.7.haar.2.3.std.
4987	T2.7.haar.3.1.aav.
4988	T2.7.haar.3.1.std.
4989	T2.7.haar.3.2.aav.
4990	T2.7.haar.3.2.std.
4991	T2.7.haar.3.3.aav.
4992	T2.7.haar.3.3.std.
4993	T2.7.haar.4.1.aav.
4994	T2.7.haar.4.1.std.
4995	T2.7.haar.4.2.aav.
4996	T2.7.haar.4.2.std.
4997	T2.7.haar.4.3.aav.
4998	T2.7.haar.4.3.std.
4999	T2.11.haar.1.1.aav.
5000	T2.11.haar.1.1.std.
5001	T2.11.haar.1.2.aav.
5002	T2.11.haar.1.2.std.
5003	T2.11.haar.1.3.aav.
5004	T2.11.haar.1.3.std.
5005	T2.11.haar.2.1.aav.
5006	T2.11.haar.2.1.std.
5007	T2.11.haar.2.2.aav.

5008	T2.11.haar.2.2.std.
5009	T2.11.haar.2.3.aav.
5010	T2.11.haar.2.3.std.
5011	T2.11.haar.3.1.aav.
5012	T2.11.haar.3.1.std.
5013	T2.11.haar.3.2.aav.
5014	T2.11.haar.3.2.std.
5015	T2.11.haar.3.3.aav.
5016	T2.11.haar.3.3.std.
5017	T2.11.haar.4.1.aav.
5018	T2.11.haar.4.1.std.
5019	T2.11.haar.4.2.aav.
5020	T2.11.haar.4.2.std.
5021	T2.11.haar.4.3.aav.
5022	T2.11.haar.4.3.std.
5023	T2.15.haar.1.1.aav.
5024	T2.15.haar.1.1.std.
5025	T2.15.haar.1.2.aav.
5026	T2.15.haar.1.2.std.
5027	T2.15.haar.1.3.aav.
5028	T2.15.haar.1.3.std.
5029	T2.15.haar.2.1.aav.
5030	T2.15.haar.2.1.std.
5031	T2.15.haar.2.2.aav.
5032	T2.15.haar.2.2.std.
5033	T2.15.haar.2.3.aav.
5034	T2.15.haar.2.3.std.
5035	T2.15.haar.3.1.aav.
5036	T2.15.haar.3.1.std.
5037	T2.15.haar.3.2.aav.
5038	T2.15.haar.3.2.std.
5039	T2.15.haar.3.3.aav.
5040	T2.15.haar.3.3.std.
5041	T2.15.haar.4.1.aav.
5042	T2.15.haar.4.1.std.
5043	T2.15.haar.4.2.aav.
5044	T2.15.haar.4.2.std.

5045	T2.15.haar.4.3.aav.
5046	T2.15.haar.4.3.std.
5047	T2.19.haar.1.1.aav.
5048	T2.19.haar.1.1.std.
5049	T2.19.haar.1.2.aav.
5050	T2.19.haar.1.2.std.
5051	T2.19.haar.1.3.aav.
5052	T2.19.haar.1.3.std.
5053	T2.19.haar.2.1.aav.
5054	T2.19.haar.2.1.std.
5055	T2.19.haar.2.2.aav.
5056	T2.19.haar.2.2.std.
5057	T2.19.haar.2.3.aav.
5058	T2.19.haar.2.3.std.
5059	T2.19.haar.3.1.aav.
5060	T2.19.haar.3.1.std.
5061	T2.19.haar.3.2.aav.
5062	T2.19.haar.3.2.std.
5063	T2.19.haar.3.3.aav.
5064	T2.19.haar.3.3.std.
5065	T2.19.haar.4.1.aav.
5066	T2.19.haar.4.1.std.
5067	T2.19.haar.4.2.aav.
5068	T2.19.haar.4.2.std.
5069	T2.19.haar.4.3.aav.
5070	T2.19.haar.4.3.std.
5071	T2.23.haar.1.1.aav.
5072	T2.23.haar.1.1.std.
5073	T2.23.haar.1.2.aav.
5074	T2.23.haar.1.2.std.
5075	T2.23.haar.1.3.aav.
5076	T2.23.haar.1.3.std.
5077	T2.23.haar.2.1.aav.
5078	T2.23.haar.2.1.std.
5079	T2.23.haar.2.2.aav.
5080	T2.23.haar.2.2.std.
5081	T2.23.haar.2.3.aav.

5082	T2.23.haar.2.3.std.
5083	T2.23.haar.3.1.aav.
5084	T2.23.haar.3.1.std.
5085	T2.23.haar.3.2.aav.
5086	T2.23.haar.3.2.std.
5087	T2.23.haar.3.3.aav.
5088	T2.23.haar.3.3.std.
5089	T2.23.haar.4.1.aav.
5090	T2.23.haar.4.1.std.
5091	T2.23.haar.4.2.aav.
5092	T2.23.haar.4.2.std.
5093	T2.23.haar.4.3.aav.
5094	T2.23.haar.4.3.std.
5095	T2.27.haar.1.1.aav.
5096	T2.27.haar.1.1.std.
5097	T2.27.haar.1.2.aav.
5098	T2.27.haar.1.2.std.
5099	T2.27.haar.1.3.aav.
5100	T2.27.haar.1.3.std.
5101	T2.27.haar.2.1.aav.
5102	T2.27.haar.2.1.std.
5103	T2.27.haar.2.2.aav.
5104	T2.27.haar.2.2.std.
5105	T2.27.haar.2.3.aav.
5106	T2.27.haar.2.3.std.
5107	T2.27.haar.3.1.aav.
5108	T2.27.haar.3.1.std.
5109	T2.27.haar.3.2.aav.
5110	T2.27.haar.3.2.std.
5111	T2.27.haar.3.3.aav.
5112	T2.27.haar.3.3.std.
5113	T2.27.haar.4.1.aav.
5114	T2.27.haar.4.1.std.
5115	T2.27.haar.4.2.aav.
5116	T2.27.haar.4.2.std.
5117	T2.27.haar.4.3.aav.
5118	T2.27.haar.4.3.std.

5119	T2.31.haar.1.1.aav.
5120	T2.31.haar.1.1.std.
5121	T2.31.haar.1.2.aav.
5122	T2.31.haar.1.2.std.
5123	T2.31.haar.1.3.aav.
5124	T2.31.haar.1.3.std.
5125	T2.31.haar.2.1.aav.
5126	T2.31.haar.2.1.std.
5127	T2.31.haar.2.2.aav.
5128	T2.31.haar.2.2.std.
5129	T2.31.haar.2.3.aav.
5130	T2.31.haar.2.3.std.
5131	T2.31.haar.3.1.aav.
5132	T2.31.haar.3.1.std.
5133	T2.31.haar.3.2.aav.
5134	T2.31.haar.3.2.std.
5135	T2.31.haar.3.3.aav.
5136	T2.31.haar.3.3.std.
5137	T2.31.haar.4.1.aav.
5138	T2.31.haar.4.1.std.
5139	T2.31.haar.4.2.aav.
5140	T2.31.haar.4.2.std.
5141	T2.31.haar.4.3.aav.
5142	T2.31.haar.4.3.std.
5143	T2.35.haar.1.1.aav.
5144	T2.35.haar.1.1.std.
5145	T2.35.haar.1.2.aav.
5146	T2.35.haar.1.2.std.
5147	T2.35.haar.1.3.aav.
5148	T2.35.haar.1.3.std.
5149	T2.35.haar.2.1.aav.
5150	T2.35.haar.2.1.std.
5151	T2.35.haar.2.2.aav.
5152	T2.35.haar.2.2.std.
5153	T2.35.haar.2.3.aav.
5154	T2.35.haar.2.3.std.
5155	T2.35.haar.3.1.aav.

5156	T2.35.haar.3.1.std.
5157	T2.35.haar.3.2.aav.
5158	T2.35.haar.3.2.std.
5159	T2.35.haar.3.3.aav.
5160	T2.35.haar.3.3.std.
5161	T2.35.haar.4.1.aav.
5162	T2.35.haar.4.1.std.
5163	T2.35.haar.4.2.aav.
5164	T2.35.haar.4.2.std.
5165	T2.35.haar.4.3.aav.
5166	T2.35.haar.4.3.std.
5167	T2.3.hu.0.
5168	T2.3.hu.1.
5169	T2.3.hu.2.
5170	T2.3.hu.3.
5171	T2.3.hu.4.
5172	T2.3.hu.5.
5173	T2.3.hu.6.
5174	T2.7.hu.0.
5175	T2.7.hu.1.
5176	T2.7.hu.2.
5177	T2.7.hu.3.
5178	T2.7.hu.4.
5179	T2.7.hu.5.
5180	T2.7.hu.6.
5181	T2.11.hu.0.
5182	T2.11.hu.1.
5183	T2.11.hu.2.
5184	T2.11.hu.3.
5185	T2.11.hu.4.
5186	T2.11.hu.5.
5187	T2.11.hu.6.
5188	T2.15.hu.0.
5189	T2.15.hu.1.
5190	T2.15.hu.2.
5191	T2.15.hu.3.
5192	T2.15.hu.4.

5193	T2.15.hu.5.
5194	T2.15.hu.6.
5195	T2.19.hu.0.
5196	T2.19.hu.1.
5197	T2.19.hu.2.
5198	T2.19.hu.3.
5199	T2.19.hu.4.
5200	T2.19.hu.5.
5201	T2.19.hu.6.
5202	T2.23.hu.0.
5203	T2.23.hu.1.
5204	T2.23.hu.2.
5205	T2.23.hu.3.
5206	T2.23.hu.4.
5207	T2.23.hu.5.
5208	T2.23.hu.6.
5209	T2.27.hu.0.
5210	T2.27.hu.1.
5211	T2.27.hu.2.
5212	T2.27.hu.3.
5213	T2.27.hu.4.
5214	T2.27.hu.5.
5215	T2.27.hu.6.
5216	T2.31.hu.0.
5217	T2.31.hu.1.
5218	T2.31.hu.2.
5219	T2.31.hu.3.
5220	T2.31.hu.4.
5221	T2.31.hu.5.
5222	T2.31.hu.6.
5223	T2.35.hu.0.
5224	T2.35.hu.1.
5225	T2.35.hu.2.
5226	T2.35.hu.3.
5227	T2.35.hu.4.
5228	T2.35.hu.5.
5229	T2.35.hu.6.

5230	T2.3.zernike.0.
5231	T2.3.zernike.1.
5232	T2.3.zernike.2.
5233	T2.3.zernike.3.
5234	T2.3.zernike.4.
5235	T2.3.zernike.5.
5236	T2.3.zernike.6.
5237	T2.3.zernike.7.
5238	T2.3.zernike.8.
5239	T2.3.zernike.9.
5240	T2.3.zernike.10.
5241	T2.3.zernike.11.
5242	T2.3.zernike.12.
5243	T2.3.zernike.13.
5244	T2.3.zernike.14.
5245	T2.3.zernike.15.
5246	T2.3.zernike.16.
5247	T2.3.zernike.17.
5248	T2.3.zernike.18.
5249	T2.3.zernike.19.
5250	T2.3.zernike.20.
5251	T2.3.zernike.21.
5252	T2.3.zernike.22.
5253	T2.3.zernike.23.
5254	T2.3.zernike.24.
5255	T2.7.zernike.0.
5256	T2.7.zernike.1.
5257	T2.7.zernike.2.
5258	T2.7.zernike.3.
5259	T2.7.zernike.4.
5260	T2.7.zernike.5.
5261	T2.7.zernike.6.
5262	T2.7.zernike.7.
5263	T2.7.zernike.8.
5264	T2.7.zernike.9.
5265	T2.7.zernike.10.
5266	T2.7.zernike.11.

5267	T2.7.zernike.12.
5268	T2.7.zernike.13.
5269	T2.7.zernike.14.
5270	T2.7.zernike.15.
5271	T2.7.zernike.16.
5272	T2.7.zernike.17.
5273	T2.7.zernike.18.
5274	T2.7.zernike.19.
5275	T2.7.zernike.20.
5276	T2.7.zernike.21.
5277	T2.7.zernike.22.
5278	T2.7.zernike.23.
5279	T2.7.zernike.24.
5280	T2.11.zernike.0.
5281	T2.11.zernike.1.
5282	T2.11.zernike.2.
5283	T2.11.zernike.3.
5284	T2.11.zernike.4.
5285	T2.11.zernike.5.
5286	T2.11.zernike.6.
5287	T2.11.zernike.7.
5288	T2.11.zernike.8.
5289	T2.11.zernike.9.
5290	T2.11.zernike.10.
5291	T2.11.zernike.11.
5292	T2.11.zernike.12.
5293	T2.11.zernike.13.
5294	T2.11.zernike.14.
5295	T2.11.zernike.15.
5296	T2.11.zernike.16.
5297	T2.11.zernike.17.
5298	T2.11.zernike.18.
5299	T2.11.zernike.19.
5300	T2.11.zernike.20.
5301	T2.11.zernike.21.
5302	T2.11.zernike.22.
5303	T2.11.zernike.23.

5304	T2.11.zernike.24.
5305	T2.15.zernike.0.
5306	T2.15.zernike.1.
5307	T2.15.zernike.2.
5308	T2.15.zernike.3.
5309	T2.15.zernike.4.
5310	T2.15.zernike.5.
5311	T2.15.zernike.6.
5312	T2.15.zernike.7.
5313	T2.15.zernike.8.
5314	T2.15.zernike.9.
5315	T2.15.zernike.10.
5316	T2.15.zernike.11.
5317	T2.15.zernike.12.
5318	T2.15.zernike.13.
5319	T2.15.zernike.14.
5320	T2.15.zernike.15.
5321	T2.15.zernike.16.
5322	T2.15.zernike.17.
5323	T2.15.zernike.18.
5324	T2.15.zernike.19.
5325	T2.15.zernike.20.
5326	T2.15.zernike.21.
5327	T2.15.zernike.22.
5328	T2.15.zernike.23.
5329	T2.15.zernike.24.
5330	T2.19.zernike.0.
5331	T2.19.zernike.1.
5332	T2.19.zernike.2.
5333	T2.19.zernike.3.
5334	T2.19.zernike.4.
5335	T2.19.zernike.5.
5336	T2.19.zernike.6.
5337	T2.19.zernike.7.
5338	T2.19.zernike.8.
5339	T2.19.zernike.9.
5340	T2.19.zernike.10.

5341	T2.19.zernike.11.
5342	T2.19.zernike.12.
5343	T2.19.zernike.13.
5344	T2.19.zernike.14.
5345	T2.19.zernike.15.
5346	T2.19.zernike.16.
5347	T2.19.zernike.17.
5348	T2.19.zernike.18.
5349	T2.19.zernike.19.
5350	T2.19.zernike.20.
5351	T2.19.zernike.21.
5352	T2.19.zernike.22.
5353	T2.19.zernike.23.
5354	T2.19.zernike.24.
5355	T2.23.zernike.0.
5356	T2.23.zernike.1.
5357	T2.23.zernike.2.
5358	T2.23.zernike.3.
5359	T2.23.zernike.4.
5360	T2.23.zernike.5.
5361	T2.23.zernike.6.
5362	T2.23.zernike.7.
5363	T2.23.zernike.8.
5364	T2.23.zernike.9.
5365	T2.23.zernike.10.
5366	T2.23.zernike.11.
5367	T2.23.zernike.12.
5368	T2.23.zernike.13.
5369	T2.23.zernike.14.
5370	T2.23.zernike.15.
5371	T2.23.zernike.16.
5372	T2.23.zernike.17.
5373	T2.23.zernike.18.
5374	T2.23.zernike.19.
5375	T2.23.zernike.20.
5376	T2.23.zernike.21.
5377	T2.23.zernike.22.

5378	T2.23.zernike.23.
5379	T2.23.zernike.24.
5380	T2.27.zernike.0.
5381	T2.27.zernike.1.
5382	T2.27.zernike.2.
5383	T2.27.zernike.3.
5384	T2.27.zernike.4.
5385	T2.27.zernike.5.
5386	T2.27.zernike.6.
5387	T2.27.zernike.7.
5388	T2.27.zernike.8.
5389	T2.27.zernike.9.
5390	T2.27.zernike.10.
5391	T2.27.zernike.11.
5392	T2.27.zernike.12.
5393	T2.27.zernike.13.
5394	T2.27.zernike.14.
5395	T2.27.zernike.15.
5396	T2.27.zernike.16.
5397	T2.27.zernike.17.
5398	T2.27.zernike.18.
5399	T2.27.zernike.19.
5400	T2.27.zernike.20.
5401	T2.27.zernike.21.
5402	T2.27.zernike.22.
5403	T2.27.zernike.23.
5404	T2.27.zernike.24.
5405	T2.31.zernike.0.
5406	T2.31.zernike.1.
5407	T2.31.zernike.2.
5408	T2.31.zernike.3.
5409	T2.31.zernike.4.
5410	T2.31.zernike.5.
5411	T2.31.zernike.6.
5412	T2.31.zernike.7.
5413	T2.31.zernike.8.
5414	T2.31.zernike.9.

5415	T2.31.zernike.10.
5416	T2.31.zernike.11.
5417	T2.31.zernike.12.
5418	T2.31.zernike.13.
5419	T2.31.zernike.14.
5420	T2.31.zernike.15.
5421	T2.31.zernike.16.
5422	T2.31.zernike.17.
5423	T2.31.zernike.18.
5424	T2.31.zernike.19.
5425	T2.31.zernike.20.
5426	T2.31.zernike.21.
5427	T2.31.zernike.22.
5428	T2.31.zernike.23.
5429	T2.31.zernike.24.
5430	T2.35.zernike.0.
5431	T2.35.zernike.1.
5432	T2.35.zernike.2.
5433	T2.35.zernike.3.
5434	T2.35.zernike.4.
5435	T2.35.zernike.5.
5436	T2.35.zernike.6.
5437	T2.35.zernike.7.
5438	T2.35.zernike.8.
5439	T2.35.zernike.9.
5440	T2.35.zernike.10.
5441	T2.35.zernike.11.
5442	T2.35.zernike.12.
5443	T2.35.zernike.13.
5444	T2.35.zernike.14.
5445	T2.35.zernike.15.
5446	T2.35.zernike.16.
5447	T2.35.zernike.17.
5448	T2.35.zernike.18.
5449	T2.35.zernike.19.
5450	T2.35.zernike.20.
5451	T2.35.zernike.21.

5452	T2.35.zernike.22.
5453	T2.35.zernike.23.
5454	T2.35.zernike.24.
5455	T2.3.sobel
5456	T2.3.sobel_mask
5457	T2.all.stats.p000.
5458	T2.all.stats.p010.
5459	T2.all.stats.p020.
5460	T2.all.stats.p025.
5461	T2.all.stats.p030.
5462	T2.all.stats.p040.
5463	T2.all.stats.p050.
5464	T2.all.stats.p060.
5465	T2.all.stats.p070.
5466	T2.all.stats.p075.
5467	T2.all.stats.p080.
5468	T2.all.stats.p090.
5469	T2.all.stats.p100.
5470	T2.all.stats.range.
5471	T2.all.stats.mean.
5472	T2.all.stats.stddev.
5473	T2.all.stats.kurtosis.
5474	T2.all.stats.skewness.
5475	T2W.3.glcm.contrast.1.mean.
5476	T2W.3.glcm.contrast.1.range.
5477	T2W.3.glcm.contrast.2.mean.
5478	T2W.3.glcm.contrast.2.range.
5479	T2W.3.glcm.contrast.3.mean.
5480	T2W.3.glcm.contrast.3.range.
5481	T2W.3.glcm.dissimilarity.1.mean.
5482	T2W.3.glcm.dissimilarity.1.range.
5483	T2W.3.glcm.dissimilarity.2.mean.
5484	T2W.3.glcm.dissimilarity.2.range.
5485	T2W.3.glcm.dissimilarity.3.mean.
5486	T2W.3.glcm.dissimilarity.3.range.
5487	T2W.3.glcm.homogeneity.1.mean.
5488	T2W.3.glcm.homogeneity.1.range.

5489	T2W.3.glcm.homogeneity.2.mean.
5490	T2W.3.glcm.homogeneity.2.range.
5491	T2W.3.glcm.homogeneity.3.mean.
5492	T2W.3.glcm.homogeneity.3.range.
5493	T2W.3.glcm.energy.1.mean.
5494	T2W.3.glcm.energy.1.range.
5495	T2W.3.glcm.energy.2.mean.
5496	T2W.3.glcm.energy.2.range.
5497	T2W.3.glcm.energy.3.mean.
5498	T2W.3.glcm.energy.3.range.
5499	T2W.3.glcm.correlation.1.mean.
5500	T2W.3.glcm.correlation.1.range.
5501	T2W.3.glcm.correlation.2.mean.
5502	T2W.3.glcm.correlation.2.range.
5503	T2W.3.glcm.correlation.3.mean.
5504	T2W.3.glcm.correlation.3.range.
5505	T2W.3.glcm.ASM.1.mean.
5506	T2W.3.glcm.ASM.1.range.
5507	T2W.3.glcm.ASM.2.mean.
5508	T2W.3.glcm.ASM.2.range.
5509	T2W.3.glcm.ASM.3.mean.
5510	T2W.3.glcm.ASM.3.range.
5511	T2W.7.glcm.contrast.1.mean.
5512	T2W.7.glcm.contrast.1.range.
5513	T2W.7.glcm.contrast.2.mean.
5514	T2W.7.glcm.contrast.2.range.
5515	T2W.7.glcm.contrast.3.mean.
5516	T2W.7.glcm.contrast.3.range.
5517	T2W.7.glcm.contrast.4.mean.
5518	T2W.7.glcm.contrast.4.range.
5519	T2W.7.glcm.dissimilarity.1.mean.
5520	T2W.7.glcm.dissimilarity.1.range.
5521	T2W.7.glcm.dissimilarity.2.mean.
5522	T2W.7.glcm.dissimilarity.2.range.
5523	T2W.7.glcm.dissimilarity.3.mean.
5524	T2W.7.glcm.dissimilarity.3.range.
5525	T2W.7.glcm.dissimilarity.4.mean.

5526	T2W.7.glcm.dissimilarity.4.range.
5527	T2W.7.glcm.homogeneity.1.mean.
5528	T2W.7.glcm.homogeneity.1.range.
5529	T2W.7.glcm.homogeneity.2.mean.
5530	T2W.7.glcm.homogeneity.2.range.
5531	T2W.7.glcm.homogeneity.3.mean.
5532	T2W.7.glcm.homogeneity.3.range.
5533	T2W.7.glcm.homogeneity.4.mean.
5534	T2W.7.glcm.homogeneity.4.range.
5535	T2W.7.glcm.energy.1.mean.
5536	T2W.7.glcm.energy.1.range.
5537	T2W.7.glcm.energy.2.mean.
5538	T2W.7.glcm.energy.2.range.
5539	T2W.7.glcm.energy.3.mean.
5540	T2W.7.glcm.energy.3.range.
5541	T2W.7.glcm.energy.4.mean.
5542	T2W.7.glcm.energy.4.range.
5543	T2W.7.glcm.correlation.1.mean.
5544	T2W.7.glcm.correlation.1.range.
5545	T2W.7.glcm.correlation.2.mean.
5546	T2W.7.glcm.correlation.2.range.
5547	T2W.7.glcm.correlation.3.mean.
5548	T2W.7.glcm.correlation.3.range.
5549	T2W.7.glcm.correlation.4.mean.
5550	T2W.7.glcm.correlation.4.range.
5551	T2W.7.glcm.ASM.1.mean.
5552	T2W.7.glcm.ASM.1.range.
5553	T2W.7.glcm.ASM.2.mean.
5554	T2W.7.glcm.ASM.2.range.
5555	T2W.7.glcm.ASM.3.mean.
5556	T2W.7.glcm.ASM.3.range.
5557	T2W.7.glcm.ASM.4.mean.
5558	T2W.7.glcm.ASM.4.range.
5559	T2W.11.glcm.contrast.1.mean.
5560	T2W.11.glcm.contrast.1.range.
5561	T2W.11.glcm.contrast.2.mean.
5562	T2W.11.glcm.contrast.2.range.

5563	T2W.11.glcm.contrast.3.mean.
5564	T2W.11.glcm.contrast.3.range.
5565	T2W.11.glcm.contrast.4.mean.
5566	T2W.11.glcm.contrast.4.range.
5567	T2W.11.glcm.dissimilarity.1.mean.
5568	T2W.11.glcm.dissimilarity.1.range.
5569	T2W.11.glcm.dissimilarity.2.mean.
5570	T2W.11.glcm.dissimilarity.2.range.
5571	T2W.11.glcm.dissimilarity.3.mean.
5572	T2W.11.glcm.dissimilarity.3.range.
5573	T2W.11.glcm.dissimilarity.4.mean.
5574	T2W.11.glcm.dissimilarity.4.range.
5575	T2W.11.glcm.homogeneity.1.mean.
5576	T2W.11.glcm.homogeneity.1.range.
5577	T2W.11.glcm.homogeneity.2.mean.
5578	T2W.11.glcm.homogeneity.2.range.
5579	T2W.11.glcm.homogeneity.3.mean.
5580	T2W.11.glcm.homogeneity.3.range.
5581	T2W.11.glcm.homogeneity.4.mean.
5582	T2W.11.glcm.homogeneity.4.range.
5583	T2W.11.glcm.energy.1.mean.
5584	T2W.11.glcm.energy.1.range.
5585	T2W.11.glcm.energy.2.mean.
5586	T2W.11.glcm.energy.2.range.
5587	T2W.11.glcm.energy.3.mean.
5588	T2W.11.glcm.energy.3.range.
5589	T2W.11.glcm.energy.4.mean.
5590	T2W.11.glcm.energy.4.range.
5591	T2W.11.glcm.correlation.1.mean.
5592	T2W.11.glcm.correlation.1.range.
5593	T2W.11.glcm.correlation.2.mean.
5594	T2W.11.glcm.correlation.2.range.
5595	T2W.11.glcm.correlation.3.mean.
5596	T2W.11.glcm.correlation.3.range.
5597	T2W.11.glcm.correlation.4.mean.
5598	T2W.11.glcm.correlation.4.range.
5599	T2W.11.glcm.ASM.1.mean.

5600	T2W.11.glcm.ASM.1.range.
5601	T2W.11.glcm.ASM.2.mean.
5602	T2W.11.glcm.ASM.2.range.
5603	T2W.11.glcm.ASM.3.mean.
5604	T2W.11.glcm.ASM.3.range.
5605	T2W.11.glcm.ASM.4.mean.
5606	T2W.11.glcm.ASM.4.range.
5607	T2W.15.glcm.contrast.1.mean.
5608	T2W.15.glcm.contrast.1.range.
5609	T2W.15.glcm.contrast.2.mean.
5610	T2W.15.glcm.contrast.2.range.
5611	T2W.15.glcm.contrast.3.mean.
5612	T2W.15.glcm.contrast.3.range.
5613	T2W.15.glcm.contrast.4.mean.
5614	T2W.15.glcm.contrast.4.range.
5615	T2W.15.glcm.dissimilarity.1.mean.
5616	T2W.15.glcm.dissimilarity.1.range.
5617	T2W.15.glcm.dissimilarity.2.mean.
5618	T2W.15.glcm.dissimilarity.2.range.
5619	T2W.15.glcm.dissimilarity.3.mean.
5620	T2W.15.glcm.dissimilarity.3.range.
5621	T2W.15.glcm.dissimilarity.4.mean.
5622	T2W.15.glcm.dissimilarity.4.range.
5623	T2W.15.glcm.homogeneity.1.mean.
5624	T2W.15.glcm.homogeneity.1.range.
5625	T2W.15.glcm.homogeneity.2.mean.
5626	T2W.15.glcm.homogeneity.2.range.
5627	T2W.15.glcm.homogeneity.3.mean.
5628	T2W.15.glcm.homogeneity.3.range.
5629	T2W.15.glcm.homogeneity.4.mean.
5630	T2W.15.glcm.homogeneity.4.range.
5631	T2W.15.glcm.energy.1.mean.
5632	T2W.15.glcm.energy.1.range.
5633	T2W.15.glcm.energy.2.mean.
5634	T2W.15.glcm.energy.2.range.
5635	T2W.15.glcm.energy.3.mean.
5636	T2W.15.glcm.energy.3.range.

5637	T2W.15.glcm.energy.4.mean.
5638	T2W.15.glcm.energy.4.range.
5639	T2W.15.glcm.correlation.1.mean.
5640	T2W.15.glcm.correlation.1.range.
5641	T2W.15.glcm.correlation.2.mean.
5642	T2W.15.glcm.correlation.2.range.
5643	T2W.15.glcm.correlation.3.mean.
5644	T2W.15.glcm.correlation.3.range.
5645	T2W.15.glcm.correlation.4.mean.
5646	T2W.15.glcm.correlation.4.range.
5647	T2W.15.glcm.ASM.1.mean.
5648	T2W.15.glcm.ASM.1.range.
5649	T2W.15.glcm.ASM.2.mean.
5650	T2W.15.glcm.ASM.2.range.
5651	T2W.15.glcm.ASM.3.mean.
5652	T2W.15.glcm.ASM.3.range.
5653	T2W.15.glcm.ASM.4.mean.
5654	T2W.15.glcm.ASM.4.range.
5655	T2W.19.glcm.contrast.1.mean.
5656	T2W.19.glcm.contrast.1.range.
5657	T2W.19.glcm.contrast.2.mean.
5658	T2W.19.glcm.contrast.2.range.
5659	T2W.19.glcm.contrast.3.mean.
5660	T2W.19.glcm.contrast.3.range.
5661	T2W.19.glcm.contrast.4.mean.
5662	T2W.19.glcm.contrast.4.range.
5663	T2W.19.glcm.dissimilarity.1.mean.
5664	T2W.19.glcm.dissimilarity.1.range.
5665	T2W.19.glcm.dissimilarity.2.mean.
5666	T2W.19.glcm.dissimilarity.2.range.
5667	T2W.19.glcm.dissimilarity.3.mean.
5668	T2W.19.glcm.dissimilarity.3.range.
5669	T2W.19.glcm.dissimilarity.4.mean.
5670	T2W.19.glcm.dissimilarity.4.range.
5671	T2W.19.glcm.homogeneity.1.mean.
5672	T2W.19.glcm.homogeneity.1.range.
5673	T2W.19.glcm.homogeneity.2.mean.

5674	T2W.19.glcm.homogeneity.2.range.
5675	T2W.19.glcm.homogeneity.3.mean.
5676	T2W.19.glcm.homogeneity.3.range.
5677	T2W.19.glcm.homogeneity.4.mean.
5678	T2W.19.glcm.homogeneity.4.range.
5679	T2W.19.glcm.energy.1.mean.
5680	T2W.19.glcm.energy.1.range.
5681	T2W.19.glcm.energy.2.mean.
5682	T2W.19.glcm.energy.2.range.
5683	T2W.19.glcm.energy.3.mean.
5684	T2W.19.glcm.energy.3.range.
5685	T2W.19.glcm.energy.4.mean.
5686	T2W.19.glcm.energy.4.range.
5687	T2W.19.glcm.correlation.1.mean.
5688	T2W.19.glcm.correlation.1.range.
5689	T2W.19.glcm.correlation.2.mean.
5690	T2W.19.glcm.correlation.2.range.
5691	T2W.19.glcm.correlation.3.mean.
5692	T2W.19.glcm.correlation.3.range.
5693	T2W.19.glcm.correlation.4.mean.
5694	T2W.19.glcm.correlation.4.range.
5695	T2W.19.glcm.ASM.1.mean.
5696	T2W.19.glcm.ASM.1.range.
5697	T2W.19.glcm.ASM.2.mean.
5698	T2W.19.glcm.ASM.2.range.
5699	T2W.19.glcm.ASM.3.mean.
5700	T2W.19.glcm.ASM.3.range.
5701	T2W.19.glcm.ASM.4.mean.
5702	T2W.19.glcm.ASM.4.range.
5703	T2W.23.glcm.contrast.1.mean.
5704	T2W.23.glcm.contrast.1.range.
5705	T2W.23.glcm.contrast.2.mean.
5706	T2W.23.glcm.contrast.2.range.
5707	T2W.23.glcm.contrast.3.mean.
5708	T2W.23.glcm.contrast.3.range.
5709	T2W.23.glcm.contrast.4.mean.
5710	T2W.23.glcm.contrast.4.range.

5711	T2W.23.glcm.dissimilarity.1.mean.
5712	T2W.23.glcm.dissimilarity.1.range.
5713	T2W.23.glcm.dissimilarity.2.mean.
5714	T2W.23.glcm.dissimilarity.2.range.
5715	T2W.23.glcm.dissimilarity.3.mean.
5716	T2W.23.glcm.dissimilarity.3.range.
5717	T2W.23.glcm.dissimilarity.4.mean.
5718	T2W.23.glcm.dissimilarity.4.range.
5719	T2W.23.glcm.homogeneity.1.mean.
5720	T2W.23.glcm.homogeneity.1.range.
5721	T2W.23.glcm.homogeneity.2.mean.
5722	T2W.23.glcm.homogeneity.2.range.
5723	T2W.23.glcm.homogeneity.3.mean.
5724	T2W.23.glcm.homogeneity.3.range.
5725	T2W.23.glcm.homogeneity.4.mean.
5726	T2W.23.glcm.homogeneity.4.range.
5727	T2W.23.glcm.energy.1.mean.
5728	T2W.23.glcm.energy.1.range.
5729	T2W.23.glcm.energy.2.mean.
5730	T2W.23.glcm.energy.2.range.
5731	T2W.23.glcm.energy.3.mean.
5732	T2W.23.glcm.energy.3.range.
5733	T2W.23.glcm.energy.4.mean.
5734	T2W.23.glcm.energy.4.range.
5735	T2W.23.glcm.correlation.1.mean.
5736	T2W.23.glcm.correlation.1.range.
5737	T2W.23.glcm.correlation.2.mean.
5738	T2W.23.glcm.correlation.2.range.
5739	T2W.23.glcm.correlation.3.mean.
5740	T2W.23.glcm.correlation.3.range.
5741	T2W.23.glcm.correlation.4.mean.
5742	T2W.23.glcm.correlation.4.range.
5743	T2W.23.glcm.ASM.1.mean.
5744	T2W.23.glcm.ASM.1.range.
5745	T2W.23.glcm.ASM.2.mean.
5746	T2W.23.glcm.ASM.2.range.
5747	T2W.23.glcm.ASM.3.mean.

5748	T2W.23.glcm.ASM.3.range.
5749	T2W.23.glcm.ASM.4.mean.
5750	T2W.23.glcm.ASM.4.range.
5751	T2W.27.glcm.contrast.1.mean.
5752	T2W.27.glcm.contrast.1.range.
5753	T2W.27.glcm.contrast.2.mean.
5754	T2W.27.glcm.contrast.2.range.
5755	T2W.27.glcm.contrast.3.mean.
5756	T2W.27.glcm.contrast.3.range.
5757	T2W.27.glcm.contrast.4.mean.
5758	T2W.27.glcm.contrast.4.range.
5759	T2W.27.glcm.dissimilarity.1.mean.
5760	T2W.27.glcm.dissimilarity.1.range.
5761	T2W.27.glcm.dissimilarity.2.mean.
5762	T2W.27.glcm.dissimilarity.2.range.
5763	T2W.27.glcm.dissimilarity.3.mean.
5764	T2W.27.glcm.dissimilarity.3.range.
5765	T2W.27.glcm.dissimilarity.4.mean.
5766	T2W.27.glcm.dissimilarity.4.range.
5767	T2W.27.glcm.homogeneity.1.mean.
5768	T2W.27.glcm.homogeneity.1.range.
5769	T2W.27.glcm.homogeneity.2.mean.
5770	T2W.27.glcm.homogeneity.2.range.
5771	T2W.27.glcm.homogeneity.3.mean.
5772	T2W.27.glcm.homogeneity.3.range.
5773	T2W.27.glcm.homogeneity.4.mean.
5774	T2W.27.glcm.homogeneity.4.range.
5775	T2W.27.glcm.energy.1.mean.
5776	T2W.27.glcm.energy.1.range.
5777	T2W.27.glcm.energy.2.mean.
5778	T2W.27.glcm.energy.2.range.
5779	T2W.27.glcm.energy.3.mean.
5780	T2W.27.glcm.energy.3.range.
5781	T2W.27.glcm.energy.4.mean.
5782	T2W.27.glcm.energy.4.range.
5783	T2W.27.glcm.correlation.1.mean.
5784	T2W.27.glcm.correlation.1.range.

5785	T2W.27.glcm.correlation.2.mean.
5786	T2W.27.glcm.correlation.2.range.
5787	T2W.27.glcm.correlation.3.mean.
5788	T2W.27.glcm.correlation.3.range.
5789	T2W.27.glcm.correlation.4.mean.
5790	T2W.27.glcm.correlation.4.range.
5791	T2W.27.glcm.ASM.1.mean.
5792	T2W.27.glcm.ASM.1.range.
5793	T2W.27.glcm.ASM.2.mean.
5794	T2W.27.glcm.ASM.2.range.
5795	T2W.27.glcm.ASM.3.mean.
5796	T2W.27.glcm.ASM.3.range.
5797	T2W.27.glcm.ASM.4.mean.
5798	T2W.27.glcm.ASM.4.range.
5799	T2W.31.glcm.contrast.1.mean.
5800	T2W.31.glcm.contrast.1.range.
5801	T2W.31.glcm.contrast.2.mean.
5802	T2W.31.glcm.contrast.2.range.
5803	T2W.31.glcm.contrast.3.mean.
5804	T2W.31.glcm.contrast.3.range.
5805	T2W.31.glcm.contrast.4.mean.
5806	T2W.31.glcm.contrast.4.range.
5807	T2W.31.glcm.dissimilarity.1.mean.
5808	T2W.31.glcm.dissimilarity.1.range.
5809	T2W.31.glcm.dissimilarity.2.mean.
5810	T2W.31.glcm.dissimilarity.2.range.
5811	T2W.31.glcm.dissimilarity.3.mean.
5812	T2W.31.glcm.dissimilarity.3.range.
5813	T2W.31.glcm.dissimilarity.4.mean.
5814	T2W.31.glcm.dissimilarity.4.range.
5815	T2W.31.glcm.homogeneity.1.mean.
5816	T2W.31.glcm.homogeneity.1.range.
5817	T2W.31.glcm.homogeneity.2.mean.
5818	T2W.31.glcm.homogeneity.2.range.
5819	T2W.31.glcm.homogeneity.3.mean.
5820	T2W.31.glcm.homogeneity.3.range.
5821	T2W.31.glcm.homogeneity.4.mean.

5822	T2W.31.glcm.homogeneity.4.range.
5823	T2W.31.glcm.energy.1.mean.
5824	T2W.31.glcm.energy.1.range.
5825	T2W.31.glcm.energy.2.mean.
5826	T2W.31.glcm.energy.2.range.
5827	T2W.31.glcm.energy.3.mean.
5828	T2W.31.glcm.energy.3.range.
5829	T2W.31.glcm.energy.4.mean.
5830	T2W.31.glcm.energy.4.range.
5831	T2W.31.glcm.correlation.1.mean.
5832	T2W.31.glcm.correlation.1.range.
5833	T2W.31.glcm.correlation.2.mean.
5834	T2W.31.glcm.correlation.2.range.
5835	T2W.31.glcm.correlation.3.mean.
5836	T2W.31.glcm.correlation.3.range.
5837	T2W.31.glcm.correlation.4.mean.
5838	T2W.31.glcm.correlation.4.range.
5839	T2W.31.glcm.ASM.1.mean.
5840	T2W.31.glcm.ASM.1.range.
5841	T2W.31.glcm.ASM.2.mean.
5842	T2W.31.glcm.ASM.2.range.
5843	T2W.31.glcm.ASM.3.mean.
5844	T2W.31.glcm.ASM.3.range.
5845	T2W.31.glcm.ASM.4.mean.
5846	T2W.31.glcm.ASM.4.range.
5847	T2W.35.glcm.contrast.1.mean.
5848	T2W.35.glcm.contrast.1.range.
5849	T2W.35.glcm.contrast.2.mean.
5850	T2W.35.glcm.contrast.2.range.
5851	T2W.35.glcm.contrast.3.mean.
5852	T2W.35.glcm.contrast.3.range.
5853	T2W.35.glcm.contrast.4.mean.
5854	T2W.35.glcm.contrast.4.range.
5855	T2W.35.glcm.dissimilarity.1.mean.
5856	T2W.35.glcm.dissimilarity.1.range.
5857	T2W.35.glcm.dissimilarity.2.mean.
5858	T2W.35.glcm.dissimilarity.2.range.

5859	T2W.35.glcm.dissimilarity.3.mean.
5860	T2W.35.glcm.dissimilarity.3.range.
5861	T2W.35.glcm.dissimilarity.4.mean.
5862	T2W.35.glcm.dissimilarity.4.range.
5863	T2W.35.glcm.homogeneity.1.mean.
5864	T2W.35.glcm.homogeneity.1.range.
5865	T2W.35.glcm.homogeneity.2.mean.
5866	T2W.35.glcm.homogeneity.2.range.
5867	T2W.35.glcm.homogeneity.3.mean.
5868	T2W.35.glcm.homogeneity.3.range.
5869	T2W.35.glcm.homogeneity.4.mean.
5870	T2W.35.glcm.homogeneity.4.range.
5871	T2W.35.glcm.energy.1.mean.
5872	T2W.35.glcm.energy.1.range.
5873	T2W.35.glcm.energy.2.mean.
5874	T2W.35.glcm.energy.2.range.
5875	T2W.35.glcm.energy.3.mean.
5876	T2W.35.glcm.energy.3.range.
5877	T2W.35.glcm.energy.4.mean.
5878	T2W.35.glcm.energy.4.range.
5879	T2W.35.glcm.correlation.1.mean.
5880	T2W.35.glcm.correlation.1.range.
5881	T2W.35.glcm.correlation.2.mean.
5882	T2W.35.glcm.correlation.2.range.
5883	T2W.35.glcm.correlation.3.mean.
5884	T2W.35.glcm.correlation.3.range.
5885	T2W.35.glcm.correlation.4.mean.
5886	T2W.35.glcm.correlation.4.range.
5887	T2W.35.glcm.ASM.1.mean.
5888	T2W.35.glcm.ASM.1.range.
5889	T2W.35.glcm.ASM.2.mean.
5890	T2W.35.glcm.ASM.2.range.
5891	T2W.35.glcm.ASM.3.mean.
5892	T2W.35.glcm.ASM.3.range.
5893	T2W.35.glcm.ASM.4.mean.
5894	T2W.35.glcm.ASM.4.range.
5895	T2W.mbb.glcm.contrast.1.mean.

5896	T2W.mbb.glcm.contrast.1.range.
5897	T2W.mbb.glcm.contrast.2.mean.
5898	T2W.mbb.glcm.contrast.2.range.
5899	T2W.mbb.glcm.contrast.3.mean.
5900	T2W.mbb.glcm.contrast.3.range.
5901	T2W.mbb.glcm.contrast.4.mean.
5902	T2W.mbb.glcm.contrast.4.range.
5903	T2W.mbb.glcm.dissimilarity.1.mean.
5904	T2W.mbb.glcm.dissimilarity.1.range.
5905	T2W.mbb.glcm.dissimilarity.2.mean.
5906	T2W.mbb.glcm.dissimilarity.2.range.
5907	T2W.mbb.glcm.dissimilarity.3.mean.
5908	T2W.mbb.glcm.dissimilarity.3.range.
5909	T2W.mbb.glcm.dissimilarity.4.mean.
5910	T2W.mbb.glcm.dissimilarity.4.range.
5911	T2W.mbb.glcm.homogeneity.1.mean.
5912	T2W.mbb.glcm.homogeneity.1.range.
5913	T2W.mbb.glcm.homogeneity.2.mean.
5914	T2W.mbb.glcm.homogeneity.2.range.
5915	T2W.mbb.glcm.homogeneity.3.mean.
5916	T2W.mbb.glcm.homogeneity.3.range.
5917	T2W.mbb.glcm.homogeneity.4.mean.
5918	T2W.mbb.glcm.homogeneity.4.range.
5919	T2W.mbb.glcm.energy.1.mean.
5920	T2W.mbb.glcm.energy.1.range.
5921	T2W.mbb.glcm.energy.2.mean.
5922	T2W.mbb.glcm.energy.2.range.
5923	T2W.mbb.glcm.energy.3.mean.
5924	T2W.mbb.glcm.energy.3.range.
5925	T2W.mbb.glcm.energy.4.mean.
5926	T2W.mbb.glcm.energy.4.range.
5927	T2W.mbb.glcm.correlation.1.mean.
5928	T2W.mbb.glcm.correlation.1.range.
5929	T2W.mbb.glcm.correlation.2.mean.
5930	T2W.mbb.glcm.correlation.2.range.
5931	T2W.mbb.glcm.correlation.3.mean.
5932	T2W.mbb.glcm.correlation.3.range.

5933	T2W.mbb.glcm.correlation.4.mean.
5934	T2W.mbb.glcm.correlation.4.range.
5935	T2W.mbb.glcm.ASM.1.mean.
5936	T2W.mbb.glcm.ASM.1.range.
5937	T2W.mbb.glcm.ASM.2.mean.
5938	T2W.mbb.glcm.ASM.2.range.
5939	T2W.mbb.glcm.ASM.3.mean.
5940	T2W.mbb.glcm.ASM.3.range.
5941	T2W.mbb.glcm.ASM.4.mean.
5942	T2W.mbb.glcm.ASM.4.range.
5943	T2W.3.lbp.1.0.
5944	T2W.3.lbp.1.1.
5945	T2W.3.lbp.1.2.
5946	T2W.3.lbp.1.3.
5947	T2W.3.lbp.1.4.
5948	T2W.3.lbp.1.5.
5949	T2W.3.lbp.1.6.
5950	T2W.3.lbp.1.7.
5951	T2W.3.lbp.1.8.
5952	T2W.3.lbp.1.9.
5953	T2W.7.lbp.3.0.
5954	T2W.7.lbp.3.1.
5955	T2W.7.lbp.3.2.
5956	T2W.7.lbp.3.3.
5957	T2W.7.lbp.3.4.
5958	T2W.7.lbp.3.5.
5959	T2W.7.lbp.3.6.
5960	T2W.7.lbp.3.7.
5961	T2W.7.lbp.3.8.
5962	T2W.7.lbp.3.9.
5963	T2W.11.lbp.5.0.
5964	T2W.11.lbp.5.1.
5965	T2W.11.lbp.5.2.
5966	T2W.11.lbp.5.3.
5967	T2W.11.lbp.5.4.
5968	T2W.11.lbp.5.5.
5969	T2W.11.lbp.5.6.

5970	T2W.11.lbp.5.7.
5971	T2W.11.lbp.5.8.
5972	T2W.11.lbp.5.9.
5973	T2W.15.lbp.7.0.
5974	T2W.15.lbp.7.1.
5975	T2W.15.lbp.7.2.
5976	T2W.15.lbp.7.3.
5977	T2W.15.lbp.7.4.
5978	T2W.15.lbp.7.5.
5979	T2W.15.lbp.7.6.
5980	T2W.15.lbp.7.7.
5981	T2W.15.lbp.7.8.
5982	T2W.15.lbp.7.9.
5983	T2W.19.lbp.9.0.
5984	T2W.19.lbp.9.1.
5985	T2W.19.lbp.9.2.
5986	T2W.19.lbp.9.3.
5987	T2W.19.lbp.9.4.
5988	T2W.19.lbp.9.5.
5989	T2W.19.lbp.9.6.
5990	T2W.19.lbp.9.7.
5991	T2W.19.lbp.9.8.
5992	T2W.19.lbp.9.9.
5993	T2W.23.lbp.11.0.
5994	T2W.23.lbp.11.1.
5995	T2W.23.lbp.11.2.
5996	T2W.23.lbp.11.3.
5997	T2W.23.lbp.11.4.
5998	T2W.23.lbp.11.5.
5999	T2W.23.lbp.11.6.
6000	T2W.23.lbp.11.7.
6001	T2W.23.lbp.11.8.
6002	T2W.23.lbp.11.9.
6003	T2W.27.lbp.13.0.
6004	T2W.27.lbp.13.1.
6005	T2W.27.lbp.13.2.
6006	T2W.27.lbp.13.3.

6007	T2W.27.lbp.13.4.
6008	T2W.27.lbp.13.5.
6009	T2W.27.lbp.13.6.
6010	T2W.27.lbp.13.7.
6011	T2W.27.lbp.13.8.
6012	T2W.27.lbp.13.9.
6013	T2W.31.lbp.15.0.
6014	T2W.31.lbp.15.1.
6015	T2W.31.lbp.15.2.
6016	T2W.31.lbp.15.3.
6017	T2W.31.lbp.15.4.
6018	T2W.31.lbp.15.5.
6019	T2W.31.lbp.15.6.
6020	T2W.31.lbp.15.7.
6021	T2W.31.lbp.15.8.
6022	T2W.31.lbp.15.9.
6023	T2W.35.lbp.17.0.
6024	T2W.35.lbp.17.1.
6025	T2W.35.lbp.17.2.
6026	T2W.35.lbp.17.3.
6027	T2W.35.lbp.17.4.
6028	T2W.35.lbp.17.5.
6029	T2W.35.lbp.17.6.
6030	T2W.35.lbp.17.7.
6031	T2W.35.lbp.17.8.
6032	T2W.35.lbp.17.9.
6033	T2W.3.hog
6034	T2W.7.hog
6035	T2W.11.hog
6036	T2W.15.hog
6037	T2W.19.hog
6038	T2W.23.hog
6039	T2W.27.hog
6040	T2W.31.hog
6041	T2W.35.hog
6042	T2W.3.gabor.1.0.1.mean.
6043	T2W.3.gabor.1.0.1.var.

6044	T2W.3.gabor.1.0.1.absmean.
6045	T2W.3.gabor.1.0.1.mag.
6046	T2W.3.gabor.1.0.2.mean.
6047	T2W.3.gabor.1.0.2.var.
6048	T2W.3.gabor.1.0.2.absmean.
6049	T2W.3.gabor.1.0.2.mag.
6050	T2W.3.gabor.1.0.3.mean.
6051	T2W.3.gabor.1.0.3.var.
6052	T2W.3.gabor.1.0.3.absmean.
6053	T2W.3.gabor.1.0.3.mag.
6054	T2W.3.gabor.1.0.4.mean.
6055	T2W.3.gabor.1.0.4.var.
6056	T2W.3.gabor.1.0.4.absmean.
6057	T2W.3.gabor.1.0.4.mag.
6058	T2W.3.gabor.1.0.5.mean.
6059	T2W.3.gabor.1.0.5.var.
6060	T2W.3.gabor.1.0.5.absmean.
6061	T2W.3.gabor.1.0.5.mag.
6062	T2W.3.gabor.2.0.1.mean.
6063	T2W.3.gabor.2.0.1.var.
6064	T2W.3.gabor.2.0.1.absmean.
6065	T2W.3.gabor.2.0.1.mag.
6066	T2W.3.gabor.2.0.2.mean.
6067	T2W.3.gabor.2.0.2.var.
6068	T2W.3.gabor.2.0.2.absmean.
6069	T2W.3.gabor.2.0.2.mag.
6070	T2W.3.gabor.2.0.3.mean.
6071	T2W.3.gabor.2.0.3.var.
6072	T2W.3.gabor.2.0.3.absmean.
6073	T2W.3.gabor.2.0.3.mag.
6074	T2W.3.gabor.2.0.4.mean.
6075	T2W.3.gabor.2.0.4.var.
6076	T2W.3.gabor.2.0.4.absmean.
6077	T2W.3.gabor.2.0.4.mag.
6078	T2W.3.gabor.2.0.5.mean.
6079	T2W.3.gabor.2.0.5.var.
6080	T2W.3.gabor.2.0.5.absmean.

6081	T2W.3.gabor.2.0.5.mag.
6082	T2W.3.gabor.3.0.1.mean.
6083	T2W.3.gabor.3.0.1.var.
6084	T2W.3.gabor.3.0.1.absmean.
6085	T2W.3.gabor.3.0.1.mag.
6086	T2W.3.gabor.3.0.2.mean.
6087	T2W.3.gabor.3.0.2.var.
6088	T2W.3.gabor.3.0.2.absmean.
6089	T2W.3.gabor.3.0.2.mag.
6090	T2W.3.gabor.3.0.3.mean.
6091	T2W.3.gabor.3.0.3.var.
6092	T2W.3.gabor.3.0.3.absmean.
6093	T2W.3.gabor.3.0.3.mag.
6094	T2W.3.gabor.3.0.4.mean.
6095	T2W.3.gabor.3.0.4.var.
6096	T2W.3.gabor.3.0.4.absmean.
6097	T2W.3.gabor.3.0.4.mag.
6098	T2W.3.gabor.3.0.5.mean.
6099	T2W.3.gabor.3.0.5.var.
6100	T2W.3.gabor.3.0.5.absmean.
6101	T2W.3.gabor.3.0.5.mag.
6102	T2W.7.gabor.1.0.1.mean.
6103	T2W.7.gabor.1.0.1.var.
6104	T2W.7.gabor.1.0.1.absmean.
6105	T2W.7.gabor.1.0.1.mag.
6106	T2W.7.gabor.1.0.2.mean.
6107	T2W.7.gabor.1.0.2.var.
6108	T2W.7.gabor.1.0.2.absmean.
6109	T2W.7.gabor.1.0.2.mag.
6110	T2W.7.gabor.1.0.3.mean.
6111	T2W.7.gabor.1.0.3.var.
6112	T2W.7.gabor.1.0.3.absmean.
6113	T2W.7.gabor.1.0.3.mag.
6114	T2W.7.gabor.1.0.4.mean.
6115	T2W.7.gabor.1.0.4.var.
6116	T2W.7.gabor.1.0.4.absmean.
6117	T2W.7.gabor.1.0.4.mag.

6118	T2W.7.gabor.1.0.5.mean.
6119	T2W.7.gabor.1.0.5.var.
6120	T2W.7.gabor.1.0.5.absmean.
6121	T2W.7.gabor.1.0.5.mag.
6122	T2W.7.gabor.2.0.1.mean.
6123	T2W.7.gabor.2.0.1.var.
6124	T2W.7.gabor.2.0.1.absmean.
6125	T2W.7.gabor.2.0.1.mag.
6126	T2W.7.gabor.2.0.2.mean.
6127	T2W.7.gabor.2.0.2.var.
6128	T2W.7.gabor.2.0.2.absmean.
6129	T2W.7.gabor.2.0.2.mag.
6130	T2W.7.gabor.2.0.3.mean.
6131	T2W.7.gabor.2.0.3.var.
6132	T2W.7.gabor.2.0.3.absmean.
6133	T2W.7.gabor.2.0.3.mag.
6134	T2W.7.gabor.2.0.4.mean.
6135	T2W.7.gabor.2.0.4.var.
6136	T2W.7.gabor.2.0.4.absmean.
6137	T2W.7.gabor.2.0.4.mag.
6138	T2W.7.gabor.2.0.5.mean.
6139	T2W.7.gabor.2.0.5.var.
6140	T2W.7.gabor.2.0.5.absmean.
6141	T2W.7.gabor.2.0.5.mag.
6142	T2W.7.gabor.3.0.1.mean.
6143	T2W.7.gabor.3.0.1.var.
6144	T2W.7.gabor.3.0.1.absmean.
6145	T2W.7.gabor.3.0.1.mag.
6146	T2W.7.gabor.3.0.2.mean.
6147	T2W.7.gabor.3.0.2.var.
6148	T2W.7.gabor.3.0.2.absmean.
6149	T2W.7.gabor.3.0.2.mag.
6150	T2W.7.gabor.3.0.3.mean.
6151	T2W.7.gabor.3.0.3.var.
6152	T2W.7.gabor.3.0.3.absmean.
6153	T2W.7.gabor.3.0.3.mag.
6154	T2W.7.gabor.3.0.4.mean.

6155	T2W.7.gabor.3.0.4.var.
6156	T2W.7.gabor.3.0.4.absmean.
6157	T2W.7.gabor.3.0.4.mag.
6158	T2W.7.gabor.3.0.5.mean.
6159	T2W.7.gabor.3.0.5.var.
6160	T2W.7.gabor.3.0.5.absmean.
6161	T2W.7.gabor.3.0.5.mag.
6162	T2W.11.gabor.1.0.1.mean.
6163	T2W.11.gabor.1.0.1.var.
6164	T2W.11.gabor.1.0.1.absmean.
6165	T2W.11.gabor.1.0.1.mag.
6166	T2W.11.gabor.1.0.2.mean.
6167	T2W.11.gabor.1.0.2.var.
6168	T2W.11.gabor.1.0.2.absmean.
6169	T2W.11.gabor.1.0.2.mag.
6170	T2W.11.gabor.1.0.3.mean.
6171	T2W.11.gabor.1.0.3.var.
6172	T2W.11.gabor.1.0.3.absmean.
6173	T2W.11.gabor.1.0.3.mag.
6174	T2W.11.gabor.1.0.4.mean.
6175	T2W.11.gabor.1.0.4.var.
6176	T2W.11.gabor.1.0.4.absmean.
6177	T2W.11.gabor.1.0.4.mag.
6178	T2W.11.gabor.1.0.5.mean.
6179	T2W.11.gabor.1.0.5.var.
6180	T2W.11.gabor.1.0.5.absmean.
6181	T2W.11.gabor.1.0.5.mag.
6182	T2W.11.gabor.2.0.1.mean.
6183	T2W.11.gabor.2.0.1.var.
6184	T2W.11.gabor.2.0.1.absmean.
6185	T2W.11.gabor.2.0.1.mag.
6186	T2W.11.gabor.2.0.2.mean.
6187	T2W.11.gabor.2.0.2.var.
6188	T2W.11.gabor.2.0.2.absmean.
6189	T2W.11.gabor.2.0.2.mag.
6190	T2W.11.gabor.2.0.3.mean.
6191	T2W.11.gabor.2.0.3.var.

6192	T2W.11.gabor.2.0.3.absmean.
6193	T2W.11.gabor.2.0.3.mag.
6194	T2W.11.gabor.2.0.4.mean.
6195	T2W.11.gabor.2.0.4.var.
6196	T2W.11.gabor.2.0.4.absmean.
6197	T2W.11.gabor.2.0.4.mag.
6198	T2W.11.gabor.2.0.5.mean.
6199	T2W.11.gabor.2.0.5.var.
6200	T2W.11.gabor.2.0.5.absmean.
6201	T2W.11.gabor.2.0.5.mag.
6202	T2W.11.gabor.3.0.1.mean.
6203	T2W.11.gabor.3.0.1.var.
6204	T2W.11.gabor.3.0.1.absmean.
6205	T2W.11.gabor.3.0.1.mag.
6206	T2W.11.gabor.3.0.2.mean.
6207	T2W.11.gabor.3.0.2.var.
6208	T2W.11.gabor.3.0.2.absmean.
6209	T2W.11.gabor.3.0.2.mag.
6210	T2W.11.gabor.3.0.3.mean.
6211	T2W.11.gabor.3.0.3.var.
6212	T2W.11.gabor.3.0.3.absmean.
6213	T2W.11.gabor.3.0.3.mag.
6214	T2W.11.gabor.3.0.4.mean.
6215	T2W.11.gabor.3.0.4.var.
6216	T2W.11.gabor.3.0.4.absmean.
6217	T2W.11.gabor.3.0.4.mag.
6218	T2W.11.gabor.3.0.5.mean.
6219	T2W.11.gabor.3.0.5.var.
6220	T2W.11.gabor.3.0.5.absmean.
6221	T2W.11.gabor.3.0.5.mag.
6222	T2W.15.gabor.1.0.1.mean.
6223	T2W.15.gabor.1.0.1.var.
6224	T2W.15.gabor.1.0.1.absmean.
6225	T2W.15.gabor.1.0.1.mag.
6226	T2W.15.gabor.1.0.2.mean.
6227	T2W.15.gabor.1.0.2.var.
6228	T2W.15.gabor.1.0.2.absmean.

6229	T2W.15.gabor.1.0.2.mag.
6230	T2W.15.gabor.1.0.3.mean.
6231	T2W.15.gabor.1.0.3.var.
6232	T2W.15.gabor.1.0.3.absmean.
6233	T2W.15.gabor.1.0.3.mag.
6234	T2W.15.gabor.1.0.4.mean.
6235	T2W.15.gabor.1.0.4.var.
6236	T2W.15.gabor.1.0.4.absmean.
6237	T2W.15.gabor.1.0.4.mag.
6238	T2W.15.gabor.1.0.5.mean.
6239	T2W.15.gabor.1.0.5.var.
6240	T2W.15.gabor.1.0.5.absmean.
6241	T2W.15.gabor.1.0.5.mag.
6242	T2W.15.gabor.2.0.1.mean.
6243	T2W.15.gabor.2.0.1.var.
6244	T2W.15.gabor.2.0.1.absmean.
6245	T2W.15.gabor.2.0.1.mag.
6246	T2W.15.gabor.2.0.2.mean.
6247	T2W.15.gabor.2.0.2.var.
6248	T2W.15.gabor.2.0.2.absmean.
6249	T2W.15.gabor.2.0.2.mag.
6250	T2W.15.gabor.2.0.3.mean.
6251	T2W.15.gabor.2.0.3.var.
6252	T2W.15.gabor.2.0.3.absmean.
6253	T2W.15.gabor.2.0.3.mag.
6254	T2W.15.gabor.2.0.4.mean.
6255	T2W.15.gabor.2.0.4.var.
6256	T2W.15.gabor.2.0.4.absmean.
6257	T2W.15.gabor.2.0.4.mag.
6258	T2W.15.gabor.2.0.5.mean.
6259	T2W.15.gabor.2.0.5.var.
6260	T2W.15.gabor.2.0.5.absmean.
6261	T2W.15.gabor.2.0.5.mag.
6262	T2W.15.gabor.3.0.1.mean.
6263	T2W.15.gabor.3.0.1.var.
6264	T2W.15.gabor.3.0.1.absmean.
6265	T2W.15.gabor.3.0.1.mag.

6266	T2W.15.gabor.3.0.2.mean.
6267	T2W.15.gabor.3.0.2.var.
6268	T2W.15.gabor.3.0.2.absmean.
6269	T2W.15.gabor.3.0.2.mag.
6270	T2W.15.gabor.3.0.3.mean.
6271	T2W.15.gabor.3.0.3.var.
6272	T2W.15.gabor.3.0.3.absmean.
6273	T2W.15.gabor.3.0.3.mag.
6274	T2W.15.gabor.3.0.4.mean.
6275	T2W.15.gabor.3.0.4.var.
6276	T2W.15.gabor.3.0.4.absmean.
6277	T2W.15.gabor.3.0.4.mag.
6278	T2W.15.gabor.3.0.5.mean.
6279	T2W.15.gabor.3.0.5.var.
6280	T2W.15.gabor.3.0.5.absmean.
6281	T2W.15.gabor.3.0.5.mag.
6282	T2W.19.gabor.1.0.1.mean.
6283	T2W.19.gabor.1.0.1.var.
6284	T2W.19.gabor.1.0.1.absmean.
6285	T2W.19.gabor.1.0.1.mag.
6286	T2W.19.gabor.1.0.2.mean.
6287	T2W.19.gabor.1.0.2.var.
6288	T2W.19.gabor.1.0.2.absmean.
6289	T2W.19.gabor.1.0.2.mag.
6290	T2W.19.gabor.1.0.3.mean.
6291	T2W.19.gabor.1.0.3.var.
6292	T2W.19.gabor.1.0.3.absmean.
6293	T2W.19.gabor.1.0.3.mag.
6294	T2W.19.gabor.1.0.4.mean.
6295	T2W.19.gabor.1.0.4.var.
6296	T2W.19.gabor.1.0.4.absmean.
6297	T2W.19.gabor.1.0.4.mag.
6298	T2W.19.gabor.1.0.5.mean.
6299	T2W.19.gabor.1.0.5.var.
6300	T2W.19.gabor.1.0.5.absmean.
6301	T2W.19.gabor.1.0.5.mag.
6302	T2W.19.gabor.2.0.1.mean.

6303	T2W.19.gabor.2.0.1.var.
6304	T2W.19.gabor.2.0.1.absmean.
6305	T2W.19.gabor.2.0.1.mag.
6306	T2W.19.gabor.2.0.2.mean.
6307	T2W.19.gabor.2.0.2.var.
6308	T2W.19.gabor.2.0.2.absmean.
6309	T2W.19.gabor.2.0.2.mag.
6310	T2W.19.gabor.2.0.3.mean.
6311	T2W.19.gabor.2.0.3.var.
6312	T2W.19.gabor.2.0.3.absmean.
6313	T2W.19.gabor.2.0.3.mag.
6314	T2W.19.gabor.2.0.4.mean.
6315	T2W.19.gabor.2.0.4.var.
6316	T2W.19.gabor.2.0.4.absmean.
6317	T2W.19.gabor.2.0.4.mag.
6318	T2W.19.gabor.2.0.5.mean.
6319	T2W.19.gabor.2.0.5.var.
6320	T2W.19.gabor.2.0.5.absmean.
6321	T2W.19.gabor.2.0.5.mag.
6322	T2W.19.gabor.3.0.1.mean.
6323	T2W.19.gabor.3.0.1.var.
6324	T2W.19.gabor.3.0.1.absmean.
6325	T2W.19.gabor.3.0.1.mag.
6326	T2W.19.gabor.3.0.2.mean.
6327	T2W.19.gabor.3.0.2.var.
6328	T2W.19.gabor.3.0.2.absmean.
6329	T2W.19.gabor.3.0.2.mag.
6330	T2W.19.gabor.3.0.3.mean.
6331	T2W.19.gabor.3.0.3.var.
6332	T2W.19.gabor.3.0.3.absmean.
6333	T2W.19.gabor.3.0.3.mag.
6334	T2W.19.gabor.3.0.4.mean.
6335	T2W.19.gabor.3.0.4.var.
6336	T2W.19.gabor.3.0.4.absmean.
6337	T2W.19.gabor.3.0.4.mag.
6338	T2W.19.gabor.3.0.5.mean.
6339	T2W.19.gabor.3.0.5.var.

6340	T2W.19.gabor.3.0.5.absmean.
6341	T2W.19.gabor.3.0.5.mag.
6342	T2W.23.gabor.1.0.1.mean.
6343	T2W.23.gabor.1.0.1.var.
6344	T2W.23.gabor.1.0.1.absmean.
6345	T2W.23.gabor.1.0.1.mag.
6346	T2W.23.gabor.1.0.2.mean.
6347	T2W.23.gabor.1.0.2.var.
6348	T2W.23.gabor.1.0.2.absmean.
6349	T2W.23.gabor.1.0.2.mag.
6350	T2W.23.gabor.1.0.3.mean.
6351	T2W.23.gabor.1.0.3.var.
6352	T2W.23.gabor.1.0.3.absmean.
6353	T2W.23.gabor.1.0.3.mag.
6354	T2W.23.gabor.1.0.4.mean.
6355	T2W.23.gabor.1.0.4.var.
6356	T2W.23.gabor.1.0.4.absmean.
6357	T2W.23.gabor.1.0.4.mag.
6358	T2W.23.gabor.1.0.5.mean.
6359	T2W.23.gabor.1.0.5.var.
6360	T2W.23.gabor.1.0.5.absmean.
6361	T2W.23.gabor.1.0.5.mag.
6362	T2W.23.gabor.2.0.1.mean.
6363	T2W.23.gabor.2.0.1.var.
6364	T2W.23.gabor.2.0.1.absmean.
6365	T2W.23.gabor.2.0.1.mag.
6366	T2W.23.gabor.2.0.2.mean.
6367	T2W.23.gabor.2.0.2.var.
6368	T2W.23.gabor.2.0.2.absmean.
6369	T2W.23.gabor.2.0.2.mag.
6370	T2W.23.gabor.2.0.3.mean.
6371	T2W.23.gabor.2.0.3.var.
6372	T2W.23.gabor.2.0.3.absmean.
6373	T2W.23.gabor.2.0.3.mag.
6374	T2W.23.gabor.2.0.4.mean.
6375	T2W.23.gabor.2.0.4.var.
6376	T2W.23.gabor.2.0.4.absmean.

6377	T2W.23.gabor.2.0.4.mag.
6378	T2W.23.gabor.2.0.5.mean.
6379	T2W.23.gabor.2.0.5.var.
6380	T2W.23.gabor.2.0.5.absmean.
6381	T2W.23.gabor.2.0.5.mag.
6382	T2W.23.gabor.3.0.1.mean.
6383	T2W.23.gabor.3.0.1.var.
6384	T2W.23.gabor.3.0.1.absmean.
6385	T2W.23.gabor.3.0.1.mag.
6386	T2W.23.gabor.3.0.2.mean.
6387	T2W.23.gabor.3.0.2.var.
6388	T2W.23.gabor.3.0.2.absmean.
6389	T2W.23.gabor.3.0.2.mag.
6390	T2W.23.gabor.3.0.3.mean.
6391	T2W.23.gabor.3.0.3.var.
6392	T2W.23.gabor.3.0.3.absmean.
6393	T2W.23.gabor.3.0.3.mag.
6394	T2W.23.gabor.3.0.4.mean.
6395	T2W.23.gabor.3.0.4.var.
6396	T2W.23.gabor.3.0.4.absmean.
6397	T2W.23.gabor.3.0.4.mag.
6398	T2W.23.gabor.3.0.5.mean.
6399	T2W.23.gabor.3.0.5.var.
6400	T2W.23.gabor.3.0.5.absmean.
6401	T2W.23.gabor.3.0.5.mag.
6402	T2W.27.gabor.1.0.1.mean.
6403	T2W.27.gabor.1.0.1.var.
6404	T2W.27.gabor.1.0.1.absmean.
6405	T2W.27.gabor.1.0.1.mag.
6406	T2W.27.gabor.1.0.2.mean.
6407	T2W.27.gabor.1.0.2.var.
6408	T2W.27.gabor.1.0.2.absmean.
6409	T2W.27.gabor.1.0.2.mag.
6410	T2W.27.gabor.1.0.3.mean.
6411	T2W.27.gabor.1.0.3.var.
6412	T2W.27.gabor.1.0.3.absmean.
6413	T2W.27.gabor.1.0.3.mag.

6414	T2W.27.gabor.1.0.4.mean.
6415	T2W.27.gabor.1.0.4.var.
6416	T2W.27.gabor.1.0.4.absmean.
6417	T2W.27.gabor.1.0.4.mag.
6418	T2W.27.gabor.1.0.5.mean.
6419	T2W.27.gabor.1.0.5.var.
6420	T2W.27.gabor.1.0.5.absmean.
6421	T2W.27.gabor.1.0.5.mag.
6422	T2W.27.gabor.2.0.1.mean.
6423	T2W.27.gabor.2.0.1.var.
6424	T2W.27.gabor.2.0.1.absmean.
6425	T2W.27.gabor.2.0.1.mag.
6426	T2W.27.gabor.2.0.2.mean.
6427	T2W.27.gabor.2.0.2.var.
6428	T2W.27.gabor.2.0.2.absmean.
6429	T2W.27.gabor.2.0.2.mag.
6430	T2W.27.gabor.2.0.3.mean.
6431	T2W.27.gabor.2.0.3.var.
6432	T2W.27.gabor.2.0.3.absmean.
6433	T2W.27.gabor.2.0.3.mag.
6434	T2W.27.gabor.2.0.4.mean.
6435	T2W.27.gabor.2.0.4.var.
6436	T2W.27.gabor.2.0.4.absmean.
6437	T2W.27.gabor.2.0.4.mag.
6438	T2W.27.gabor.2.0.5.mean.
6439	T2W.27.gabor.2.0.5.var.
6440	T2W.27.gabor.2.0.5.absmean.
6441	T2W.27.gabor.2.0.5.mag.
6442	T2W.27.gabor.3.0.1.mean.
6443	T2W.27.gabor.3.0.1.var.
6444	T2W.27.gabor.3.0.1.absmean.
6445	T2W.27.gabor.3.0.1.mag.
6446	T2W.27.gabor.3.0.2.mean.
6447	T2W.27.gabor.3.0.2.var.
6448	T2W.27.gabor.3.0.2.absmean.
6449	T2W.27.gabor.3.0.2.mag.
6450	T2W.27.gabor.3.0.3.mean.

6451	T2W.27.gabor.3.0.3.var.
6452	T2W.27.gabor.3.0.3.absmean.
6453	T2W.27.gabor.3.0.3.mag.
6454	T2W.27.gabor.3.0.4.mean.
6455	T2W.27.gabor.3.0.4.var.
6456	T2W.27.gabor.3.0.4.absmean.
6457	T2W.27.gabor.3.0.4.mag.
6458	T2W.27.gabor.3.0.5.mean.
6459	T2W.27.gabor.3.0.5.var.
6460	T2W.27.gabor.3.0.5.absmean.
6461	T2W.27.gabor.3.0.5.mag.
6462	T2W.31.gabor.1.0.1.mean.
6463	T2W.31.gabor.1.0.1.var.
6464	T2W.31.gabor.1.0.1.absmean.
6465	T2W.31.gabor.1.0.1.mag.
6466	T2W.31.gabor.1.0.2.mean.
6467	T2W.31.gabor.1.0.2.var.
6468	T2W.31.gabor.1.0.2.absmean.
6469	T2W.31.gabor.1.0.2.mag.
6470	T2W.31.gabor.1.0.3.mean.
6471	T2W.31.gabor.1.0.3.var.
6472	T2W.31.gabor.1.0.3.absmean.
6473	T2W.31.gabor.1.0.3.mag.
6474	T2W.31.gabor.1.0.4.mean.
6475	T2W.31.gabor.1.0.4.var.
6476	T2W.31.gabor.1.0.4.absmean.
6477	T2W.31.gabor.1.0.4.mag.
6478	T2W.31.gabor.1.0.5.mean.
6479	T2W.31.gabor.1.0.5.var.
6480	T2W.31.gabor.1.0.5.absmean.
6481	T2W.31.gabor.1.0.5.mag.
6482	T2W.31.gabor.2.0.1.mean.
6483	T2W.31.gabor.2.0.1.var.
6484	T2W.31.gabor.2.0.1.absmean.
6485	T2W.31.gabor.2.0.1.mag.
6486	T2W.31.gabor.2.0.2.mean.
6487	T2W.31.gabor.2.0.2.var.

6488	T2W.31.gabor.2.0.2.absmean.
6489	T2W.31.gabor.2.0.2.mag.
6490	T2W.31.gabor.2.0.3.mean.
6491	T2W.31.gabor.2.0.3.var.
6492	T2W.31.gabor.2.0.3.absmean.
6493	T2W.31.gabor.2.0.3.mag.
6494	T2W.31.gabor.2.0.4.mean.
6495	T2W.31.gabor.2.0.4.var.
6496	T2W.31.gabor.2.0.4.absmean.
6497	T2W.31.gabor.2.0.4.mag.
6498	T2W.31.gabor.2.0.5.mean.
6499	T2W.31.gabor.2.0.5.var.
6500	T2W.31.gabor.2.0.5.absmean.
6501	T2W.31.gabor.2.0.5.mag.
6502	T2W.31.gabor.3.0.1.mean.
6503	T2W.31.gabor.3.0.1.var.
6504	T2W.31.gabor.3.0.1.absmean.
6505	T2W.31.gabor.3.0.1.mag.
6506	T2W.31.gabor.3.0.2.mean.
6507	T2W.31.gabor.3.0.2.var.
6508	T2W.31.gabor.3.0.2.absmean.
6509	T2W.31.gabor.3.0.2.mag.
6510	T2W.31.gabor.3.0.3.mean.
6511	T2W.31.gabor.3.0.3.var.
6512	T2W.31.gabor.3.0.3.absmean.
6513	T2W.31.gabor.3.0.3.mag.
6514	T2W.31.gabor.3.0.4.mean.
6515	T2W.31.gabor.3.0.4.var.
6516	T2W.31.gabor.3.0.4.absmean.
6517	T2W.31.gabor.3.0.4.mag.
6518	T2W.31.gabor.3.0.5.mean.
6519	T2W.31.gabor.3.0.5.var.
6520	T2W.31.gabor.3.0.5.absmean.
6521	T2W.31.gabor.3.0.5.mag.
6522	T2W.35.gabor.1.0.1.mean.
6523	T2W.35.gabor.1.0.1.var.
6524	T2W.35.gabor.1.0.1.absmean.

6525	T2W.35.gabor.1.0.1.mag.
6526	T2W.35.gabor.1.0.2.mean.
6527	T2W.35.gabor.1.0.2.var.
6528	T2W.35.gabor.1.0.2.absmean.
6529	T2W.35.gabor.1.0.2.mag.
6530	T2W.35.gabor.1.0.3.mean.
6531	T2W.35.gabor.1.0.3.var.
6532	T2W.35.gabor.1.0.3.absmean.
6533	T2W.35.gabor.1.0.3.mag.
6534	T2W.35.gabor.1.0.4.mean.
6535	T2W.35.gabor.1.0.4.var.
6536	T2W.35.gabor.1.0.4.absmean.
6537	T2W.35.gabor.1.0.4.mag.
6538	T2W.35.gabor.1.0.5.mean.
6539	T2W.35.gabor.1.0.5.var.
6540	T2W.35.gabor.1.0.5.absmean.
6541	T2W.35.gabor.1.0.5.mag.
6542	T2W.35.gabor.2.0.1.mean.
6543	T2W.35.gabor.2.0.1.var.
6544	T2W.35.gabor.2.0.1.absmean.
6545	T2W.35.gabor.2.0.1.mag.
6546	T2W.35.gabor.2.0.2.mean.
6547	T2W.35.gabor.2.0.2.var.
6548	T2W.35.gabor.2.0.2.absmean.
6549	T2W.35.gabor.2.0.2.mag.
6550	T2W.35.gabor.2.0.3.mean.
6551	T2W.35.gabor.2.0.3.var.
6552	T2W.35.gabor.2.0.3.absmean.
6553	T2W.35.gabor.2.0.3.mag.
6554	T2W.35.gabor.2.0.4.mean.
6555	T2W.35.gabor.2.0.4.var.
6556	T2W.35.gabor.2.0.4.absmean.
6557	T2W.35.gabor.2.0.4.mag.
6558	T2W.35.gabor.2.0.5.mean.
6559	T2W.35.gabor.2.0.5.var.
6560	T2W.35.gabor.2.0.5.absmean.
6561	T2W.35.gabor.2.0.5.mag.

6562	T2W.35.gabor.3.0.1.mean.
6563	T2W.35.gabor.3.0.1.var.
6564	T2W.35.gabor.3.0.1.absmean.
6565	T2W.35.gabor.3.0.1.mag.
6566	T2W.35.gabor.3.0.2.mean.
6567	T2W.35.gabor.3.0.2.var.
6568	T2W.35.gabor.3.0.2.absmean.
6569	T2W.35.gabor.3.0.2.mag.
6570	T2W.35.gabor.3.0.3.mean.
6571	T2W.35.gabor.3.0.3.var.
6572	T2W.35.gabor.3.0.3.absmean.
6573	T2W.35.gabor.3.0.3.mag.
6574	T2W.35.gabor.3.0.4.mean.
6575	T2W.35.gabor.3.0.4.var.
6576	T2W.35.gabor.3.0.4.absmean.
6577	T2W.35.gabor.3.0.4.mag.
6578	T2W.35.gabor.3.0.5.mean.
6579	T2W.35.gabor.3.0.5.var.
6580	T2W.35.gabor.3.0.5.absmean.
6581	T2W.35.gabor.3.0.5.mag.
6582	T2W.3.haar.1.1.aav.
6583	T2W.3.haar.1.1.std.
6584	T2W.3.haar.1.2.aav.
6585	T2W.3.haar.1.2.std.
6586	T2W.3.haar.1.3.aav.
6587	T2W.3.haar.1.3.std.
6588	T2W.3.haar.2.1.aav.
6589	T2W.3.haar.2.1.std.
6590	T2W.3.haar.2.2.aav.
6591	T2W.3.haar.2.2.std.
6592	T2W.3.haar.2.3.aav.
6593	T2W.3.haar.2.3.std.
6594	T2W.3.haar.3.1.aav.
6595	T2W.3.haar.3.1.std.
6596	T2W.3.haar.3.2.aav.
6597	T2W.3.haar.3.2.std.
6598	T2W.3.haar.3.3.aav.

6599	T2W.3.haar.3.3.std.
6600	T2W.3.haar.4.1.aav.
6601	T2W.3.haar.4.1.std.
6602	T2W.3.haar.4.2.aav.
6603	T2W.3.haar.4.2.std.
6604	T2W.3.haar.4.3.aav.
6605	T2W.3.haar.4.3.std.
6606	T2W.7.haar.1.1.aav.
6607	T2W.7.haar.1.1.std.
6608	T2W.7.haar.1.2.aav.
6609	T2W.7.haar.1.2.std.
6610	T2W.7.haar.1.3.aav.
6611	T2W.7.haar.1.3.std.
6612	T2W.7.haar.2.1.aav.
6613	T2W.7.haar.2.1.std.
6614	T2W.7.haar.2.2.aav.
6615	T2W.7.haar.2.2.std.
6616	T2W.7.haar.2.3.aav.
6617	T2W.7.haar.2.3.std.
6618	T2W.7.haar.3.1.aav.
6619	T2W.7.haar.3.1.std.
6620	T2W.7.haar.3.2.aav.
6621	T2W.7.haar.3.2.std.
6622	T2W.7.haar.3.3.aav.
6623	T2W.7.haar.3.3.std.
6624	T2W.7.haar.4.1.aav.
6625	T2W.7.haar.4.1.std.
6626	T2W.7.haar.4.2.aav.
6627	T2W.7.haar.4.2.std.
6628	T2W.7.haar.4.3.aav.
6629	T2W.7.haar.4.3.std.
6630	T2W.11.haar.1.1.aav.
6631	T2W.11.haar.1.1.std.
6632	T2W.11.haar.1.2.aav.
6633	T2W.11.haar.1.2.std.
6634	T2W.11.haar.1.3.aav.
6635	T2W.11.haar.1.3.std.

6636	T2W.11.haar.2.1.aav.
6637	T2W.11.haar.2.1.std.
6638	T2W.11.haar.2.2.aav.
6639	T2W.11.haar.2.2.std.
6640	T2W.11.haar.2.3.aav.
6641	T2W.11.haar.2.3.std.
6642	T2W.11.haar.3.1.aav.
6643	T2W.11.haar.3.1.std.
6644	T2W.11.haar.3.2.aav.
6645	T2W.11.haar.3.2.std.
6646	T2W.11.haar.3.3.aav.
6647	T2W.11.haar.3.3.std.
6648	T2W.11.haar.4.1.aav.
6649	T2W.11.haar.4.1.std.
6650	T2W.11.haar.4.2.aav.
6651	T2W.11.haar.4.2.std.
6652	T2W.11.haar.4.3.aav.
6653	T2W.11.haar.4.3.std.
6654	T2W.15.haar.1.1.aav.
6655	T2W.15.haar.1.1.std.
6656	T2W.15.haar.1.2.aav.
6657	T2W.15.haar.1.2.std.
6658	T2W.15.haar.1.3.aav.
6659	T2W.15.haar.1.3.std.
6660	T2W.15.haar.2.1.aav.
6661	T2W.15.haar.2.1.std.
6662	T2W.15.haar.2.2.aav.
6663	T2W.15.haar.2.2.std.
6664	T2W.15.haar.2.3.aav.
6665	T2W.15.haar.2.3.std.
6666	T2W.15.haar.3.1.aav.
6667	T2W.15.haar.3.1.std.
6668	T2W.15.haar.3.2.aav.
6669	T2W.15.haar.3.2.std.
6670	T2W.15.haar.3.3.aav.
6671	T2W.15.haar.3.3.std.
6672	T2W.15.haar.4.1.aav.

6673	T2W.15.haar.4.1.std.
6674	T2W.15.haar.4.2.aav.
6675	T2W.15.haar.4.2.std.
6676	T2W.15.haar.4.3.aav.
6677	T2W.15.haar.4.3.std.
6678	T2W.19.haar.1.1.aav.
6679	T2W.19.haar.1.1.std.
6680	T2W.19.haar.1.2.aav.
6681	T2W.19.haar.1.2.std.
6682	T2W.19.haar.1.3.aav.
6683	T2W.19.haar.1.3.std.
6684	T2W.19.haar.2.1.aav.
6685	T2W.19.haar.2.1.std.
6686	T2W.19.haar.2.2.aav.
6687	T2W.19.haar.2.2.std.
6688	T2W.19.haar.2.3.aav.
6689	T2W.19.haar.2.3.std.
6690	T2W.19.haar.3.1.aav.
6691	T2W.19.haar.3.1.std.
6692	T2W.19.haar.3.2.aav.
6693	T2W.19.haar.3.2.std.
6694	T2W.19.haar.3.3.aav.
6695	T2W.19.haar.3.3.std.
6696	T2W.19.haar.4.1.aav.
6697	T2W.19.haar.4.1.std.
6698	T2W.19.haar.4.2.aav.
6699	T2W.19.haar.4.2.std.
6700	T2W.19.haar.4.3.aav.
6701	T2W.19.haar.4.3.std.
6702	T2W.23.haar.1.1.aav.
6703	T2W.23.haar.1.1.std.
6704	T2W.23.haar.1.2.aav.
6705	T2W.23.haar.1.2.std.
6706	T2W.23.haar.1.3.aav.
6707	T2W.23.haar.1.3.std.
6708	T2W.23.haar.2.1.aav.
6709	T2W.23.haar.2.1.std.

6710	T2W.23.haar.2.2.aav.
6711	T2W.23.haar.2.2.std.
6712	T2W.23.haar.2.3.aav.
6713	T2W.23.haar.2.3.std.
6714	T2W.23.haar.3.1.aav.
6715	T2W.23.haar.3.1.std.
6716	T2W.23.haar.3.2.aav.
6717	T2W.23.haar.3.2.std.
6718	T2W.23.haar.3.3.aav.
6719	T2W.23.haar.3.3.std.
6720	T2W.23.haar.4.1.aav.
6721	T2W.23.haar.4.1.std.
6722	T2W.23.haar.4.2.aav.
6723	T2W.23.haar.4.2.std.
6724	T2W.23.haar.4.3.aav.
6725	T2W.23.haar.4.3.std.
6726	T2W.27.haar.1.1.aav.
6727	T2W.27.haar.1.1.std.
6728	T2W.27.haar.1.2.aav.
6729	T2W.27.haar.1.2.std.
6730	T2W.27.haar.1.3.aav.
6731	T2W.27.haar.1.3.std.
6732	T2W.27.haar.2.1.aav.
6733	T2W.27.haar.2.1.std.
6734	T2W.27.haar.2.2.aav.
6735	T2W.27.haar.2.2.std.
6736	T2W.27.haar.2.3.aav.
6737	T2W.27.haar.2.3.std.
6738	T2W.27.haar.3.1.aav.
6739	T2W.27.haar.3.1.std.
6740	T2W.27.haar.3.2.aav.
6741	T2W.27.haar.3.2.std.
6742	T2W.27.haar.3.3.aav.
6743	T2W.27.haar.3.3.std.
6744	T2W.27.haar.4.1.aav.
6745	T2W.27.haar.4.1.std.
6746	T2W.27.haar.4.2.aav.

6747	T2W.27.haar.4.2.std.
6748	T2W.27.haar.4.3.aav.
6749	T2W.27.haar.4.3.std.
6750	T2W.31.haar.1.1.aav.
6751	T2W.31.haar.1.1.std.
6752	T2W.31.haar.1.2.aav.
6753	T2W.31.haar.1.2.std.
6754	T2W.31.haar.1.3.aav.
6755	T2W.31.haar.1.3.std.
6756	T2W.31.haar.2.1.aav.
6757	T2W.31.haar.2.1.std.
6758	T2W.31.haar.2.2.aav.
6759	T2W.31.haar.2.2.std.
6760	T2W.31.haar.2.3.aav.
6761	T2W.31.haar.2.3.std.
6762	T2W.31.haar.3.1.aav.
6763	T2W.31.haar.3.1.std.
6764	T2W.31.haar.3.2.aav.
6765	T2W.31.haar.3.2.std.
6766	T2W.31.haar.3.3.aav.
6767	T2W.31.haar.3.3.std.
6768	T2W.31.haar.4.1.aav.
6769	T2W.31.haar.4.1.std.
6770	T2W.31.haar.4.2.aav.
6771	T2W.31.haar.4.2.std.
6772	T2W.31.haar.4.3.aav.
6773	T2W.31.haar.4.3.std.
6774	T2W.35.haar.1.1.aav.
6775	T2W.35.haar.1.1.std.
6776	T2W.35.haar.1.2.aav.
6777	T2W.35.haar.1.2.std.
6778	T2W.35.haar.1.3.aav.
6779	T2W.35.haar.1.3.std.
6780	T2W.35.haar.2.1.aav.
6781	T2W.35.haar.2.1.std.
6782	T2W.35.haar.2.2.aav.
6783	T2W.35.haar.2.2.std.

6784	T2W.35.haar.2.3.aav.
6785	T2W.35.haar.2.3.std.
6786	T2W.35.haar.3.1.aav.
6787	T2W.35.haar.3.1.std.
6788	T2W.35.haar.3.2.aav.
6789	T2W.35.haar.3.2.std.
6790	T2W.35.haar.3.3.aav.
6791	T2W.35.haar.3.3.std.
6792	T2W.35.haar.4.1.aav.
6793	T2W.35.haar.4.1.std.
6794	T2W.35.haar.4.2.aav.
6795	T2W.35.haar.4.2.std.
6796	T2W.35.haar.4.3.aav.
6797	T2W.35.haar.4.3.std.
6798	T2W.3.hu.0.
6799	T2W.3.hu.1.
6800	T2W.3.hu.2.
6801	T2W.3.hu.3.
6802	T2W.3.hu.4.
6803	T2W.3.hu.5.
6804	T2W.3.hu.6.
6805	T2W.7.hu.0.
6806	T2W.7.hu.1.
6807	T2W.7.hu.2.
6808	T2W.7.hu.3.
6809	T2W.7.hu.4.
6810	T2W.7.hu.5.
6811	T2W.7.hu.6.
6812	T2W.11.hu.0.
6813	T2W.11.hu.1.
6814	T2W.11.hu.2.
6815	T2W.11.hu.3.
6816	T2W.11.hu.4.
6817	T2W.11.hu.5.
6818	T2W.11.hu.6.
6819	T2W.15.hu.0.
6820	T2W.15.hu.1.

6821	T2W.15.hu.2.
6822	T2W.15.hu.3.
6823	T2W.15.hu.4.
6824	T2W.15.hu.5.
6825	T2W.15.hu.6.
6826	T2W.19.hu.0.
6827	T2W.19.hu.1.
6828	T2W.19.hu.2.
6829	T2W.19.hu.3.
6830	T2W.19.hu.4.
6831	T2W.19.hu.5.
6832	T2W.19.hu.6.
6833	T2W.23.hu.0.
6834	T2W.23.hu.1.
6835	T2W.23.hu.2.
6836	T2W.23.hu.3.
6837	T2W.23.hu.4.
6838	T2W.23.hu.5.
6839	T2W.23.hu.6.
6840	T2W.27.hu.0.
6841	T2W.27.hu.1.
6842	T2W.27.hu.2.
6843	T2W.27.hu.3.
6844	T2W.27.hu.4.
6845	T2W.27.hu.5.
6846	T2W.27.hu.6.
6847	T2W.31.hu.0.
6848	T2W.31.hu.1.
6849	T2W.31.hu.2.
6850	T2W.31.hu.3.
6851	T2W.31.hu.4.
6852	T2W.31.hu.5.
6853	T2W.31.hu.6.
6854	T2W.35.hu.0.
6855	T2W.35.hu.1.
6856	T2W.35.hu.2.
6857	T2W.35.hu.3.

6858	T2W.35.hu.4.
6859	T2W.35.hu.5.
6860	T2W.35.hu.6.
6861	T2W.3.zernike.0.
6862	T2W.3.zernike.1.
6863	T2W.3.zernike.2.
6864	T2W.3.zernike.3.
6865	T2W.3.zernike.4.
6866	T2W.3.zernike.5.
6867	T2W.3.zernike.6.
6868	T2W.3.zernike.7.
6869	T2W.3.zernike.8.
6870	T2W.3.zernike.9.
6871	T2W.3.zernike.10.
6872	T2W.3.zernike.11.
6873	T2W.3.zernike.12.
6874	T2W.3.zernike.13.
6875	T2W.3.zernike.14.
6876	T2W.3.zernike.15.
6877	T2W.3.zernike.16.
6878	T2W.3.zernike.17.
6879	T2W.3.zernike.18.
6880	T2W.3.zernike.19.
6881	T2W.3.zernike.20.
6882	T2W.3.zernike.21.
6883	T2W.3.zernike.22.
6884	T2W.3.zernike.23.
6885	T2W.3.zernike.24.
6886	T2W.7.zernike.0.
6887	T2W.7.zernike.1.
6888	T2W.7.zernike.2.
6889	T2W.7.zernike.3.
6890	T2W.7.zernike.4.
6891	T2W.7.zernike.5.
6892	T2W.7.zernike.6.
6893	T2W.7.zernike.7.
6894	T2W.7.zernike.8.

6895	T2W.7.zernike.9.
6896	T2W.7.zernike.10.
6897	T2W.7.zernike.11.
6898	T2W.7.zernike.12.
6899	T2W.7.zernike.13.
6900	T2W.7.zernike.14.
6901	T2W.7.zernike.15.
6902	T2W.7.zernike.16.
6903	T2W.7.zernike.17.
6904	T2W.7.zernike.18.
6905	T2W.7.zernike.19.
6906	T2W.7.zernike.20.
6907	T2W.7.zernike.21.
6908	T2W.7.zernike.22.
6909	T2W.7.zernike.23.
6910	T2W.7.zernike.24.
6911	T2W.11.zernike.0.
6912	T2W.11.zernike.1.
6913	T2W.11.zernike.2.
6914	T2W.11.zernike.3.
6915	T2W.11.zernike.4.
6916	T2W.11.zernike.5.
6917	T2W.11.zernike.6.
6918	T2W.11.zernike.7.
6919	T2W.11.zernike.8.
6920	T2W.11.zernike.9.
6921	T2W.11.zernike.10.
6922	T2W.11.zernike.11.
6923	T2W.11.zernike.12.
6924	T2W.11.zernike.13.
6925	T2W.11.zernike.14.
6926	T2W.11.zernike.15.
6927	T2W.11.zernike.16.
6928	T2W.11.zernike.17.
6929	T2W.11.zernike.18.
6930	T2W.11.zernike.19.
6931	T2W.11.zernike.20.

6932	T2W.11.zernike.21.
6933	T2W.11.zernike.22.
6934	T2W.11.zernike.23.
6935	T2W.11.zernike.24.
6936	T2W.15.zernike.0.
6937	T2W.15.zernike.1.
6938	T2W.15.zernike.2.
6939	T2W.15.zernike.3.
6940	T2W.15.zernike.4.
6941	T2W.15.zernike.5.
6942	T2W.15.zernike.6.
6943	T2W.15.zernike.7.
6944	T2W.15.zernike.8.
6945	T2W.15.zernike.9.
6946	T2W.15.zernike.10.
6947	T2W.15.zernike.11.
6948	T2W.15.zernike.12.
6949	T2W.15.zernike.13.
6950	T2W.15.zernike.14.
6951	T2W.15.zernike.15.
6952	T2W.15.zernike.16.
6953	T2W.15.zernike.17.
6954	T2W.15.zernike.18.
6955	T2W.15.zernike.19.
6956	T2W.15.zernike.20.
6957	T2W.15.zernike.21.
6958	T2W.15.zernike.22.
6959	T2W.15.zernike.23.
6960	T2W.15.zernike.24.
6961	T2W.19.zernike.0.
6962	T2W.19.zernike.1.
6963	T2W.19.zernike.2.
6964	T2W.19.zernike.3.
6965	T2W.19.zernike.4.
6966	T2W.19.zernike.5.
6967	T2W.19.zernike.6.
6968	T2W.19.zernike.7.

6969	T2W.19.zernike.8.
6970	T2W.19.zernike.9.
6971	T2W.19.zernike.10.
6972	T2W.19.zernike.11.
6973	T2W.19.zernike.12.
6974	T2W.19.zernike.13.
6975	T2W.19.zernike.14.
6976	T2W.19.zernike.15.
6977	T2W.19.zernike.16.
6978	T2W.19.zernike.17.
6979	T2W.19.zernike.18.
6980	T2W.19.zernike.19.
6981	T2W.19.zernike.20.
6982	T2W.19.zernike.21.
6983	T2W.19.zernike.22.
6984	T2W.19.zernike.23.
6985	T2W.19.zernike.24.
6986	T2W.23.zernike.0.
6987	T2W.23.zernike.1.
6988	T2W.23.zernike.2.
6989	T2W.23.zernike.3.
6990	T2W.23.zernike.4.
6991	T2W.23.zernike.5.
6992	T2W.23.zernike.6.
6993	T2W.23.zernike.7.
6994	T2W.23.zernike.8.
6995	T2W.23.zernike.9.
6996	T2W.23.zernike.10.
6997	T2W.23.zernike.11.
6998	T2W.23.zernike.12.
6999	T2W.23.zernike.13.
7000	T2W.23.zernike.14.
7001	T2W.23.zernike.15.
7002	T2W.23.zernike.16.
7003	T2W.23.zernike.17.
7004	T2W.23.zernike.18.
7005	T2W.23.zernike.19.

7006	T2W.23.zernike.20.
7007	T2W.23.zernike.21.
7008	T2W.23.zernike.22.
7009	T2W.23.zernike.23.
7010	T2W.23.zernike.24.
7011	T2W.27.zernike.0.
7012	T2W.27.zernike.1.
7013	T2W.27.zernike.2.
7014	T2W.27.zernike.3.
7015	T2W.27.zernike.4.
7016	T2W.27.zernike.5.
7017	T2W.27.zernike.6.
7018	T2W.27.zernike.7.
7019	T2W.27.zernike.8.
7020	T2W.27.zernike.9.
7021	T2W.27.zernike.10.
7022	T2W.27.zernike.11.
7023	T2W.27.zernike.12.
7024	T2W.27.zernike.13.
7025	T2W.27.zernike.14.
7026	T2W.27.zernike.15.
7027	T2W.27.zernike.16.
7028	T2W.27.zernike.17.
7029	T2W.27.zernike.18.
7030	T2W.27.zernike.19.
7031	T2W.27.zernike.20.
7032	T2W.27.zernike.21.
7033	T2W.27.zernike.22.
7034	T2W.27.zernike.23.
7035	T2W.27.zernike.24.
7036	T2W.31.zernike.0.
7037	T2W.31.zernike.1.
7038	T2W.31.zernike.2.
7039	T2W.31.zernike.3.
7040	T2W.31.zernike.4.
7041	T2W.31.zernike.5.
7042	T2W.31.zernike.6.

7043	T2W.31.zernike.7.
7044	T2W.31.zernike.8.
7045	T2W.31.zernike.9.
7046	T2W.31.zernike.10.
7047	T2W.31.zernike.11.
7048	T2W.31.zernike.12.
7049	T2W.31.zernike.13.
7050	T2W.31.zernike.14.
7051	T2W.31.zernike.15.
7052	T2W.31.zernike.16.
7053	T2W.31.zernike.17.
7054	T2W.31.zernike.18.
7055	T2W.31.zernike.19.
7056	T2W.31.zernike.20.
7057	T2W.31.zernike.21.
7058	T2W.31.zernike.22.
7059	T2W.31.zernike.23.
7060	T2W.31.zernike.24.
7061	T2W.35.zernike.0.
7062	T2W.35.zernike.1.
7063	T2W.35.zernike.2.
7064	T2W.35.zernike.3.
7065	T2W.35.zernike.4.
7066	T2W.35.zernike.5.
7067	T2W.35.zernike.6.
7068	T2W.35.zernike.7.
7069	T2W.35.zernike.8.
7070	T2W.35.zernike.9.
7071	T2W.35.zernike.10.
7072	T2W.35.zernike.11.
7073	T2W.35.zernike.12.
7074	T2W.35.zernike.13.
7075	T2W.35.zernike.14.
7076	T2W.35.zernike.15.
7077	T2W.35.zernike.16.
7078	T2W.35.zernike.17.
7079	T2W.35.zernike.18.

7080	T2W.35.zernike.19.
7081	T2W.35.zernike.20.
7082	T2W.35.zernike.21.
7083	T2W.35.zernike.22.
7084	T2W.35.zernike.23.
7085	T2W.35.zernike.24.
7086	T2W.3.sobel
7087	T2W.3.sobel_mask
7088	T2W.all.stats.p000.
7089	T2W.all.stats.p010.
7090	T2W.all.stats.p020.
7091	T2W.all.stats.p025.
7092	T2W.all.stats.p030.
7093	T2W.all.stats.p040.
7094	T2W.all.stats.p050.
7095	T2W.all.stats.p060.
7096	T2W.all.stats.p070.
7097	T2W.all.stats.p075.
7098	T2W.all.stats.p080.
7099	T2W.all.stats.p090.
7100	T2W.all.stats.p100.
7101	T2W.all.stats.range.
7102	T2W.all.stats.mean.
7103	T2W.all.stats.stddev.
7104	T2W.all.stats.kurtosis.
7105	T2W.all.stats.skewness.

Supplementary Table S3. Showing the parameters of the feature selections used in this study

Feature Selection	Parameters
Recursive Feature Elimination (RFE)	Linear regression estimator, 10-fold cross-validation, number of features selected depends on the number of features set and ranked based on the performance metric (root mean squared error (RMSE)) based on cross-validation.

LASSO	10 fold cross validation, regularization parameter using minimum cross-validation error criterion
MRMR	Mutual information (MI) as relevance measure, conditional mutual information (CMI) as redundancy measure, and a weighted sum method to combine measures

Supplementary Table S4. Showing the parameters of the LASSO and Random Forest models

Model	Parameters	Evaluation metric
LASSO	Lambda = lambda min*, 5 and 10-fold CV	AUC
Random Forest	1000 trees, max node=20, mtry=1-20,5 and10-fold CV	Accuracy

*Lambda min was obtained by using the cv.glmnet() function in R to fit a LASSO regression model with elastic net regularization. 10-fold cross-validation to select theca optimal value of the tuning parameter lambda, which controls the strength of the L1 regularization penalty. The lamda.min value which corresponds to teh minimum cross-validated error, was then chosen as the optimal value of lambda.

Supplementary Table S5. Showing the indices of robust features selected by 3 different feature selection in each datasets with different ratio of pertubed endpoints

Dataset	Ratio of added noise	Robust Features selected		
		RFE	LASSO	MRMR
Dataset 1 (threshold = 20)	0%	142, 133, 146, 144, 109, 230, 242, 18, 147, 103, 28, 56, 55, 104	78, 144, 103, 254, 14, 4, 166, 189	14, 27, 28, 29, 37, 55, 73, 74, 75, 103, 133, 137, 139, 142, 143, 144, 146, 147, 148
	5%	142, 133, 146, 144, 230, 147	143, 37, 86, 71, 174, 262, 78, 97	27, 29, 37, 74, 98, 103, 133, 137, 138, 142, 143, 144, 146, 147, 148, 190
	10%	142, 133, 146, 144, 230, 147	143, 37, 86, 174, 262, 78, 97	27, 29, 37, 74, 98, 103, 133, 137, 138, 142, 143, 144, 146, 147, 148
	20%	142, 133, 146, 144, 230	143, 86, 174, 262, 78	27, 29, 37, 74, 98, 103, 133, 137, 138, 142, 143, 144, 146, 147, 148
	30%	142, 133, 146, 144, 230	143, 37, 86, 174, 78, 97	27, 29, 37, 74, 103, 133, 137, 138, 142, 143, 144, 146, 147, 148
	40%	142, 133, 146, 144, 147, 230	143, 86, 174, 262	27, 29, 37, 74, 103, 133, 137, 138, 142, 143, 144, 146, 147, 148

	50%	142, 133, 146, 144	143, 86, 174, 262	27, 29, 37, 74, 98, 103, 133, 137, 142, 143, 144, 146, 147, 148
	60%	142, 133, 146, 144, 230, 147	143, 86, 97	27, 29, 37, 74, 98, 103, 133, 137, 138, 142, 143, 144, 146, 147, 148
	70%	142, 133, 146, 144	143	27, 29, 37, 74, 103, 133, 137, 138, 142, 143, 144, 146, 147, 148
	80%	142, 133, 146, 144	143, 37, 97	27, 29, 37, 74, 98, 103, 133, 137, 142, 143, 144, 146, 147, 148
	90%	142, 133, 146, 144, 147, 230	143, 86, 78, 174	27, 29, 37, 74, 103, 133, 137, 142, 143, 144, 146, 147, 148
	100%	142, 133, 146, 144	143, 78, 97	27, 29, 37, 74, 133, 137, 138, 142, 143, 144, 146, 147, 148, 190
Dataset 2 (threshold =50)	0%	142, 133, 146, 144, 109, 230, 242, 18, 147, 103, 28, 56, 55, 104	78, 144, 103, 254, 14, 4, 166, 189	14, 27, 28, 29, 37, 55, 73, 74, 75, 103, 133, 137, 139, 142, 143, 144, 146, 147, 148, 165, 203, 234, 250, 251, 190, 174, 96, 79, 166, 17, 4, 5, 78, 100, 167, 254, 255, 262, 180
	5%	142, 133, 146, 144, 230, 147	143, 37, 86, 71, 174, 262, 78, 97, 138, 190, 4	27, 28, 29, 37, 74, 98, 103, 133, 137, 138, 142, 143, 144, 146, 147, 148, 190, 203, 231, 239, 250, 251, 4, 55, 73, 79, 166, 78, 86, 174, 167, 255, 77, 254
	10%	142, 133, 146, 144, 230, 147	143, 37, 86, 174, 262, 78, 97, 103, 190, 4	4, 27, 29, 37, 55, 73, 74, 98, 103, 133, 137, 138, 142, 143, 144, 146, 147, 148, 190, 203, 231, 239, 250, 251, 28, 166, 79, 167, 78, 86, 174, 254, 255
	20%	142, 133, 146, 144, 230	143, 86, 174, 262, 78, 103, 190, 4	4, 27, 28, 29, 37, 73, 74, 98, 103, 133, 137, 138, 142, 143, 144, 146, 147, 148, 190, 203, 231, 239, 250, 251, 55, 167, 166, 174, 78, 86, 79, 254, 255
	30%	142, 133, 146, 144, 230	143, 37, 86, 174, 262, 78, 97, 103, 138, 190	4, 27, 28, 29, 37, 55, 73, 74, 98, 103, 133, 137, 138, 142, 143, 144, 146, 147, 148, 190, 203, 231, 239, 250, 251, 174, 167, 254, 78, 79, 166, 255, 86
	40%	142, 133, 146, 144, 147, 230	143, 86, 174, 262, 103, 4	27, 28, 29, 37, 55, 74, 98, 103, 133, 137, 138, 142, 143, 144, 146, 147, 148, 190, 203, 231, 239, 250, 251, 4, 174, 79, 166, 78, 254, 255, 86, 167
	50%	142, 133, 146, 144	143, 86, 174, 262, 103, 133, 4	4, 27, 28, 29, 37, 73, 74, 98, 103, 133, 137, 138, 142, 143, 144, 146, 147, 148, 190, 203, 231, 239, 250, 251, 55, 78, 165, 255, 86, 174, 167, 254, 79, 166

	60%	142, 133, 146, 144, 230, 147	143, 86, 262, 97, 103, 4	4, 27, 28, 29, 37, 55, 73, 74, 98, 103, 133, 137, 138, 142, 143, 144, 146, 147, 148, 190, 203, 231, 239, 250, 251, 165, 78, 167, 174, 79, 254, 166, 255
	70%	142, 133, 146, 144	143, 133, 190, 4	27, 28, 29, 37, 55, 74, 98, 103, 133, 137, 138, 142, 143, 144, 146, 147, 148, 190, 203, 231, 239, 250, 251, 4, 86, 78, 79, 167, 166, 165, 174, 254, 255
	80%	142, 133, 146, 144	143, 37, 97, 4	27, 28, 29, 37, 55, 74, 98, 103, 133, 137, 142, 143, 144, 146, 147, 148, 190, 203, 231, 239, 250, 251, 4, 73, 138, 253, 78, 255, 174, 254, 79, 167, 166
	90%	142, 133, 146, 144, 147, 230	143, 86, 78, 174, 262, 190	4, 27, 28, 29, 37, 74, 98, 103, 133, 137, 138, 142, 143, 144, 146, 147, 148, 190, 203, 231, 239, 250, 251, 55, 174, 166, 255, 78, 79, 167, 254, 86
	100%	142, 133, 146, 144	143, 78, 97	27, 28, 29, 37, 74, 98, 103, 133, 137, 138, 142, 143, 144, 146, 147, 148, 190, 203, 231, 239, 250, 251, 4, 55, 165, 174, 78, 166, 167, 255, 86, 79, 254
Dataset 2 (threshold = 20)	0%	2345, 5917, 5919, 3596, 642, 653, 5535, 597, 641, 3590, 649, 634, 3589, 647, 645, 3208, 2134, 6934	736, 1908, 2252, 2253, 734, 6983, 5315, 5289, 1143, 5919, 5917, 1151, 1091, 5956, 5533, 7009, 2440, 6934, 77, 3996	59, 77, 859, 1828, 2008, 2449, 2639, 2761, 2917, 3619, 3920, 5191, 5314, 5315, 5533, 5671, 5767, 5917
	5%		714, 5917	77, 345, 1265, 1434, 1768, 2917, 3010, 3948, 4104, 4152, 5202, 5205, 5219, 5314
	10%		714, 5314, 5917	77, 1434, 1768, 2917, 3948, 4104, 4152, 5205, 5219, 5314, 5383, 5917
	20%		714, 5917	77, 1768, 2917, 3948, 4152, 5205, 5314, 5383, 5917, 5919, 6424
	30%		714, 5917	77, 1768, 2917, 4152, 5205, 5314, 5383, 5917, 5919, 6424
	40%		5917	77, 1768, 2917, 5205, 5314, 5383, 5917, 5919
	50%		5917	77, 1768, 2917, 5205, 5314, 5383, 5917, 5919, 6424
	60%		714, 5917	1768, 5205, 5314, 5383, 5917, 5919
	70%		714, 5917	1768, 2917, 5205, 5314, 5917, 5919, 6424

	80%		5917	1768, 2917, 5205, 5314, 5917, 5919
	90%		5917	1768, 2917, 5205, 5314, 5917, 5919
	100%		5917	1768, 5205, 5314, 5917, 5919
Dataset 2 (threshold =50)	0%	2345, 5917, 5919, 3596, 642, 653, 5535, 597, 641, 3590, 649, 634, 3589, 647, 645, 3208, 2134, 6934, 1799, 3451, 2663, 1807, 5765, 2198, 2573, 3694, 887, 3189, 699, 6408, 2707, 1924, 2343, 853, 1870, 4128, 593, 3213, 2858, 2856, 2246, 2712	736, 1908, 2252, 2253, 734, 6983, 5315, 5289, 1143, 5919, 5917, 1151, 1091, 5956, 5533, 7009, 2440, 6934, 77, 3996, 2917, 61, 4355, 2639, 4374, 2449, 3516, 2761, 2933, 1095, 6544	59, 77, 859, 1828, 2008, 2449, 2639, 2761, 2917, 3619, 3920, 5191, 5314, 5315, 5533, 5671, 5767, 5917, 5919, 5925, 6544, 6983, 2174, 2885, 6934, 707, 875, 1151, 2615, 734, 1265, 2252, 4357, 5289, 714, 691, 694, 711, 713, 5218, 6920
	5%		714, 5917	77, 345, 1265, 1434, 1768, 2917, 3010, 3948, 4104, 4152, 4255, 5202, 5205, 5219, 5314, 5383, 5917, 5919, 5990, 6424, 7056, 835, 734, 691, 693, 694, 698, 711, 713, 714, 5211
	10%		714, 5314, 5917	77, 835, 1265, 1434, 1768, 2917, 3010, 3948, 4104, 4152, 5205, 5219, 5314, 5383, 5917, 5919, 6424, 734, 5211, 691, 693, 694, 698, 711, 713, 714
	20%		714, 5917	77, 1768, 2917, 3948, 4104, 4152, 5205, 5314, 5383, 5917, 5919, 6424, 734, 835, 691, 693, 694, 698, 711, 713, 714
	30%		714, 5917	77, 1768, 2917, 4104, 4152, 5205, 5314, 5383, 5917, 5919, 6424, 734, 691, 693, 694, 698, 711, 713, 714
	40%		5917	77, 1768, 2917, 4152, 5205, 5211, 5314, 5383, 5917, 5919, 6424, 734, 691, 693, 694, 711, 713, 714
	50%		5917	77, 1768, 2917, 5205, 5211, 5314, 5383, 5917, 5919, 6424, 734, 694, 691, 693, 698, 711, 713, 714
	60%		714, 5917	1768, 2917, 5205, 5314, 5383, 5917, 5919, 6424,

				734, 691, 693, 694, 698, 711, 713, 714
	70%		714, 5917	77, 1768, 2917, 5205, 5314, 5383, 5917, 5919, 6424, 734, 691, 693, 694, 698, 711, 713, 714
	80%		5917	77, 734, 1768, 2917, 5205, 5314, 5383, 5917, 5919, 6424, 691, 693, 694, 698, 711, 713, 714
	90%		5917	1768, 2917, 4152, 5205, 5314, 5383, 5917, 5919, 6424, 734, 691, 693, 694, 711, 713, 714
	100%		5917	1768, 2917, 4152, 5205, 5314, 5917, 5919, 6424, 734, 654, 691, 693, 694, 698, 711, 713, 714

Supplementary Figure S1. Showing the diagram to generate random features

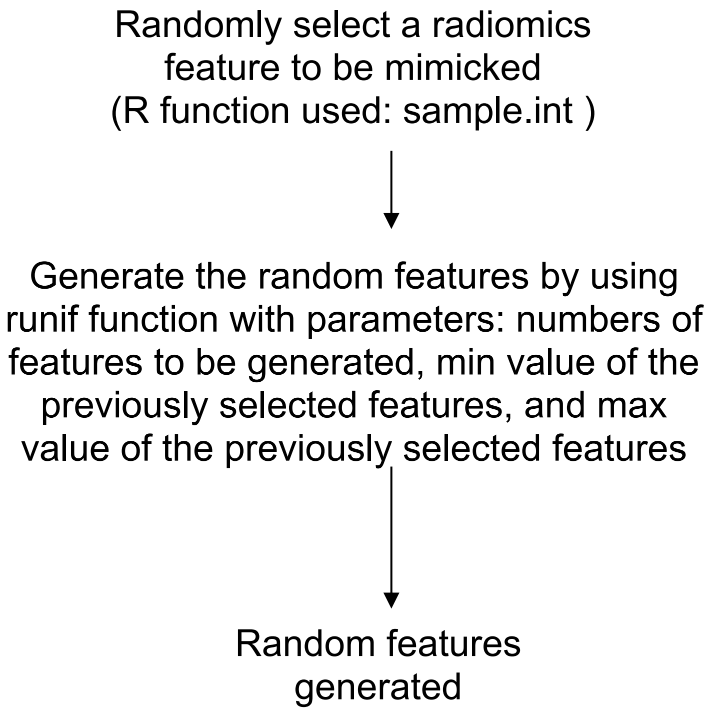

Supplementary Figure S2. showing the sensitivity of the radiomics model both in training dataset and testing dataset for dataset1 and dataset2

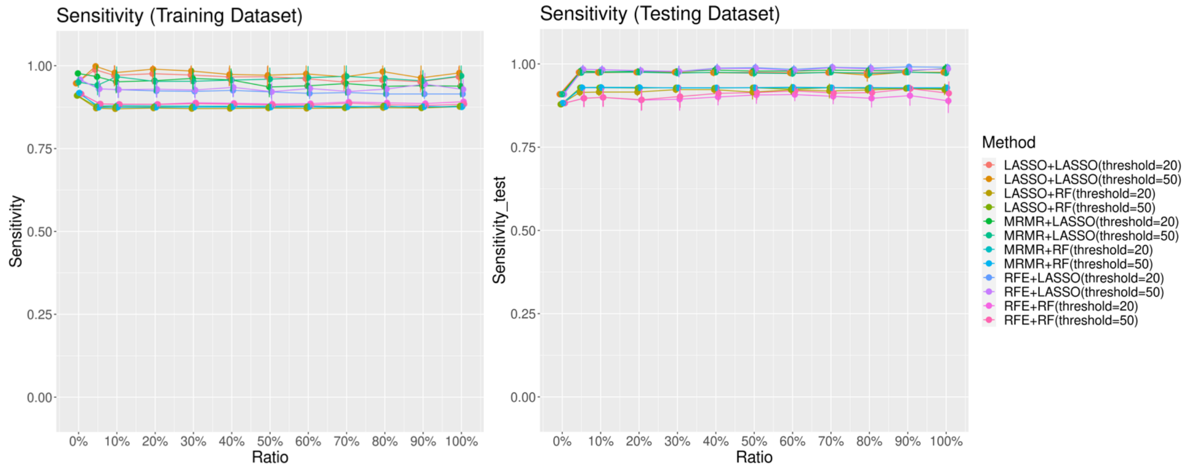

a Dataset 1

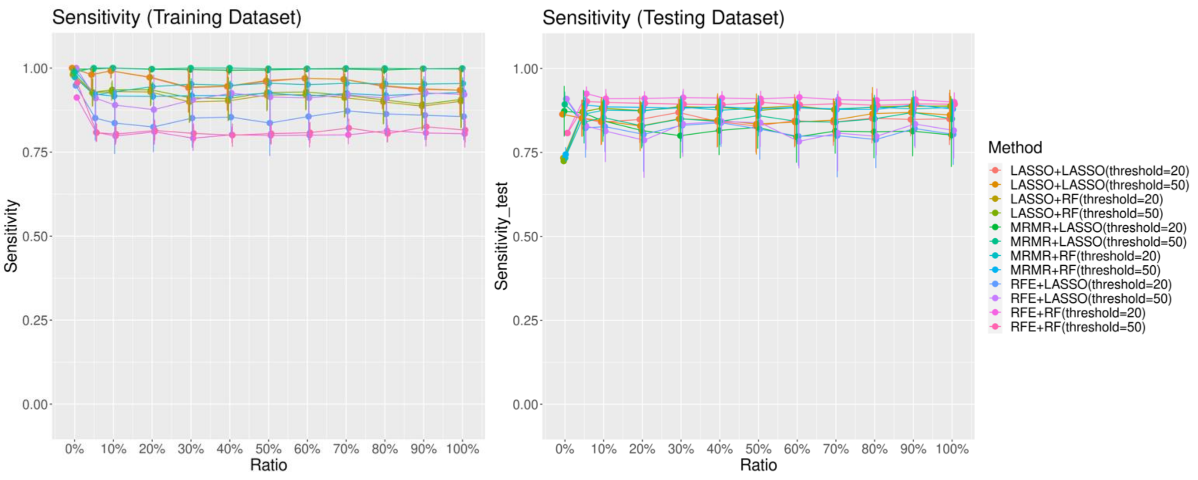

Sensitivity (Training Dataset)
Sensitivity
Ratio
Sensitivity (Testing Dataset)
Sensitivity_test
Method
LASSO+LASSO(threshold=20)
LASSO+LASSO(threshold=50)
LASSO+RF(threshold=20)
LASSO+RF(threshold=50)
MRMR+LASSO(threshold=20)
MRMR+LASSO(threshold=50)
MRMR+RF(threshold=20)
MRMR+RF(threshold=50)
RFE+LASSO(threshold=20)
RFE+LASSO(threshold=50)
RFE+RF(threshold=20)
RFE+RF(threshold=50)

b Dataset 2

Supplementary Figure S3. Showing the specificity of the radiomics model both in training dataset and testing dataset for dataset1 and dataset2

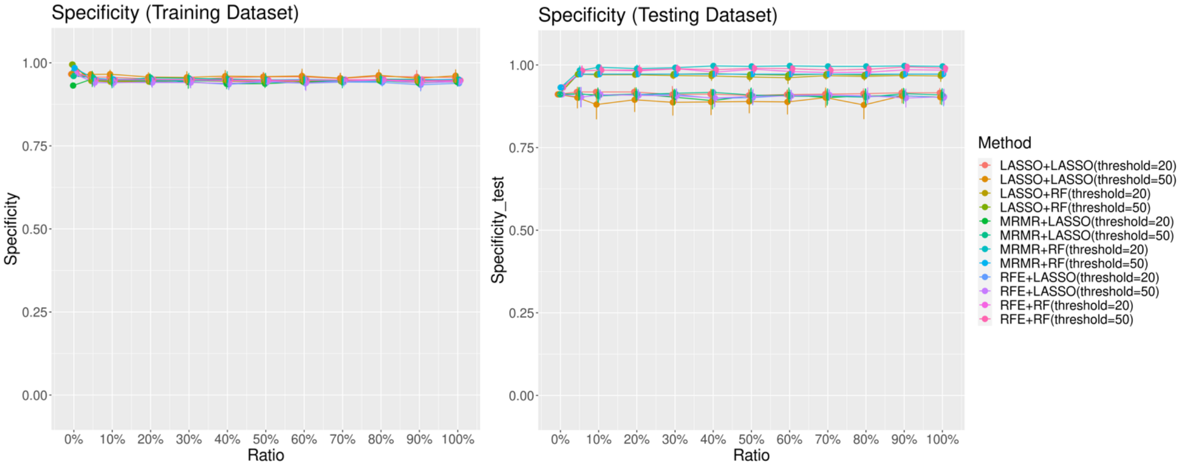

a Dataset 1

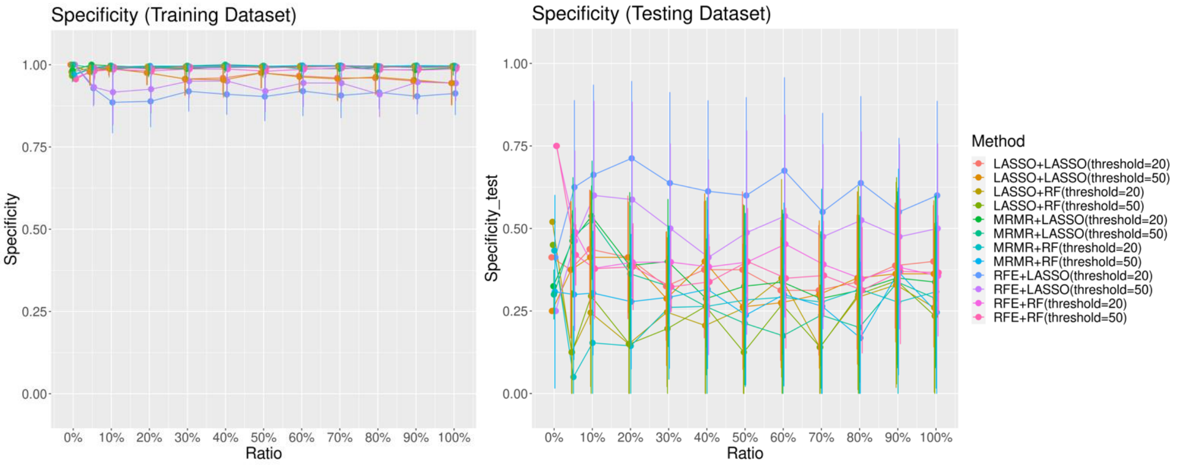

b Dataset 2

Supplementary Figure S4. Shows the importance of the top 10 most important radiomics and random features towards the radiomics model along with the total mean contribution of random features using Random Forest and LASSO classifiers for dataset 1 (a,b respectively) and dataset 2 (c,d respectively) with the maximum threshold set to 50 for the feature selection methods across every repetition and different feature selection methods for the different ratio of additional random features.

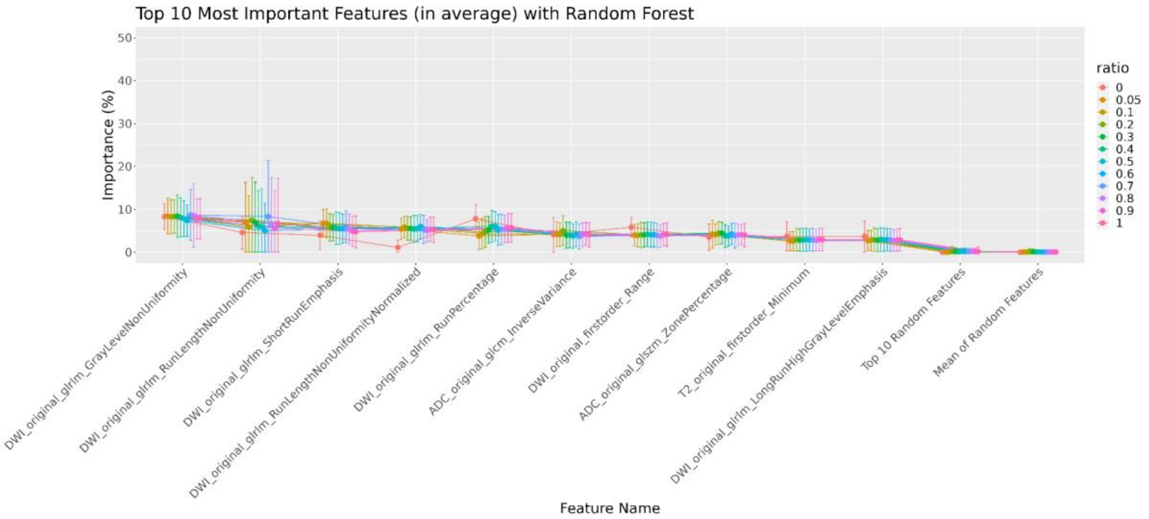

(a)

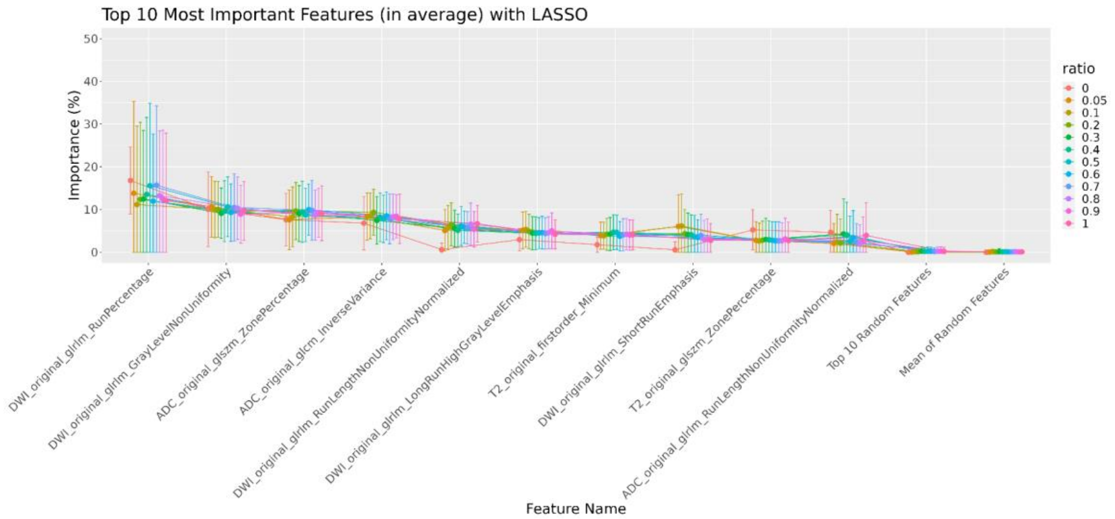

(b)

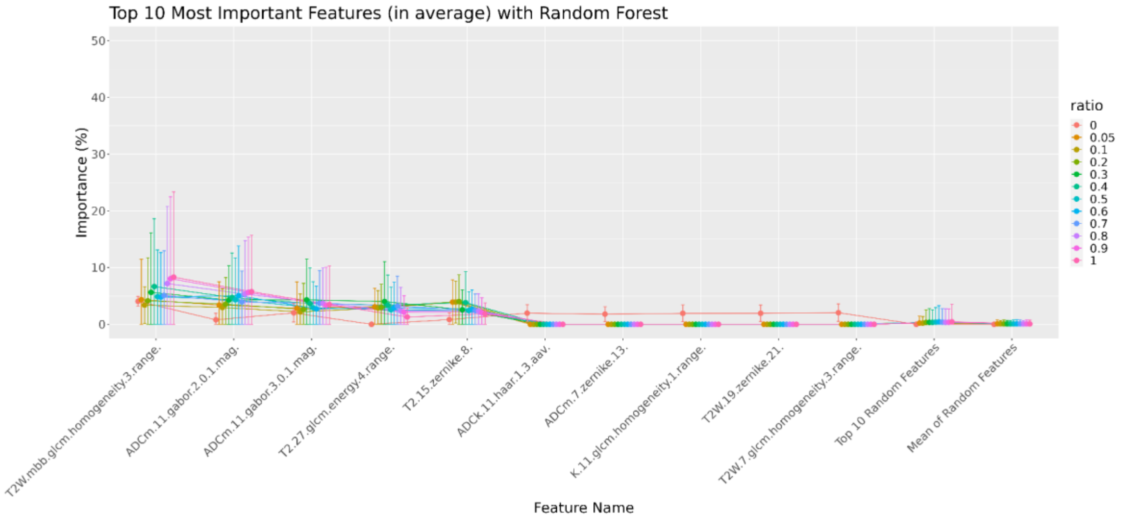

Top 10 Most Important Features (in average) with Random Forest
Importance (%)
Feature Name
ratio
0
0.05
0.1
0.2
0.3
0.4
0.5
0.6
0.7
0.8
0.9
1
T2W.mbb.glcm.homogeneity.3.range.
ADCm.11.gabor.2.0.1.mag.
ADCm.11.gabor.3.0.1.mag.
T2.27.glcm.energy.4.range.
T2.15.zernike.8.
ADCk.11.haar.1.3.aav.
ADCm.7.zernike.13.
K.11.glcm.homogeneity.1.range.
T2W.19.zernike.21.
T2W.7.glcm.homogeneity.3.range.
Top 10 Random Features
Mean of Random Features

(c)

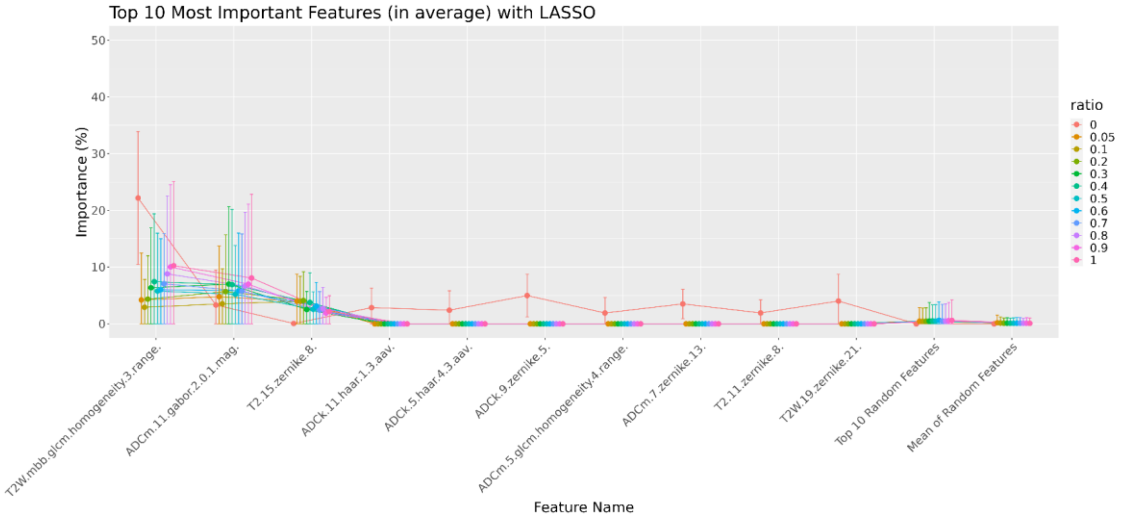

Top 10 Most Important Features (in average) with LASSO
Importance (%)
Feature Name
ratio
0
0.05
0.1
0.2
0.3
0.4
0.5
0.6
0.7
0.8
0.9
1
T2W.mbb.glcm.homogeneity.3.range.
ADCm.11.gabor.2.0.1.mag.
T2.15.zernike.8.
ADCk.11.haar.1.3.aav.
ADCk.5.haar.4.3.aav.
ADCk.9.zernike.5.
ADCm.5.glcm.homogeneity.4.range.
ADCm.7.zernike.13.
T2.11.zernike.8.
T2W.19.zernike.21.
Top 10 Random Features
Mean of Random Features

(d)
